# Identification of potential modulators of IFITM3 by in-silico modeling and virtual screening

**DOI:** 10.1038/s41598-022-20259-8

**Published:** 2022-09-24

**Authors:** Vikas Tiwari, Shruthi Viswanath

**Affiliations:** National Centre for Biological Sciences, Tata Institute of Fundamental Research, Bangalore, 560065 India

**Keywords:** Biophysics, Computational biology and bioinformatics, Drug discovery, Structural biology

## Abstract

IFITM3 is a transmembrane protein that confers innate immunity. It has been established to restrict entry of multiple viruses. Overexpression of IFITM3 has been shown to be associated with multiple cancers, implying IFITM3 to be good therapeutic target. The regulation of IFITM3 activity is mediated by multiple post-translational modifications (PTM). In this study, we have modelled the structure of IFITM3, consistent with experimental predictions on its membrane topology. MD simulation in membrane-aqueous environment revealed the stability of the model. Ligand binding sites on the IFITM3 surface were predicted and it was observed that the best site includes important residues involved in PTM and has good druggable score. Molecular docking was performed using FDA approved ligands and natural ligands from Super Natural II database. The ligands were re-ranked by calculating binding free energy. Select docking complexes were simulated again to substantiate the binding between ligand and IFITM3. We observed that known drugs like Eluxadoline and natural products like SN00224572 and Parishin A have good binding affinity against IFITM3. These ligands form persistent interactions with key lysine residues (Lys83, Lys104) and hence can potentially alter the activity of IFITM3. The results of this computational study can provide a starting point for experimental investigations on IFITM3 modulators.

## Introduction

Interferon induced transmembrane proteins (IFITMs) play an important role in various cellular processes and innate immunity upon stimulation by interferon. In humans, there are 5 members (IFITM1, IFITM2, IFITM3, IFITM5 and IFITM10) in this family of proteins with IFITM1, 2, and 3 being expressed ubiquitously while the expression of IFITM5 is restricted to osteoblasts^1–4^. IFITM proteins (IFITM1, IFITM2 or IFITM3) have been shown to restrict multiple viral infections including influenza A virus West Nile virus and dengue virus^2,5–7^. Other viruses like Marburg virus, Ebola and SARS coronavirus were also found to be differentially restricted by different IFITM proteins^8^. IFITM1 localizes predominantly to plasma membrane and early endosome while IFITM2 and IFITM3 localize to late endosome and lysosome^9^.

IFITM proteins belong to the CD225 family of proteins. IFITM proteins consist of a conserved intracellular loop (residues 81–104) between the intramembrane domain (residues 58–80) and transmembrane helix (residues 105–126)^7,10,11^. IFITM3 is a type II transmembrane protein with an intramembrane helix and C-terminus transmembrane helix. It is responsible for the majority (50–80%) of antiviral response of interferons against influenza A and residues of CD225 domain are important for viral restriction^12^. IFITM3 was shown to oligomerize and contains the GxxxG motif (residues 91–95) which is known to play an important role in oligomerization^13^. IFITM3 was shown to interact with cholesterol through residues from amphipathic helix (residues 59–68) and cholesterol binding domain (CARC motif; residues 104–113). This interaction with cholesterol is required for its antiviral activity^14,15^.

IFITM3 is regulated by several post-translational modifications that include palmitoylation, ubiquitination, methylation and phosphorylation^4^. Palmitoylation occurs at three conserved cysteine residues (Cys71, Cys72, Cys105) and mutation of these residues abolishes the antiviral activity of IFITM3^16^. Addition of myristoylation and prenylation sites at N and C-terminus of IFITM3 has been shown to rescue the antiviral function of palmitoylation deficient IFITM3 mutant^17^. E3 ubiquitin ligase NEDD4 can ubiquitinate the IFITM3 at four conserved lysine residues (K24, K83, K88, K104)^17,18^. IFITM3 is methylated at Lys88 by SET7 and this modification negatively affects antiviral activity of IFITM3^19^. The Lys88 methylation can be removed by histone demethylase LSD1 which increases the antiviral activity of IFITM3^20^. Phosphorylation of IFITM3 at Tyr20 by protein-tyrosine kinase Fyn leads to plasma membrane accumulation of IFITM3 and negatively affects the antiviral activity. Further, the Tyr20 phosphorylation or mutation of Tyr20 have been shown to decrease the ubiquitination of IFITM3^21^.

IFITM3 expression is found to be upregulated in several cancers including breast cancer, prostate cancer, colon cancer, lung cancer, gastric cancer^22–26^. Overexpression of IFITM3 has been shown to increase cancer hallmarks like cell proliferation, epithelial–mesenchymal transition and invasion. Therefore IFITM3 is a good therapeutic target to abolish tumor progression^27^. IFITM3 is also involved in Alzheimer’s disease (AD) by modulating the activity of γ-secretase. IFITM3 knockdown has been shown to decrease the amyloid plaque formation in mouse model^28^. The recent outbreak of COVID-19 by SARS CoV2 led to studies on the relation between IFITM3 and COVID-19 infection. IFITM3 expression was found to be higher in the nasopharyngeal swabs of COVID-19 patients and the expression of IFITM3 increased with infection time of COVID-19^29^. In lung epithelial cells, IFITM3 was found to be an early up-regulated gene upon SARS CoV2 infection^30^. One study showed that endogenous expression of IFITM3 promotes efficient infection by SARS CoV2^31^, while another study demonstrated that IFITM3 possesses both pro and antiviral effects against SARS CoV2^32^.

Owing to the multifactorial role of IFITM3 in multiple diseases, we have tried to find potential small molecule modulators for IFITM3 using an FDA approved dataset and a database of natural products. In this study, we have modelled the structure of IFITM3 and predicted the possible binding sites for ligand interaction followed by virtual screening and MD simulation analysis to find the best ligands for IFITM3. These results can provide a starting point for experimental investigations on IFITM3 modulators.

## Results

### IFITM3 modeled structure remains stable upon simulation

**Figure 1:**
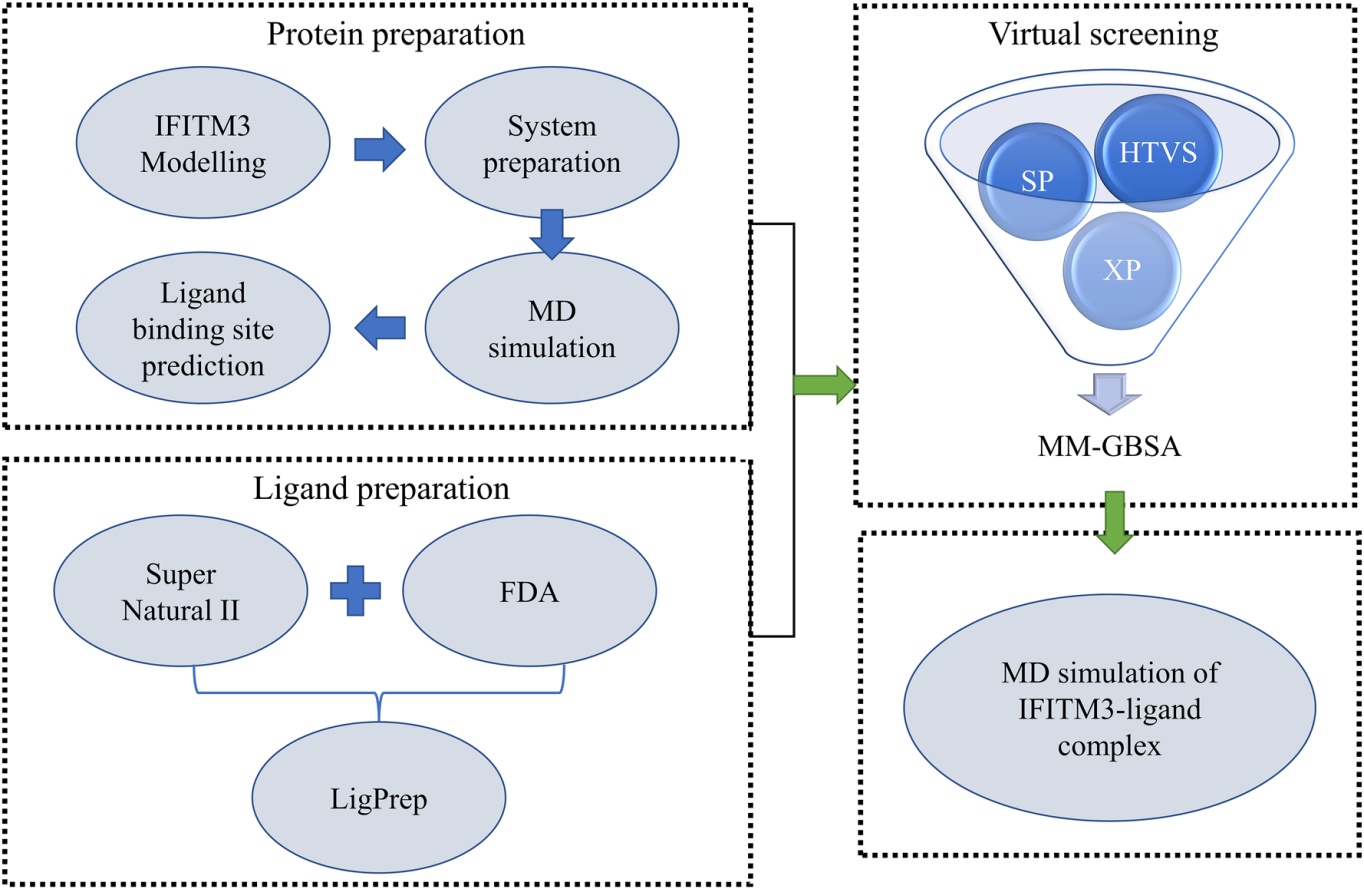
Workflow of IFITM3 modelling and virtual screening. Structure of IFITM3 was modelled followed by simulation and binding site prediction. Ligands were prepared using LigPrep module of Schrodinger. The virtual screening involved HTVS, SP and XP docking protocol. The docked complexes were subjected to MD simulation. *HTVS* high-throughput virtual screening, *SP* standard precision, *XP* extra precision.

**Figure 2:**
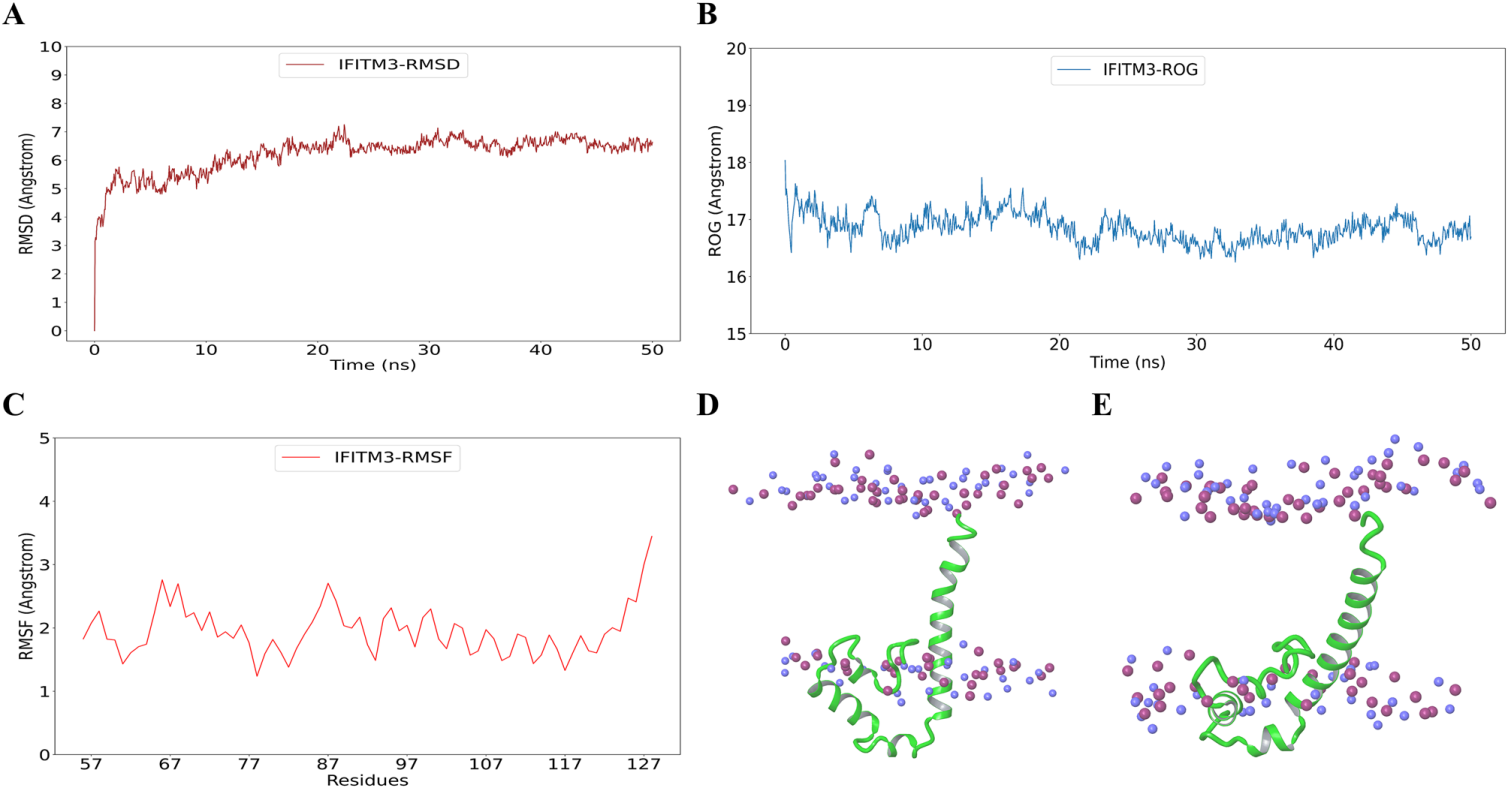
MD simulation results of IFITM3 model (**A**) RMSD of IFITM3 with respect to 0th ns (**B**) RMSF of residues across simulation time (**C**) Radius of gyration across simulation time (**D**) IFITM3 model at 0th ns (**E**) IFITM3 model at 50th ns. Blue and magenta spheres represent nitrogen and phosphorus of POPC bilayer.

The overall workflow has been summarized in Fig. 1. Detailed description of each step has been explained in methods section. Residues 56–128 of human IFITM3 were modeled, comprising of a conserved CD225 domain (consisting of an intra-membrane domain, and a conserved intracellular loop), and a transmembrane domain^7,11^. This structure has been deposited at the Protein Model Archive (10.5452/ma-w81qa). Our model is consistent with previous studies on IFITM3 topology and structure^10,33,34^. It has a type II TM topology of IFITM3 consistent with Bailey et al.^33^. It is consistent with secondary structure predictions from Ling et al. and Chesarino et al.^10,34^, showing that this region of IFITM3 has three alpha-helices. Finally, the orientation of the first alpha helix, which is an amphipathic helix, is also consistent with these studies, with the residues N64 and T65 in the hydrophilic face of the helix being cytosolic exposed in our model. The IFITM3 model was also compared with AlphaFold model (AF-Q01628-F1; RMSD of 3.5 A to our model)^35^; it was observed that the AlphaFold model does not comply with the topology of IFITM3 as it is predicted as a type II transmembrane protein (see also “Discussion” and Supplementary Fig. S1A). The initial model was found to have one residue (Gln97) in disallowed region of Ramachandran map as assessed by PROCHECK. The overall model quality was observed to be satisfactory as assessed by ProSA-web (Supplementary Fig. S1B). This initial model was prepared using protein preparation wizard of Schrodinger which involves H-bond optimization and constrained minimization. The minimized structure was utilized for MD simulation. The modelled structure was simulated in membrane-aqueous environment. The RMSD was calculated with respect to frame 0 and the RMSD was found to stabilize after 20th ns (Fig. 2A). The residue-wise fluctuation was also recorded using RMSF analysis and it was observed that the terminal residues are more flexible (Fig. 2B). Further, radius of gyration was calculated to assess the change in compactness of the model and it was observed that the ROG at the end of the simulation is less than at the beginning indicating that the compactness increases over simulation time (Fig. 2C).

The RMSD between initial model (Fig. 2D) and the structure at the end of simulation (Fig. 2E) is 5 Å. The structure at the end of the simulation was considered for ligand binding site prediction and virtual screening (Fig. 2E).

### Virtual screening using FDA dataset revealed potent binders of IFITM3

**Figure 3:**
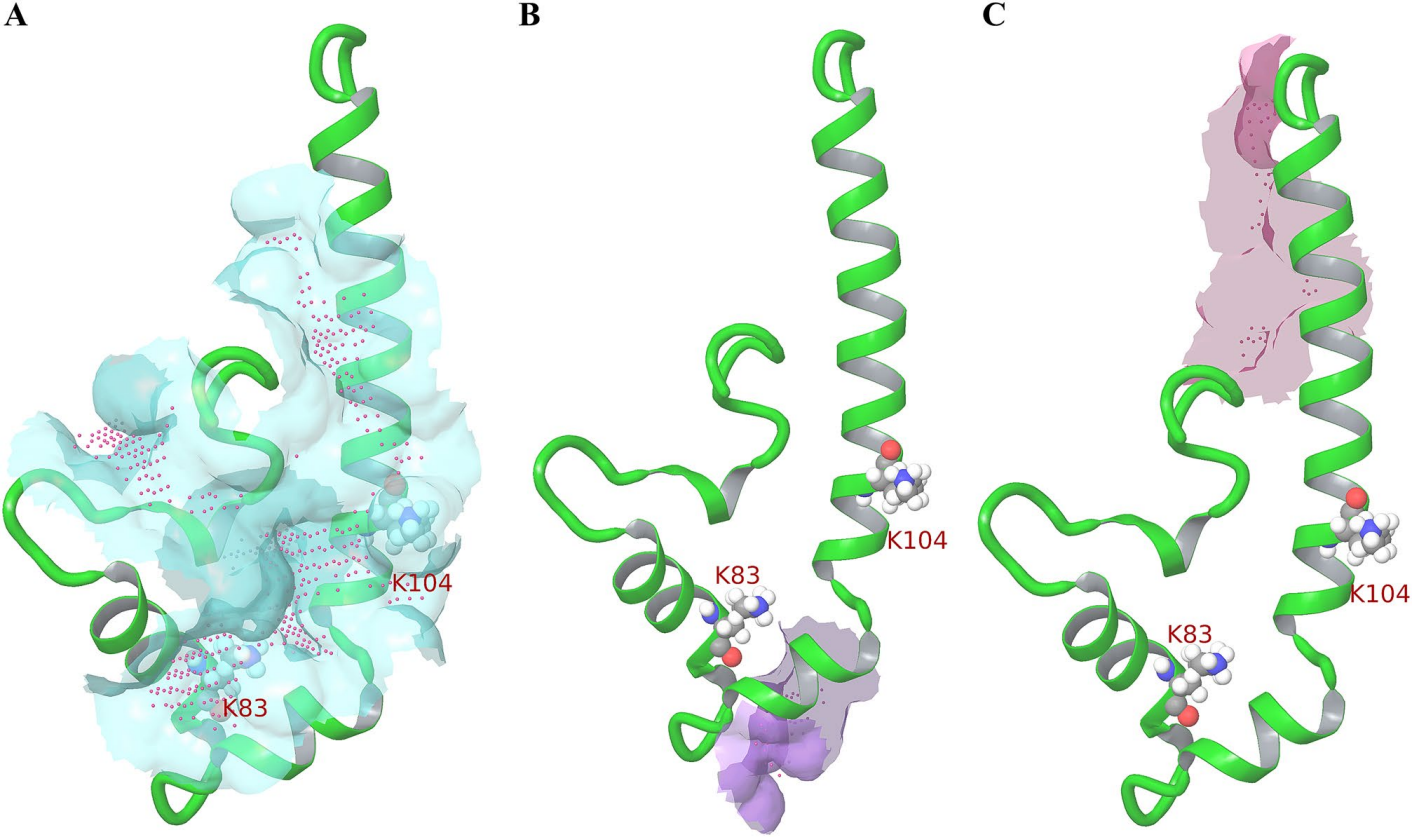
Sites predicted by SiteMap (**A**) Site 1 in cyan (**B**) Site 2 in purple (**C**) Site 3 in pink. Important lysine residues have been labelled. IFITM3 has been shown as green ribbon. Lys83 and Lys104 have been represented as ball and stick. Magenta dots indicate site points.

The possible ligand binding sites were predicted for the IFITM3 model using SiteMap tool and the top site (Site 1) has satisfactory SiteScore (0.899) and Dscore (1.033)^36^. This site contains functionally relevant residues like Lys83 and Lys104 and hence it was considered for docking experiment (Fig. 3A). Two more sites were predicted with poor SiteScore and Dscore. Site 2 has 0.660 SiteScore and 0.724 Dscore (Fig. 3B) while Site 3 has 0.615 SiteScore and 0.690 Dscore (Fig. 3C). The receptor grid was generated using Site 1 and the prepared library of 2310 FDA ligands were docked sequentially to this site. The HTVS docking retained 1888 ligands and resulted in “Kaolin” as top hit with docking score of − 7.845. All of HTVS hits were subjected to SP docking and “Nafarelin” with docking score of − 9.135 was found to be the top hit compound. The top 1000 ligands from SP results were subjected to XP docking and the best ligand is “Amikacin” having docking score of − 10.464 kcal/mol. Kaolin was also found to be the 3rd best ligand (− 9.826 kcal/mol). All of the XP ligands were re-ranked based on the binding energy calculations from MM-GBSA where limited protein flexibility was considered along with implicit membrane. “Fidaxomicin” was the top hit with ΔG of − 88.8 kcal/mol (ranked 10th as per XP docking score). The top hits were examined for their interactions and their clashes with membrane (Supplementary Fig. S2).

**Figure 4:**
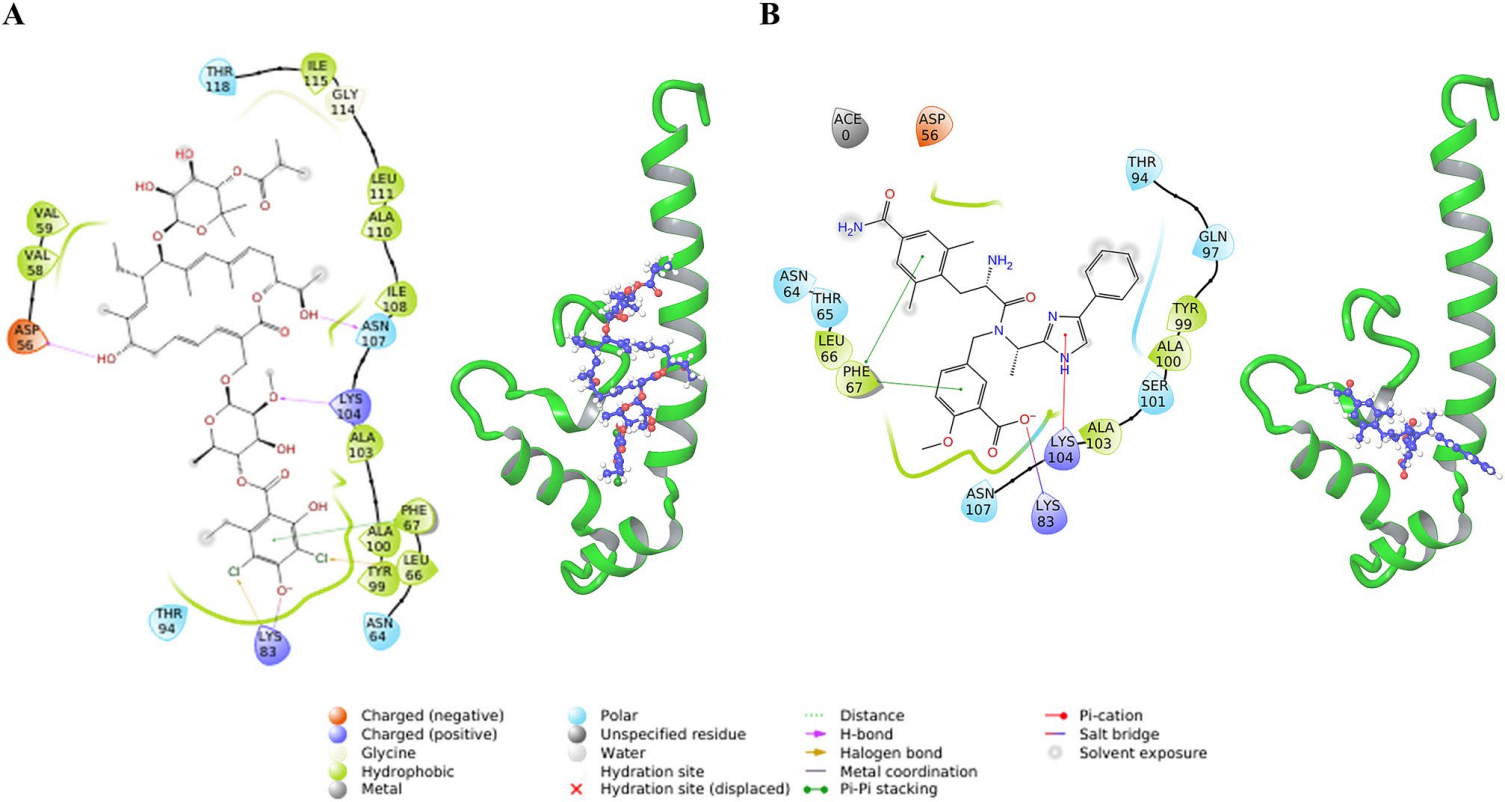
Interactions of ligands in XP-pose with IFITM3 (**A**) Fidaxomicin (**B**) Eluxadoline.

The complex with Fidaxomicin shows H-bond and salt-bridge interactions with Lys104 and Lys83 respectively. It is involved in H-bond interactions with Asn107 and Asp56 as well. Phe67 forms Pi–Pi stacking interaction with fidaxomicin. Halogen bond interactions are also formed between fidaxomicin and residues Lys83 and Tyr99 (Fig. 4A). Further, the membrane information was overlayed and fidaxomicin was found to overlap with the POPC membrane. Among the top ten hits, Eluxadoline was found to interact with Lys83 and Lys104 while not overlapping with the membrane (Fig. 4B). Eluxadoline also forms Pi–Pi stacking interactions with Phe67. A similar analysis of docked complexes was done for the top thirty hits based on ΔG and the ligands without overlap with membrane include Naloxegol, Valrubicin, Thiethylperazine, Montelukast, Sacubitril and Ertugliflozin. There are antifungal compounds like Itraconazole and Isavuconazonium among the top ten hits. Antivirals like Ledipasvir, Atazanavir, Telaprevir and Indinavir were also among the top fifty hits. The known functions and the docking score of these ligands have been summarized in Supplementary Table S1.

### MD simulation revealed stable IFITM3 interacting FDA ligands

**Figure 5:**
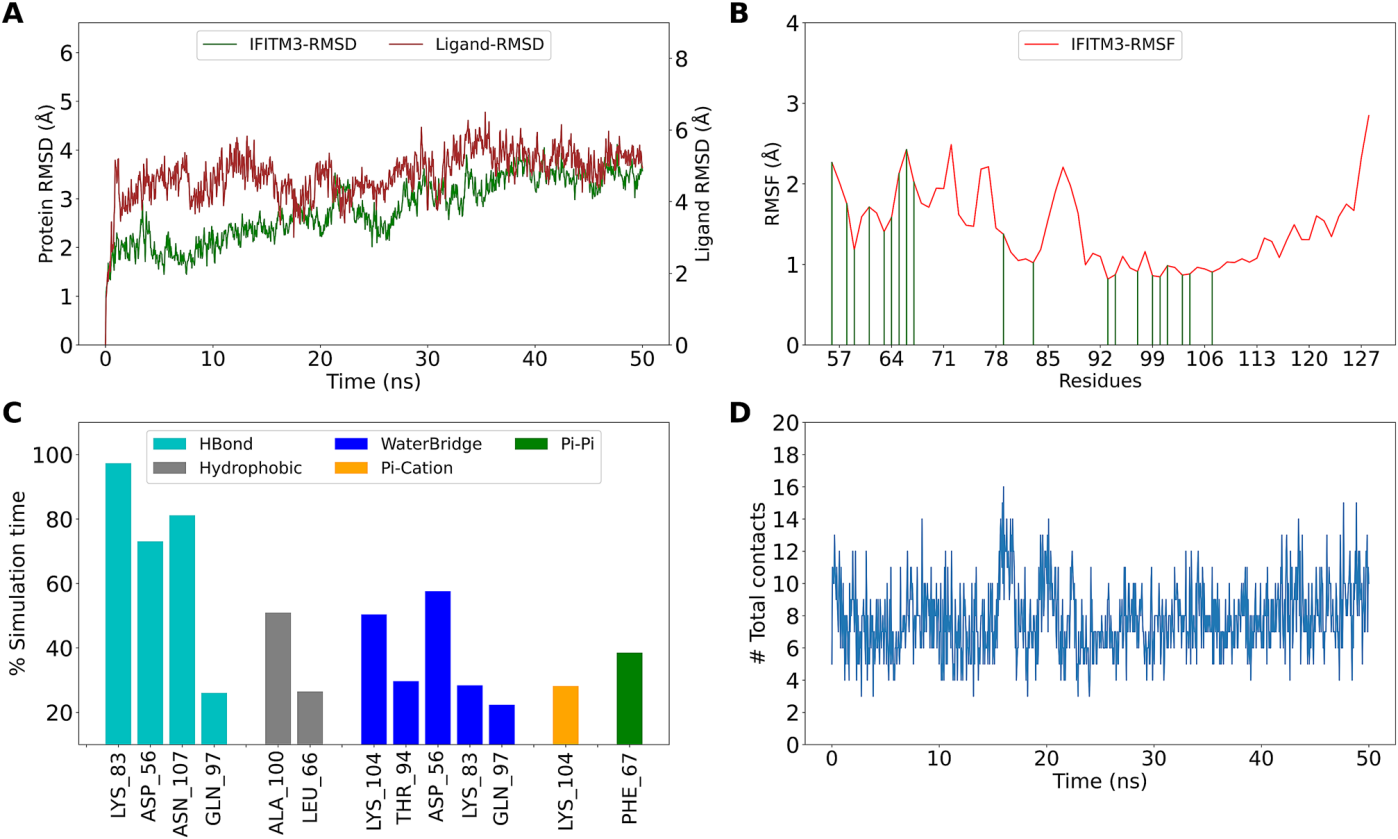
MD simulation of IFITM3-Eluxadoline complex (**A**) RMSD of IFITM3 and Eluxadoline fit on IFITM3 (Ligand-RMSD) (**B**) RMSF of IFITM3. Green lines indicate interactions with Eluxadoline (**C**) Interactions between IFITM3 and Eluxadoline as percentage of simulation time. Interactions that persist for more than 20% of simulation time have been shown (**D**) Total contacts (includes all interactions) between Eluxadoline and IFITM3.

Next, MD simulations were used to assess the stability of and interactions in the protein–ligand complex. The XP pose of top FDA ligands (not having membrane overlap) were subjected to MD simulation and their stability was assessed. Initially, a 50 ns MD simulation was performed for ligands within the top thirty hits (Eluxadoline, Naloxegol, Valrubicin, Sacubitril, Ertugliflozin, Thiethylperazine, Montelukast) as per the ΔG score. The complex of IFITM3- Eluxadoline remains stable as assessed by RMSD. The ligand RMSD was calculated for the ligand after aligning the protein–ligand complex on the protein backbone (Fig. 5A). The residue fluctuations of IFITM3 were observed throughout the simulation time and residues that interacts with the Eluxadoline are shown (Fig. 5B). The non-covalent interactions between IFITM3 and Eluxadoline were calculated and Lys83 was found to interact for more than 90% of simulation time while Lys104 interacts via H-bonds and Pi–cation interactions that persist for less than 40% of simulation time. Other major interacting residues are Asn107, Asp56, Ala100 and Phe67. A few residues are involved in interactions via water-bridge and the prominent residues are Lys104 and Asp56 (Fig. 5C). The interactions between IFITM3 and Eluxadoline were maintained throughout the simulation time, as observed by total contact at each time point (Fig. 5D). Among others, Valrubicin, Sacubitril and Montelukast were found to be stable and retain interactions with Lys83 and Lys104 (Supplementary Figs. S3–S5).

Naloxegol does not form a stable complex with IFITM3 (Supplementary Fig. S6). Ertugliflozin does not interact with Lys83 or Lys104 for more than 20% of simulation time (Supplementary Fig. S7). Thiethylperazine remains stable but does not interact with Lys83 or Lys104. It forms Pi–cation and Pi–Pi interactions with Phe67 but loses contact with IFITM3 at multiple time points across simulation (Supplementary Fig. S8). Further, few more ligands from top 100 hits were selected for MD simulation (20 ns) and it was observed that Cephaloglycin forms a stable complex with IFITM3 and interacts with Lys83 through H-bond and with Lys104 through Pi–cation interaction (Supplementary Fig. S9). Other ligands with stable interaction with IFITM3 include Aprepitant (Supplementary Fig. S10), Fusidic acid (Supplementary Fig. S11), Mitoxantrone (Supplementary Fig. S12), Tafenoquine (Supplementary Fig. S13), Riboflavin (Supplementary Fig. S14) and Roflumilast (Supplementary Fig. S15). A few ligands were found to be unstable upon simulation and these include Atazanavir (Supplementary Fig. S16), Hesperidin (Supplementary Fig. S17), Elbasvir (Supplementary Fig. S18) and Amikacin (Supplementary Fig. S19). Based on these analysis, the promising FDA compounds that can bind to IFITM3 and perturb its function include Eluxadoline, Valrubicin, Sacubitril, Montelukast, Cephaloglycin and Fusidic acid.

### Virtual screening using natural compounds revealed more potent binders of IFITM3

**Figure 6:**
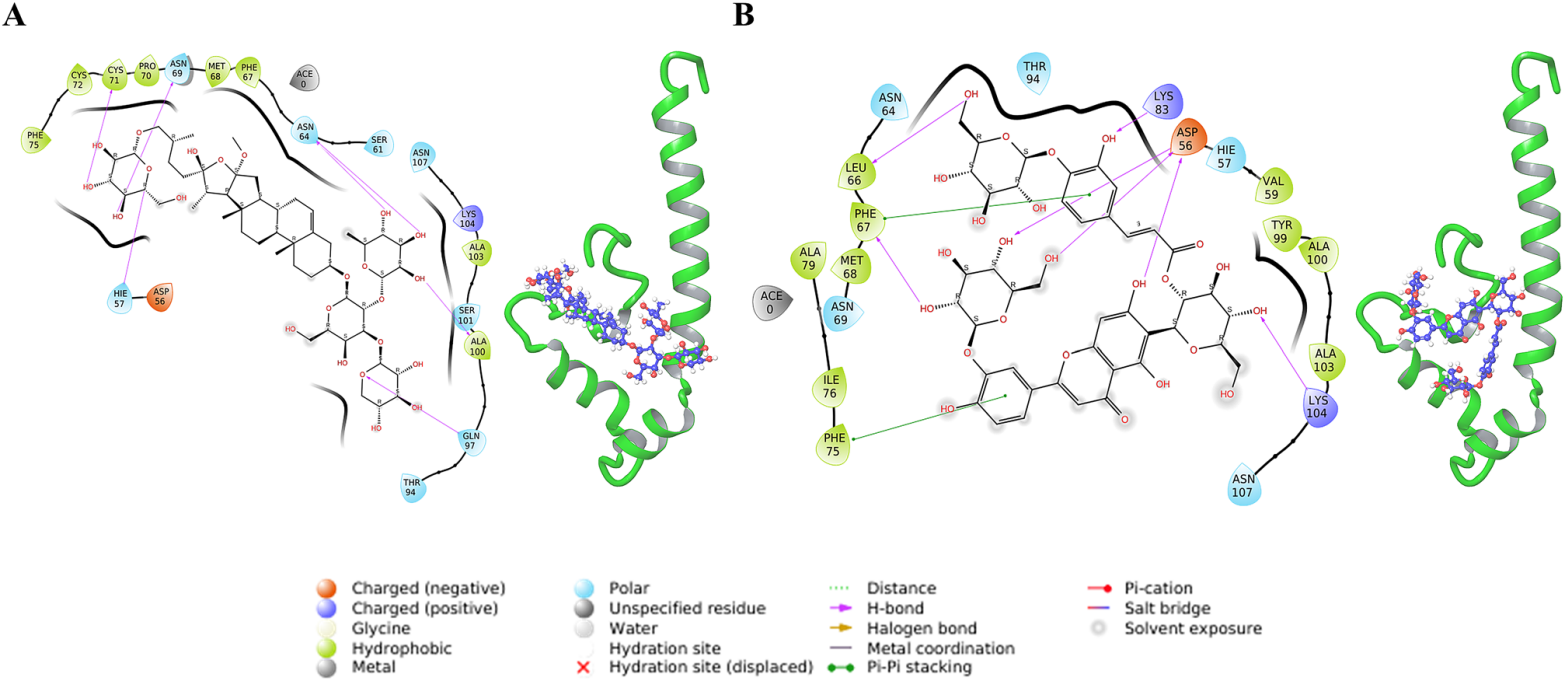
Interactions of natural ligands in XP-pose with IFITM3 (**A**) SN00224572 (**B**) SN00249458.

The same binding site of IFITM3 was utilized to screen library of Natural products from Super Natural II database. A total of 3,22,927 compounds were screened. The initial HTVS retained 2,44,618 compounds with best docking score of − 8.752 kcal/mol. Top 24,500 compounds of HTVS were used for SP docking which retained 24,376 compounds and the best docking score was found to be − 10.285 kcal/mol. Top 2500 compounds from SP docking results were subjected to XP docking which resulted in best docking score of − 13.781 kcal/mol. All of the XP poses were re-ranked by calculating ΔG using MMGBSA. The best compound is “SN00224572” with a ΔG of − 100.79 kcal/mol, which is better than the best compound from FDA dataset. The compound “SN00224572” forms H-bond interactions with His57, Asn64, Asn69, Cys71, Gln97 and Ala100 (Fig. 6A).

The top compound which interacts with both Lys83 and Lys104 is SN00249458 (3rd Rank as per ΔG score). SN00249458 forms H-bond with Asp56, Leu66 and Phe67 apart from Lys83 and Lys104. Phe67 and Phe75 form Pi–Pi stacking interactions with SN00249458 (Fig. 6B). Interactions in IFITM3–ligand complex and ligand overlap with membrane were analysed for other top hits (Supplementary Fig. S20).

### IFITM3 makes stable complex with ligands from Super Natural II database

**Figure 7:**
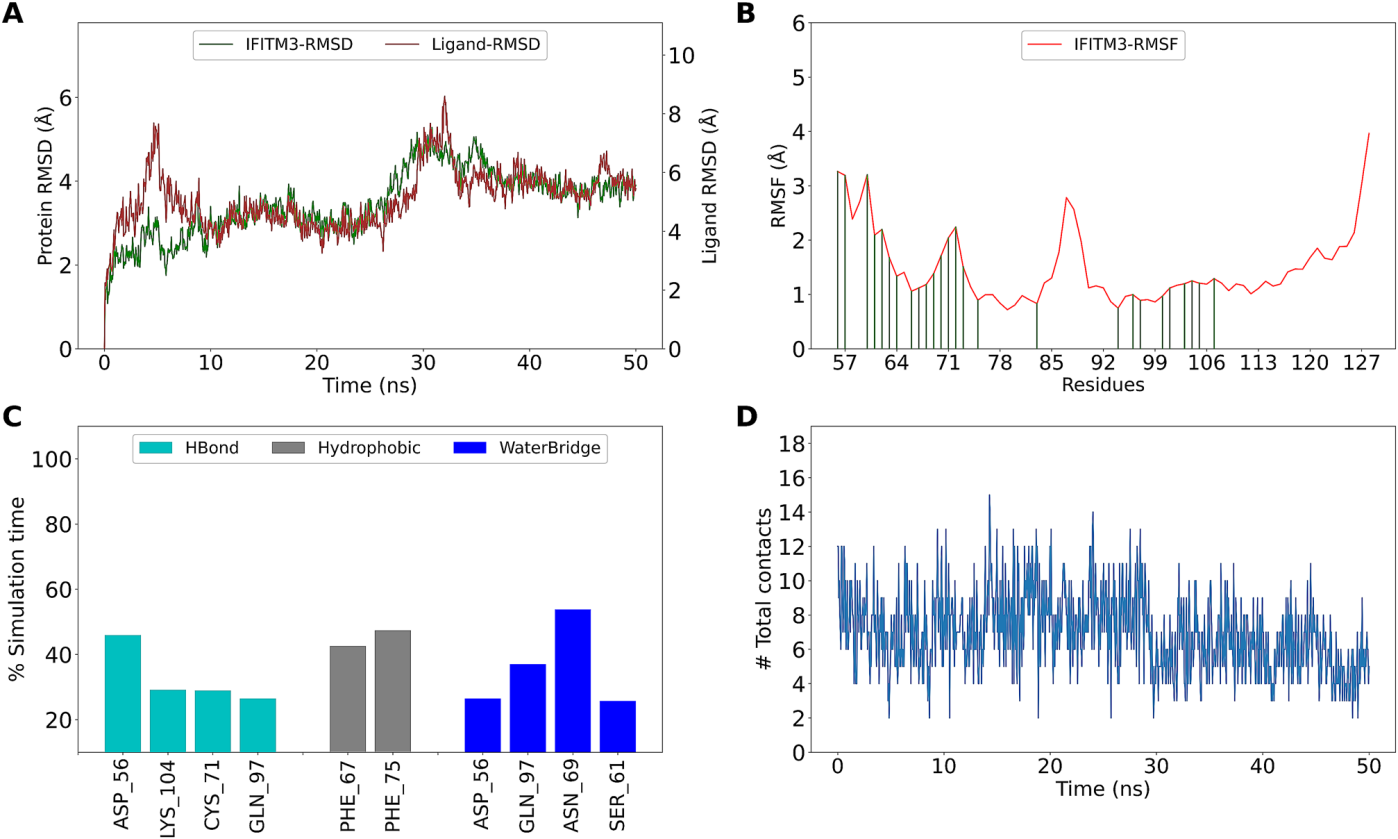
MD simulation of IFITM3-SN00224572 complex (**A**) RMSD of IFITM3 and SN00224572 “fit on protein” (**B**) RMSF of IFITM3. Green lines indicate interactions with SN00224572 (**C**) Interactions between IFITM3 and SN00224572 as percentage of simulation time (**D**) Total contacts (includes all interactions) between SN00224572 and IFITM3.

The complex of top hits from SNDB with IFITM3 were subjected to MD simulation to assess the interaction stability. SN00224572 forms stable complex with IFITM3 (Fig. 7A). The residue fluctuations of IFITM3 were observed to be less in the region 92–110 and these residues make contact with SN00224572 (Fig. 7B). Asp56, Cys71, Gln97 and Lys104 form H-bond interactions with SN00224572 for more than 20% of simulation time. Lys83 forms transient ionic interactions and persistent water-bridge interaction with SN00224572 (Fig. 7C). There are at least 2 contacts maintained throughout the simulation time between IFITM3 and SN00224572 (Fig. 7D). The IFITM3- SN00224572 complex can be considered stable owing to these interactions.

Similar simulation and analysis were done for other top hits. Compound SN00249458 was found to have more contacts with IFITM3 than SN00224572. SN00249458 interacts mainly with Asp56, Asn64, Asn69, Asp86 via H-bonds and with Phe67 via Pi–Pi interaction (Supplementary Fig. S21). Among the top five hits, SN00342783 and SN00328659 (Saponin PK) were found to be not stable complexes while SN00226205 remains stable (Supplementary Figs. S22–S24). SN00244021 has a very good docking score (− 13.621) and ΔG score (− 93.78). MD simulation of SN00244021 revealed that it forms stable complex with IFITM3 and interacts mainly with Asn69 and Asp56 for more than 80% simulation time. It also interacts with Lys83 through water-bridge interaction (Supplementary Fig. S25). The top compound without any severe clash with the membrane is SN00164639 (13th rank as per ΔG score). MD simulation of SN00164639 revealed multiple interactions with IFITM3 but the RMSD was not stabilized (Supplementary Fig. S26).

Of the top hits, there are hits with common names like SN00265483 (Periseoside D), SN00274576 (Pharbitic acid D), SN00287660 (Pseudoceratinazole A), SN00379347 (Parishin A), SN00267584 (MIMENGOSIDE F), SN00306006 (Hosenkoside B), SN00389392 (Landomycin U), SN00359919 (Crambescidin 826), SN00374092 (Anguivioside B), SN00274346 (Disporoside D), SN00226238 (Broussonetine Q), SN00242218 (Nazumamide A), SN00226669 (Kukoamine A), SN00304961 (Rhodilunancin A) and SN00318481 (Caryocaroside II-12). The docking scores and ΔG of these have been summarized in Supplementary Table S2. MD simulations were performed for Periseoside D, Pharbitic acid D, Pseudoceratinazole A, Parishin A, Hosenkoside B, Anguivioside B, Disporoside D, Broussonetine Q, Nazumamide A, Kukoamine A, Rhodilunancin A and Caryocaroside II-12. Among these compounds, Pharbitic acid D, Pseudoceratinazole A, Parishin A, Anguivioside B, Broussonetine Q, Nazumamide A and Caryocaroside II-12 form stable complex with IFITM3 (Supplementary Figs. S27–S38). Pharbitic acid D interacts via H-bond with Lys83 for more than 70% simulation time and forms water-bridge interaction with Lys104 (Supplementary Fig. S28). Pseudoceratinazole A forms a H-bond with Lys104 that persists for more than 20% simulation time and it forms Pi–cation interaction with Phe67 (Supplementary Fig. S29). Parishin A was found to interact with both Lys83 and Lys104 for more than 60% of simulation time. Phe67 also interacts majorly through hydrophobic interaction for more than 60% of simulation time. Parishin A maintains contact at each time point throughout the simulation time (Supplementary Fig. S30). Anguivioside B does not form persistent interaction with Lys83 or Lys104 (Supplementary Fig. S32). Broussonetine Q forms water-bridge interaction with Lys104 (Supplementary Fig. S34). Nazumamide A also forms water-bridge interaction with Lys104 but it interacts with Asp56 and Phe67 for more than 40% simulation time via ionic and Pi–cation interactions respectively (Supplementary Fig. S35). Caryocaroside II-12 interacts with Lys104 through H-bond that persists for more than 60% simulation time (Supplementary Fig. S38).

Further, MD simulation was also performed for SN00239590, SN00323932, SN00286991, SN00280809 and SN00029983 (Supplementary Table S2). It was observed that SN00239590 interacts with both Lys83 and Lys104 via ionic and H-bond interaction but the complex is not stable as assessed by the RMSD values (Supplementary Fig. S39). SN00323932 loses contact with IFITM3 during simulation (Supplementary Fig. S40). SN00286991 remains very stable throughout simulation and interacts with Lys104 through Pi–cation interaction (Supplementary Fig. S41). SN00280809 also forms stable complex and interacts with both Lys83 and Lys104 (Supplementary Fig. S42). SN00029983 does not form stable complex with IFITM3 (Supplementary Fig. S43). In summary, SN00224572, SN00249458, SN00226205, SN00244021, SN00286991, SN00280809, Pharbitic acid D, Pseudoceratinazole A, Parishin A, Anguivioside B, Broussonetine Q, Nazumamide A and Caryocaroside II-12 are the natural compounds which form stable interactions with IFITM3.

## Discussion

IFITM3 is involved in multiple pathologies including cancer and Alzheimer^27,28^. It has also been implicated in inhibition of various viral entry. For some pathologies, inhibition of IFITM3 will be beneficial (cancer conditions), whereas for other conditions, such as inhibition of viral entry, the activation of IFITM3 can be useful. Several post translational modifications regulate the activity of IFITM3. It gets ubiquitinated at Lys83 and Lys104 for degradation. Therefore, the avoidance of degradation can potentially increase its lifetime. Computational approaches are an efficient way to find potential IFITM3 binders, both inhibitors of IFITM3 function and inhibitors of its interaction with other proteins. Towards this goal, the structure of IFITM3^56–128^ was modelled.

Three different membrane topologies, an intramembrane topology, a type III transmembrane (TM) topology, and a type II transmembrane topology, for IFITM3 have earlier been proposed for IFITM3^11^. However, recent studies have shown that IFITM3 is a type II transmembrane protein with a single TM helix^10,33,34^. Our model structure of IFITM3^56–128^ (Protein Model Archive 10.5452/ma-w81qa) is consistent with these recent studies on IFITM3 topology and structure, and has a type II TM topology, with three alpha helices, including an amphipathic helix. The structure predicted by Alphafold (model AF-Q01628-F1; RMSD of 3.5 A to our model)^35^ however has a topology of type III transmembrane proteins with two transmembrane helices^7,10,34^. Hence it was not used in our investigation. The modelled structure was simulated in the membrane-aqueous environment to get the thermodynamically stable conformation of IFITM3. The MD parameters (like RMSD, RMSF and ROG) were checked to assess the model quality. The final structure at the end of the simulation was considered for docking studies. The structure at the end of the simulation was utilized to predict drug-binding sites. The top binding site was found to have residues important for post translational modifications, like Lys83, Lys104 which get ubiquitinated and Cys71, Cys72 which get Palmitoylated. Lys104 is also a part of CARC motif which is important for cholesterol binding^14,15^. This site was also found to have good druggable score. Ligand interactions with PTM residues can affect the interactions of IFITM3 with other proteins.

First, virtual screening was carried out using the FDA dataset against this site. In the FDA dataset, all of the top ten ligands based on ΔG values were found to be interacting with at least one critical lysine residue. Further, simulation studies revealed the stability of interaction of these compounds indicting their role as potential IFITM3 binders. The most probable IFITM3 binders based on the simulation analysis include Eluxadoline, Montelukast, Valrubicin and Sacubitril. Eluxadoline is a mixed opioid receptor agonist and it is used to treat irritable bowel syndrome. Montelukast is used for Asthma therapy and is an antagonist of leukotriene receptor. Sacubitril is used in case of chronic heart failure and it is an inhibitor of neprilysin. Valrubicin is used for BCG-resistant bladder and is semisynthetic analog of anthracycline drug doxorubicin. Other than these top hits, there are few antifungal compounds like Itraconazole (used for treatment of pulmonary and extrapulmonary blastomycosis), Isavuconazonium and Amphotericin B. Amphotericin B is known to counteract the antiviral activity of IFITM3 by preventing an increase in the membrane order^13,37^. MD simulations showed that Amphotericin B forms a stable interaction with IFITM3 (Supplementary Fig. S44). Antiviral drugs like Ledipasvir (against hepatitis C virus), Atazanavir (against HIV), Telaprevir (against chronic Hepatitis C Virus) and Indinavir (against HIV) were also retained among hits. Such compounds can be validated experimentally for their role in pathologies involving IFITM3.

Further, more than 0.3 million natural compounds were screened against the same drug binding site of the IFITM3. The final set of compounds after docking were re-ranked based on ΔG score and the top hit (SN00224572) showed a better binding energy (− 100.79 kcal/mol) than the top hit from FDA dataset. MD simulations similar to the previous case revealed the interaction stability of SN00224572. SN00249458 was also found to interact strongly with IFITM3, whereas a few other promising top hits from the docking approach were found to be unstable upon simulation. Parishin A was found to be stable upon simulation and also interacts with key lysine residues. Parishin A has been demonstrated to have protective effects in case of brain disorders. Our results suggest that the neuroprotective effect of Parishin A could possibly be through binding to IFITM3 and thereby abrogating its activity.

Some of the top hit compounds from the FDA dataset, such as Naloxegol, Valrubicin, Montelukast, Sacubitril, Aprepitant, Fusidic acid, Hesperidin, Roflumilast, Riboflavin, Cephaloglycin and the Super Natural II dataset, such as SN00226205, SN00244021, SN00164639, SN00239590, SN00323932, SN00286991, SN00280809, SN00274576, SN00379347, SN00029983, SN00374092, SN00274346, SN00226238, SN00226669 interact with the residues of the GxxxG motif which is known to be important for IFITM3 oligomerization (Supplementary Figs. S2 and S20). Therefore, the interaction of IFITM3 with these compounds might impact its oligomerization^13^. The top hits from both FDA and Super Natural II datasets interact with the Lys104 which is part of the cholesterol binding site in IFITM3 (Figs. 4, 5, 6, 7)^15^. Top hits from the FDA dataset (Supplementary Fig. S2) and the top hits from the Super Natural II dataset (Supplementary Fig. S20) also interact with Phe67, which is also involved in cholesterol binding^14^. Therefore, these compounds may alter the cholesterol binding activity of IFITM3, and hence its function.

The current study has the following limitations. The lack of an experimental structure of IFITM3 to do virtual screening is a limitation. Secondly, the predicted modulators need to be experimentally validated. In-vitro binding experiments on recombinant IFITM3 could provide a starting point for establishing the binding of the predicted compounds, followed by detailed functional assays in cell lines. Third, our approach does not distinguish whether the bound ligand will enhance the activity of IFITM3 (desired outcome to inhibit viral entry) or abrogate the activity of IFITM3 (desired in case of multiple cancers). In case of inhibitors of IFITM3 activity, the susceptibility to viral infection can increase. Therefore, combining these inhibitors with anti-viral drugs may be necessary. Another strategy is to consider top hits which are known antiviral drugs. Both the above strategies might be combined to compensate for the lack of anti-viral activity during IFITM3 inhibition. Experimental validation of predicted good binders will help establish how the lack of anti-viral activity of IFITM3 can be compensated. Finally, a few top hit compounds as per binding energy overlapped with the membrane and therefore it was not feasible to perform MD simulations on these.

## Conclusion

IFITM3 has been established as a therapeutic target against cancer and it has been shown to affect multiple viral infections including SARS-CoV2. Its function is regulated by multiple post-translational modifications. In this study we have modelled the structure of IFITM3 and predicted a potential drug-binding site consisting of key residues involved in post translational modifications. This binding site was assessed for the binding of FDA approved drugs and natural products from Super Natural II database. We found that known drugs like Eluxadoline, Valrubicin, Sacubitril, Montelukast, Cephaloglycin and Fusidic acid have good binding affinity against IFITM3. Among natural products, it was observed that major IFITM3-interacting ligands include SN00224572, SN00249458, SN00226205, SN00244021, SN00286991, SN00280809, Pharbitic acid D, Pseudoceratinazole A, Parishin A, Anguivioside B, Broussonetine Q, Nazumamide A and Caryocaroside II-12. Experimental validation can reveal the effect of these compounds on the activity of IFITM3 and pathologies associated with IFITM3.

## Material and methods

### Modelling of IFITM3 structure

We modelled residues 56–128 of human IFITM3, comprising of the conserved CD225 region and a transmembrane domain, i.e., all of IFITM3 except its N and C-terminal cytosolic domains. The CD225 region in turn comprises of a conserved intra-membrane domain and a conserved intracellular loop^7^. The modelled region consists of two α-helices and a transmembrane α-helix, according to two independent experimental studies^10,34^.

The human IFITM3 protein sequence was obtained from Uniprot (Q01628). The IFITM3 56–128 structure was modelled by homology modelling using Modeller^38^. Two templates were used. First, a homology model of human IFITM3 was taken from Modbase (EN ENSP00000382707.4). The template used for Modbase is PDB 1dkq with 31% identity to IFITM3 sequence. Low-confidence regions were truncated from this model, resulting in a model for IFITM3 56–107^39^. Next, a template for the transmembrane region that was not covered by the Modbase model was used (PDB 3HD7)^40^. This template was detected by running HHPred on human IFITM3 sequence^41^. The local sequence identity between 3HD7 and IFITM3 transmembrane region is 60%.

Our model is consistent with previous studies on IFITM3 membrane structure and topology^10,11,34^. Bailey et al. demonstrated that IFITM3 is a type II transmembrane protein with its N-terminus in the cytosol and C-terminus in the extra-cellular space, using surface staining, fluorescence microscopy, and lysosomal degradation assays^33^. Ling et al. showed that IFITM3 possesses two intra-membrane helices, residues 62–67 and 76–85, and a TM helix, residues 96–131^10^. Largely in agreement with Ling et al. and Chesarino et al. showed that IFITM3 has three alpha-helical regions, with two distinct alpha-helices in residues 59–68 and 73–89, separated by an unstructured region, and a TM helix (93–131) using a combination of bioinformatics tools^34^. Our model is consistent with the above three studies, i.e. the model has a type II TM topology of IFITM3 and is consistent with the secondary structure predictions from Ling et al. and Chesarino et al.^10,34^. Finally, the orientation of the first alpha helix, which is an amphipathic helix, is also consistent with that of Ling et al. and Chesarino et al.^10,34^. The residues N64 and T65 in the hydrophilic face of the helix are cytosolic exposed in our model.

### Molecular dynamics simulation

The modelled IFITM3 structure was prepared using protein preparation wizard of Schrodinger and subjected to MD simulation (50 ns) in the presence of membrane using Desmond^42,43^. POPC membrane and TIP4P water model was used in the System Builder panel of Schrodinger. The membrane placement was manually adjusted followed by neutralization of the system and NaCl was added at 0.15 M concentration. The output of System builder was subjected to MD simulation using Desmond. The default relaxation protocol (includes minimization and short MD runs) was used before production run of 50 ns in NPT ensemble at 300 K using OPLS3e force field^44^. For protein–ligand complex, the system was prepared using the system builder and the membrane information was obtained from the IFITM3 structure considered for the docking. MD simulation was not performed in cases where direct membrane placement resulted in severe clashes (overlap) with docked ligand.

### Molecular docking

The IFITM3 structure at the end of 50 ns simulation was considered for molecular docking. Binding sites were predicted using SiteMap tool^36,45^. Site 1 was found to have good druggability score and involved important residues like Lys83 and Lys104 that undergo post-translational modification. Ligands from FDA approved drugs^46^ or Super Natural II database^47^ were prepared using LigPrep tool. The molecular docking was performed using glide^48–50^. Initially HTVS (High-throughput virtual screening) method was employed, followed by SP (Standard precision) docking. Reward intramolecular H-bond and enhance planarity of conjugated pi groups were considered for docking. Further, aromatic h-donors at partial charge cut off of 0.1 and halogens as acceptors were considered. Strain correction terms were applied. XP (Extra precision) docking was carried out for top hits from SP docking with similar parameters as SP. The XP poses were subjected to binding energy calculation using MM-GBSA tool of Prime module of Schrodinger. For this calculation, IFITM3 residues within 4 Å of ligand were considered flexible and harmonic constraint (1.0 kcal mol^−1^ Å^−2^) was used on these flexible residues. The ligands were re-ranked based on ΔG of binding. The best ligands from each dataset based on the binding energy were subjected to MD simulation to assess the interaction stability.

### MD simulation result analysis

The simulation results of nascent protein or protein–ligand complex were analysed using simulation interaction diagram (SID), simulation quality analysis and simulation event analysis of Schrodinger. The root mean squared deviation (RMSD) and root mean squared fluctuation (RMSF) were calculated using the first frame as reference structure. “Ligand RMSD” indicates the RMSD of ligand after superposition of protein–ligand complex to the reference structure followed by RMSD calculation of ligand. Higher values of “Ligand RMSD” compared to protein RMSD suggests diffusion of ligand from the initial binding site.

## Supplementary Information

Supplementary Information.

## Data Availability

The IFITM3 model has been deposited to Model Archive at 10.5452/ma-w81qa. Modeled IFITM3 structure and MD simulation trajectories of IFITM3 and IFITM3-ligand complexes are deposited to Zenodo at the 10.5281/zenodo.6787966.
