# Supplementary material for: Identification of potential modulators of IFITM3 by in-silico modeling and virtual screening
Source: Sci Rep. 2022 Sep 24;12:15952. doi: 10.1038/s41598-022-20259-8 (PMC9509314; doi:10.1038/s41598-022-20259-8)
Supplement: Supplementary file 1 — Supplementary Information. [file 41598_2022_20259_MOESM1_ESM.docx]

**Supplementary Information for**

**Identification of potential modulators of IFITM3 by *in-silico* modeling and virtual screening**

Vikas Tiwari and Shruthi Viswanath*

National Centre for Biological Sciences, Tata Institute of Fundamental Research, Bangalore 560065, India

*Corresponding author. Email: shruthiv@ncbs.res.in

Short title: Drug-discovery for IFITM3

**Supplementary Tables**

**Table S1:** Top hits from FDA dataset

| Rank as per ΔG (XP rank) | Compound | ΔG | Docking score (XP) | Activity |
| --- | --- | --- | --- | --- |
| 1 (10) | Fidaxomicin | -88.8 | -8.735 | Novel macrolide antibiotic used in the treatment of diarrhea |
| 7 (122) | Eluxadoline | -74.57 | -5.975 | Mixed mu-opioid receptor agonist, kappa-opioid receptor agonist, and a-delta opioid receptor antagonist indicated for use in diarrhea-predominant irritable bowel syndrome |
| 3 (498) | Itraconazole | -80.78 | -4.066 | Antifungal. A highly selective inhibitor of fungal cytochrome P-450 sterol C-14 α-demethylation via the inhibition of the enzyme cytochrome P450 14α-demethylase |
| 10 (998) | Isavuconazonium | -73.53 | 0.717 | Antifungal. Used for the treatment of invasive aspergillosis and mucormycosis. |
| 15 (72) | Naloxegol | -71.03 | -6.525 | Peripherally-selective opioid antagonist |
| 19 (100) | Valrubicin | -67.38 | -6.193 | Semisynthetic analog of the doxorubicin. Treatment of the bladder cancer |
| 21 (321) | Thiethylperazine | -66.18 | -4.746 | A dopamine antagonist that is particularly useful in treating the nausea and vomiting associated with anaesthesia |
| 22 (444) | Montelukast | -65.38 | -4.328 | Leukotriene receptor antagonist. used typically in addition to or complementary with the use of inhaled corticosteroids or other agents in asthma step therapy |
| 24 (16) | Sacubitril | -65.14 | -7.857 | A prodrug neprilysin inhibitor used in combination with valsartan to reduce the risk of cardiovascular events in patients with chronic heart failure (NYHA Class II-IV) and reduced ejection fraction |
| 27 (225) | Ertugliflozin | -64.74 | -5.163 | Potent and selective inhibitors of the sodium-dependent glucose cotransporters (SGLT), specifically the type 2 which is responsible for about 90% of the glucose reabsorption from glomerulus |
| 14 (429) | Ledipasvir | -71.17 | -4.316 | Antiviral. Inhibits NS5A of HCV |
| 41 (221) | Atazanavir | -62.35 | -5.064 | Antiviral. HIV-1 protease inhibitor |
| 45 (530) | Telaprevir | -61.65 | -3.922 | Antiviral. Inhibitor of NS3/4a protease of HCV |
| 47 (333) | Aprepitant | -61.44 | -4.620 | Antagonist of P/neurokinin 1 (NK1) receptor. Antiemetic agent. |
| 48 (508) | Indinavir | -61.41 | -4.019 | Antiviral. Inhibits HIV protease |
| 55 (52) | Fusidic acid | -60.01 | -6.748 | Antibiotic. Interferes with bacterial protein synthesis |
| 68 (55) | Hesperidin | -57.08 | -6.668 | Bioflavonoid. Effective in blood vessel disorders |
| 79 (792) | Elbasvir | -55.65 | -3.109 | Antiviral. Inhibits NS5A of HCV |
| 80 (179) | Mitoxantrone | -55.64 | -5.402 | Used in multiple sclerosis. Inhibits topoisomerase II |
| 84 (92) | Tafenoquine | -55.30 | -6.186 | Treatment for relapsing vivax malaria |
| 85 (388) | Roflumilast | -55.26 | -4.465 | Inhibitor of phosphodiesterase-4 (PDE-4). Used against chronic obstructive pulmonary disease (COPD) exacerbations. |
| 95 (256) | Riboflavin | -54.19 | -4.904 | Vitamin B2 |
| 99 (54) | Cephaloglycin | -53.87 | -6.697 | Antibiotic. Inhibits cell wall synthesis. |
| 716 (1) | Amikacin | -26.25 | -10.464 | Antibiotic. Binds to bacterial 30S ribosomal subunit. |

**Table S2:** Top hits from SNDB dataset

| Rank as per ΔG (XP rank) | Compound | ΔG | Docking score (XP) |
| --- | --- | --- | --- |
| 1 (628) | SN00224572 | -100.79 | -8.312 |
| 2 (16) | SN00342783 | -98.98 | -12.06 |
| 3 (374) | SN00249458 | -98.56 | -9.071 |
| 4 (143) | SN00328659 | -97.01 | -10.167 |
| 5 (278) | SN00226205 | -94.52 | -9.428 |
| 9 (2) | SN00244021 | -93.78 | -13.621 |
| 13 (823) | SN00164639 | -92.38 | -7.884 |
| 27 (675) | SN00239590 | -90.23 | -8.202 |
| 37 (2429) | SN00323932 | -87.60 | -3.242 |
| 41 (1069) | SN00265483 | -86.84 | -7.373 |
| 57 (1572) | SN00286991 | -84.05 | -6.489 |
| 69 (38) | SN00280809 | -83.14 | -11.317 |
| 73 (111) | SN00274576 | -82.75 | -10.498 |
| 115 (1422) | SN00287660 | -80.2 | -6.753 |
| 116 (502) | SN00379347 | -80.15 | -8.639 |
| 167 (450) | SN00306006 | -77.50 | -8.783 |
| 187 (1278) | SN00029983 | -76.61 | -7.026 |
| 247 (312) | SN00374092 | -73.92 | -9.334 |
| 268 (408) | SN00274346 | -72.76 | -8.924 |
| 273 (279) | SN00226238 | -72.36 | -9.427 |
| 279 (642) | SN00242218 | -72.19 | -8.291 |
| 298 (511) | SN00226669 | -71.59 | -8.608 |
| 318 (1853) | SN00304961 | -70.67 | -5.817 |
| 345 (515) | SN00318481 | -69.53 | -8.602 |

**Supplementary Figures**


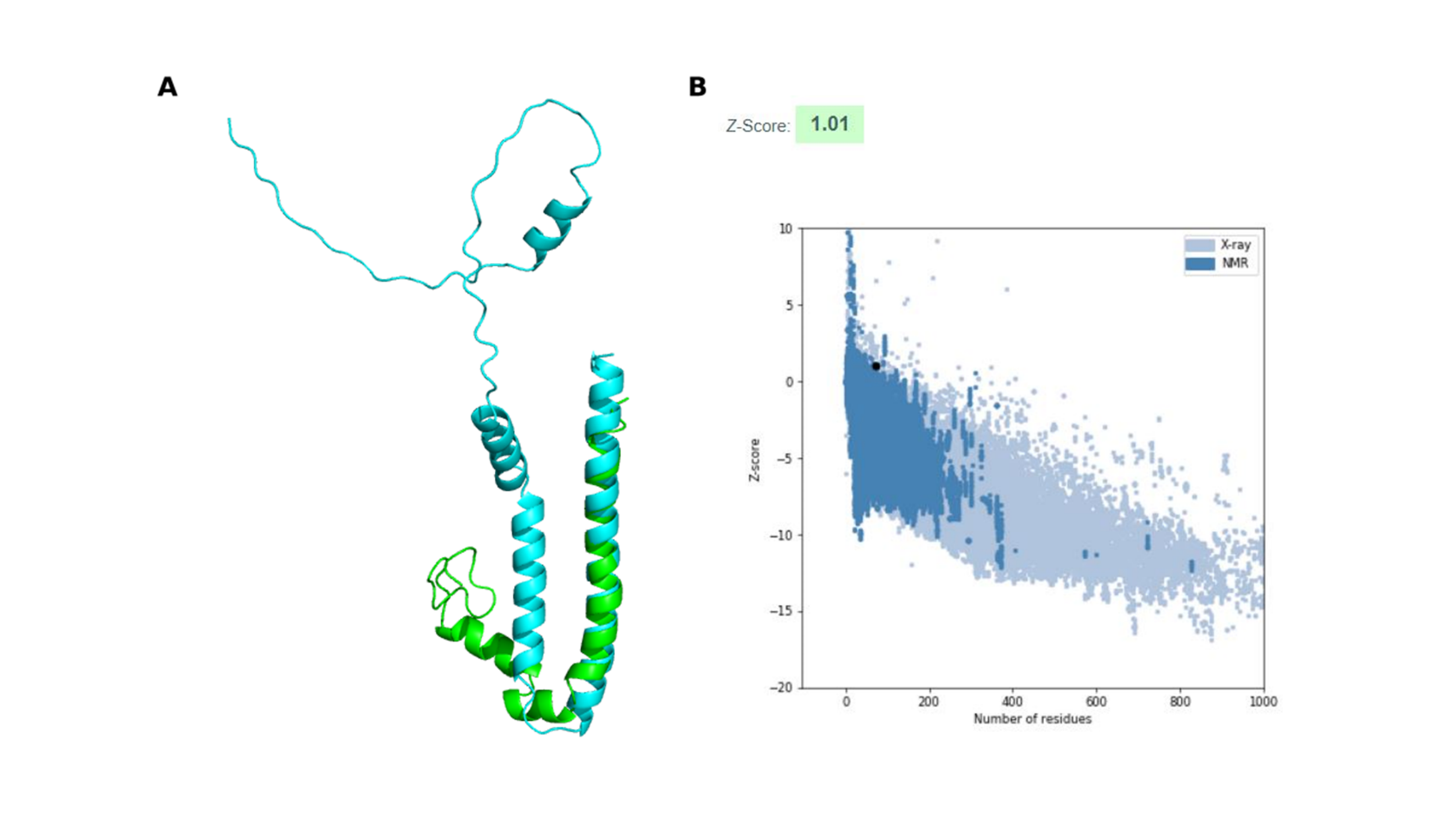


**Figure S1: (A)** Comparison of modelled IFITM3 modelled structure (Green) with AlphaFold model (Cyan) (**B**) Overall model quality of IFITM3 assessed by ProSA-web. The Z-score of all experimentally determined protein structures in PDB solved by X-ray (Light blue) or NMR (Dark blue). The Z-score of IFITM3 is represented by black dot.

**
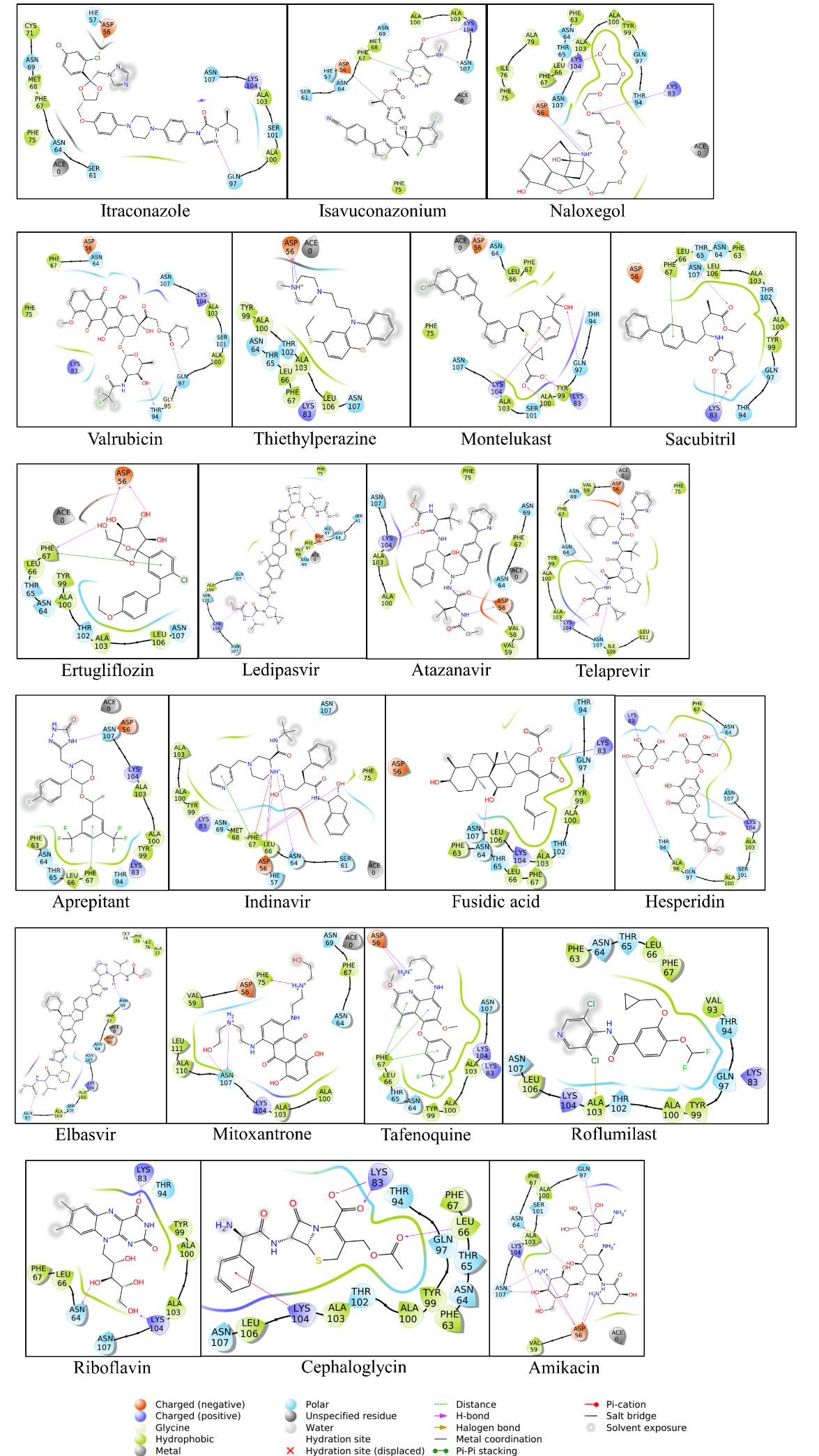
**

**Figure S2:** Interaction plot of top FDA ligands (XP docking pose) with IFITM3

**
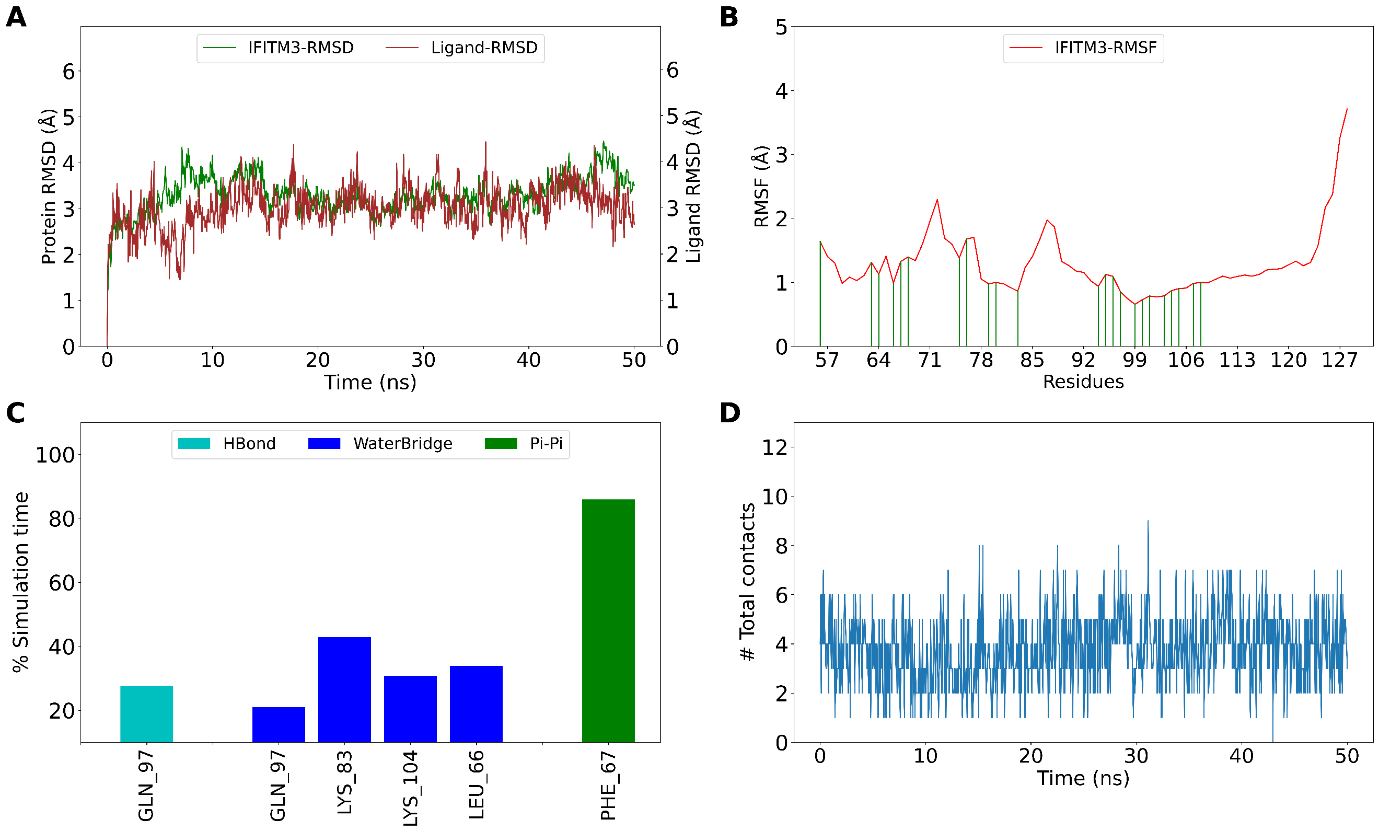
**

**Figure S3:** MD simulation of IFITM3-Valrubicin complex (**A**) RMSD of IFITM3 and Valrubicin fit on IFITM3 (Ligand-RMSD) (**B**) RMSF of IFITM3. Green lines indicate interactions with Valrubicin (**C**) Interactions between IFITM3 and Valrubicin as percentage of simulation time. Interactions that persist for more than 20% of simulation time have been shown (**D**) Total contacts (includes all interactions) between Valrubicin and IFITM3

**
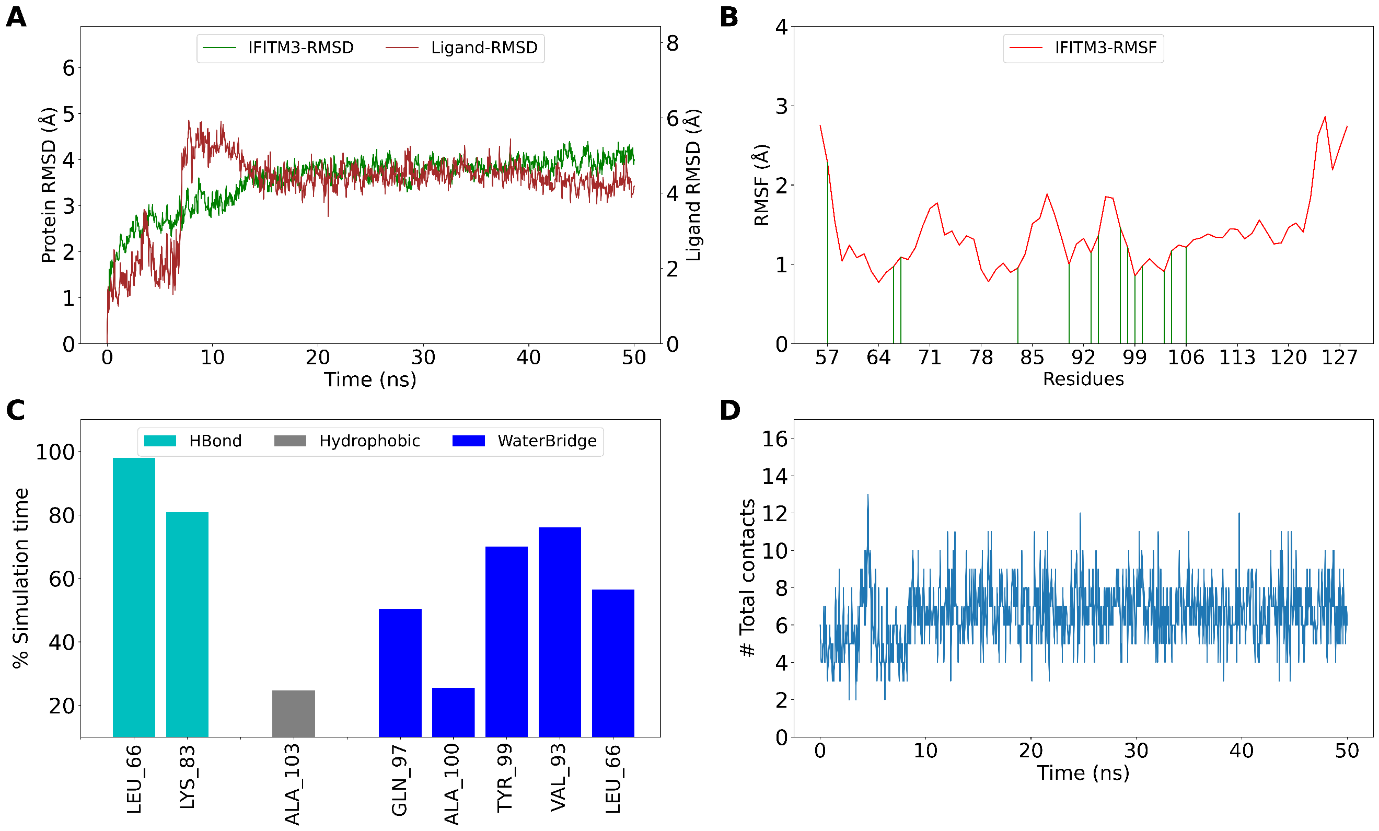
**

**Figure S4:** MD simulation of IFITM3-Sacubitril complex (**A**) RMSD of IFITM3 and Sacubitril fit on IFITM3 (Ligand-RMSD) (**B**) RMSF of IFITM3. Green lines indicate interactions with Sacubitril (**C**) Interactions between IFITM3 and Sacubitril as percentage of simulation time. Interactions that persist for more than 20% of simulation time have been shown (**D**) Total contacts (includes all interactions) between Sacubitril and IFITM3

**
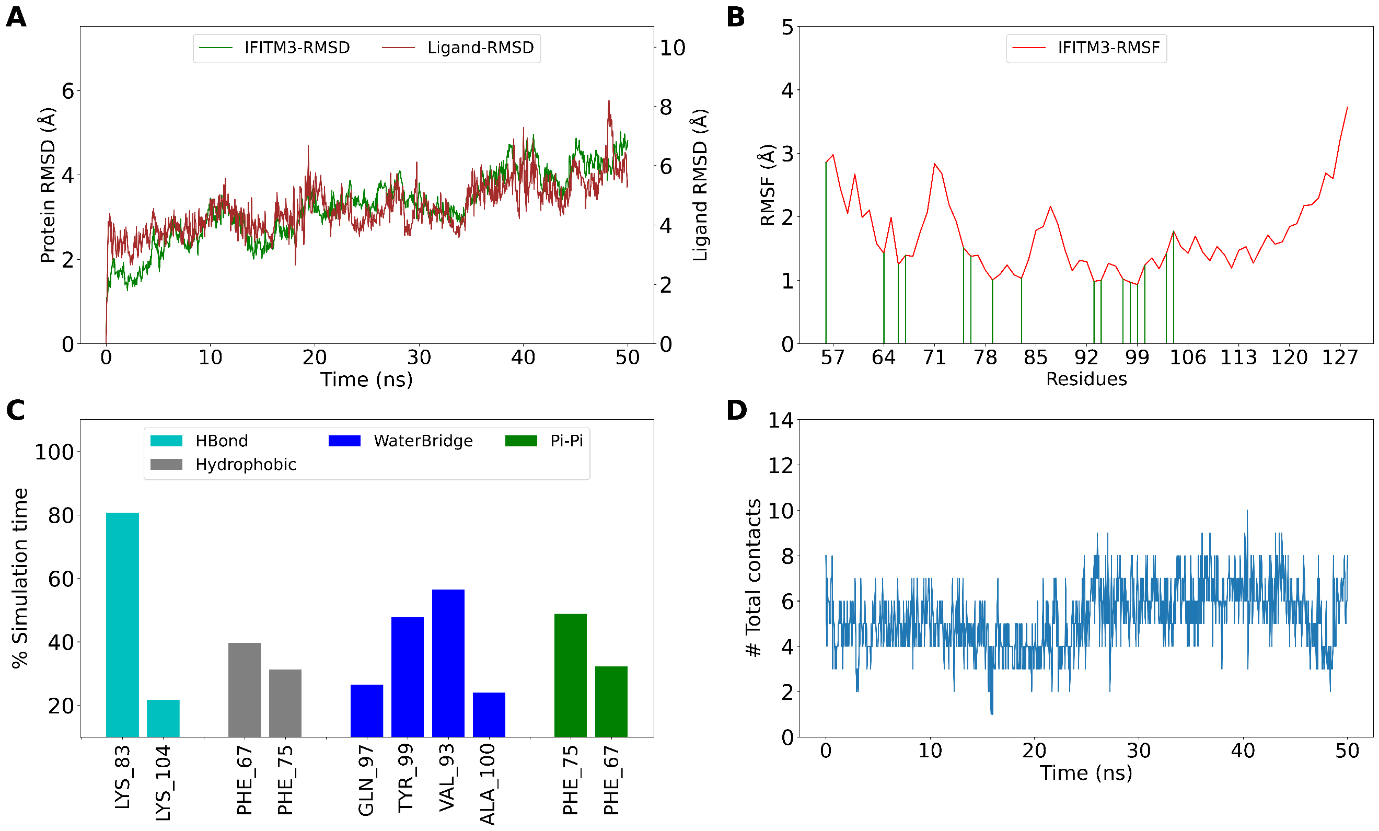
**

**Figure S5:** MD simulation of IFITM3-Montelukast complex (**A**) RMSD of IFITM3 and Montelukast fit on IFITM3 (Ligand-RMSD) (**B**) RMSF of IFITM3. Green lines indicate interactions with Montelukast (**C**) Interactions between IFITM3 and Montelukast as percentage of simulation time. Interactions that persist for more than 20% of simulation time have been shown (**D**) Total contacts (includes all interactions) between Montelukast and IFITM3

**
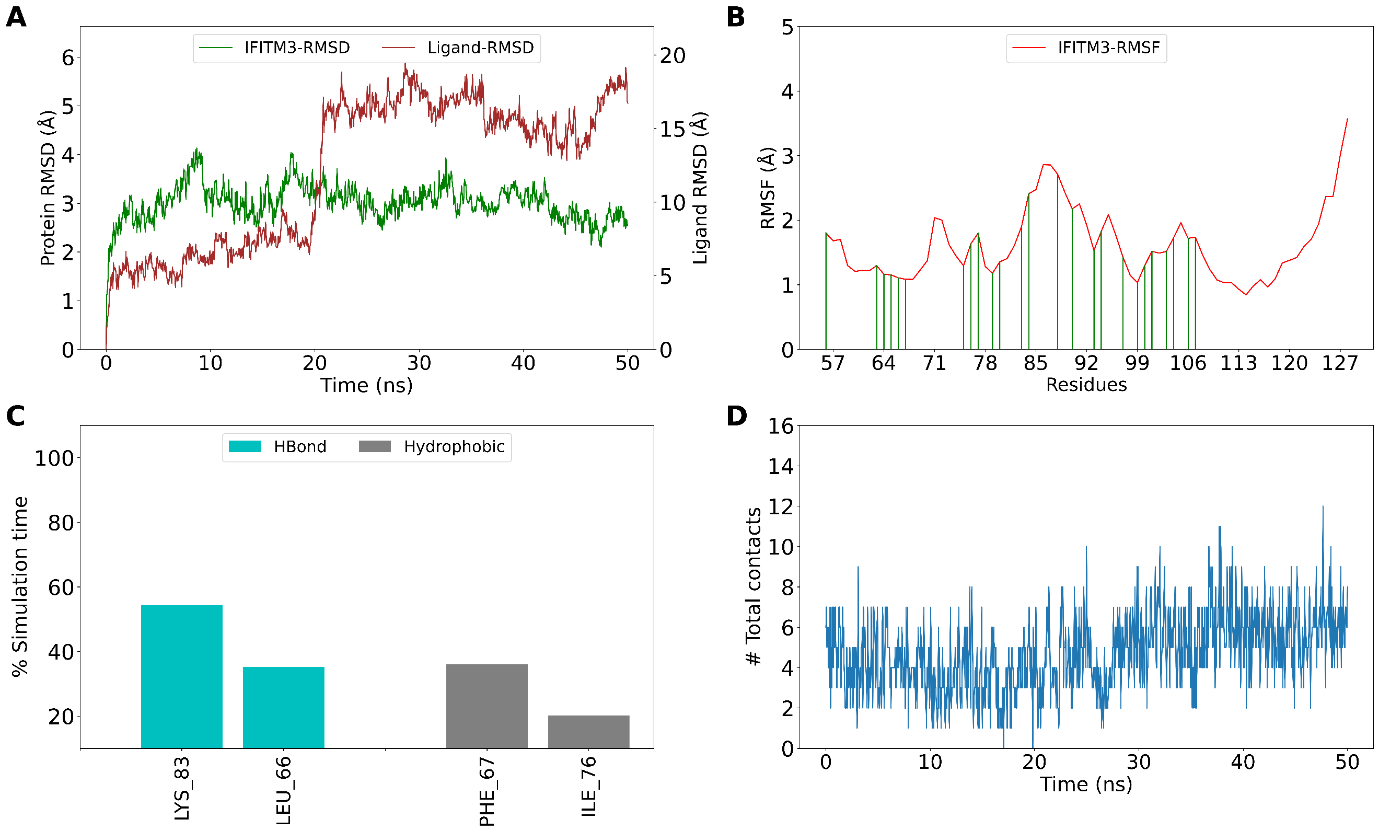
**

**Figure S6:** MD simulation of IFITM3-Naloxegol complex (**A**) RMSD of IFITM3 and Naloxegol fit on IFITM3 (Ligand-RMSD) (**B**) RMSF of IFITM3. Green lines indicate interactions with Naloxegol (**C**) Interactions between IFITM3 and Naloxegol as percentage of simulation time. Interactions that persist for more than 20% of simulation time have been shown (**D**) Total contacts (includes all interactions) between Naloxegol and IFITM3

**
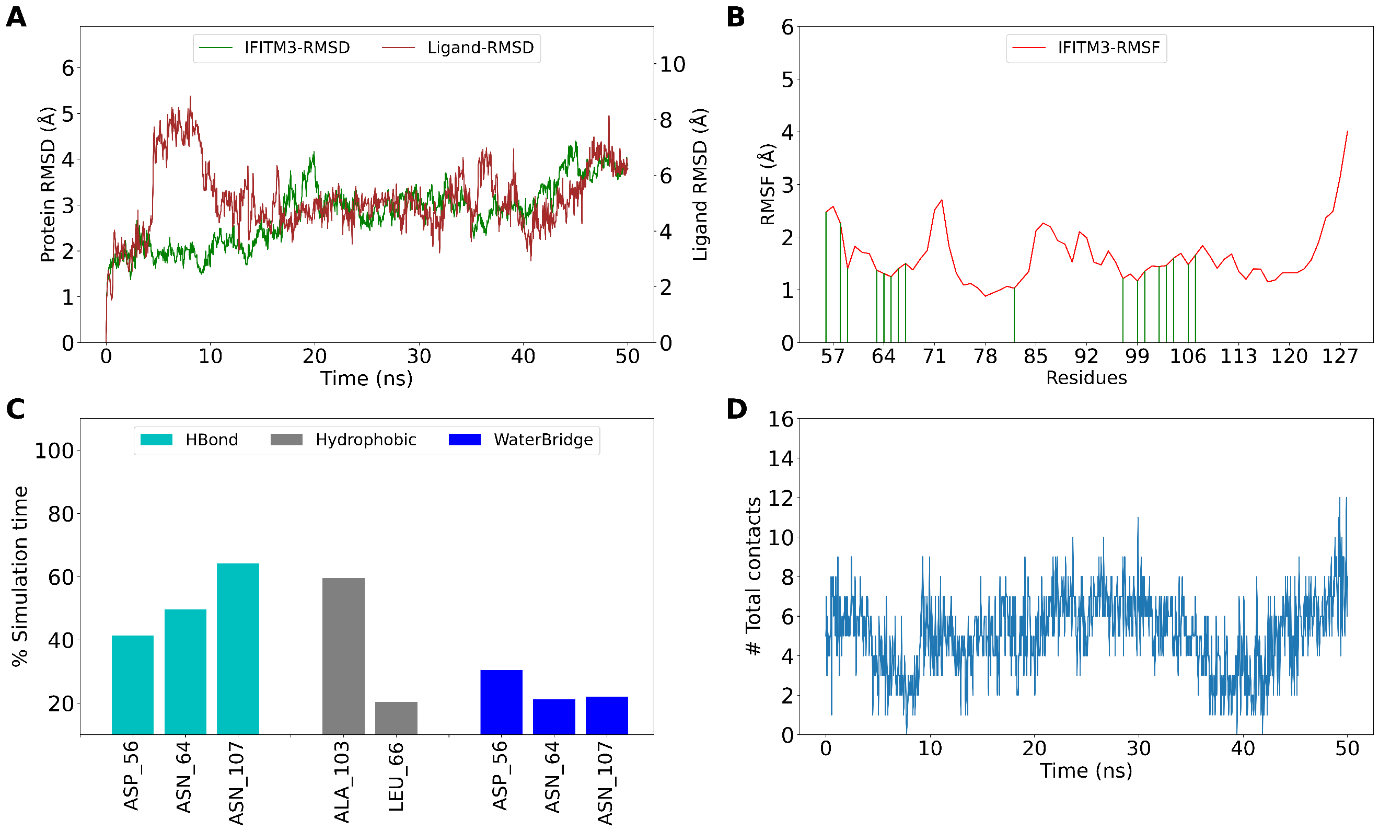
**

**Figure S7:** MD simulation of IFITM3-Ertugliflozin complex (**A**) RMSD of IFITM3 and Ertugliflozin fit on IFITM3 (Ligand-RMSD) (**B**) RMSF of IFITM3. Green lines indicate interactions with Ertugliflozin (**C**) Interactions between IFITM3 and Ertugliflozin as percentage of simulation time. Interactions that persist for more than 20% of simulation time have been shown (**D**) Total contacts (includes all interactions) between Ertugliflozin and IFITM3

**
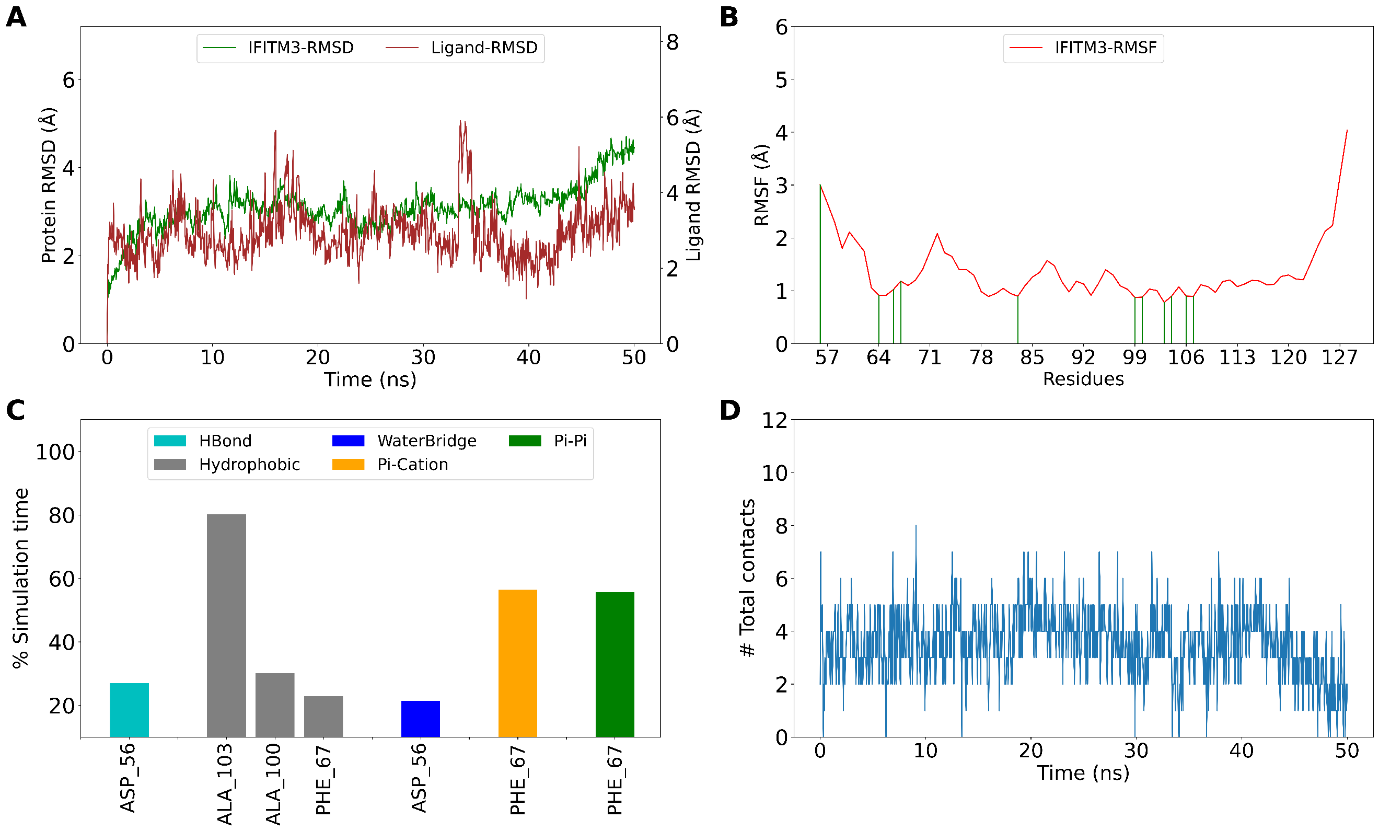
**

**Figure S8:** MD simulation of IFITM3-Thiethylperazine complex (**A**) RMSD of IFITM3 and Thiethylperazine fit on IFITM3 (Ligand-RMSD) (**B**) RMSF of IFITM3. Green lines indicate interactions with Thiethylperazine (**C**) Interactions between IFITM3 and Thiethylperazine as percentage of simulation time. Interactions that persist for more than 20% of simulation time have been shown (**D**) Total contacts (includes all interactions) between Thiethylperazine and IFITM3

**
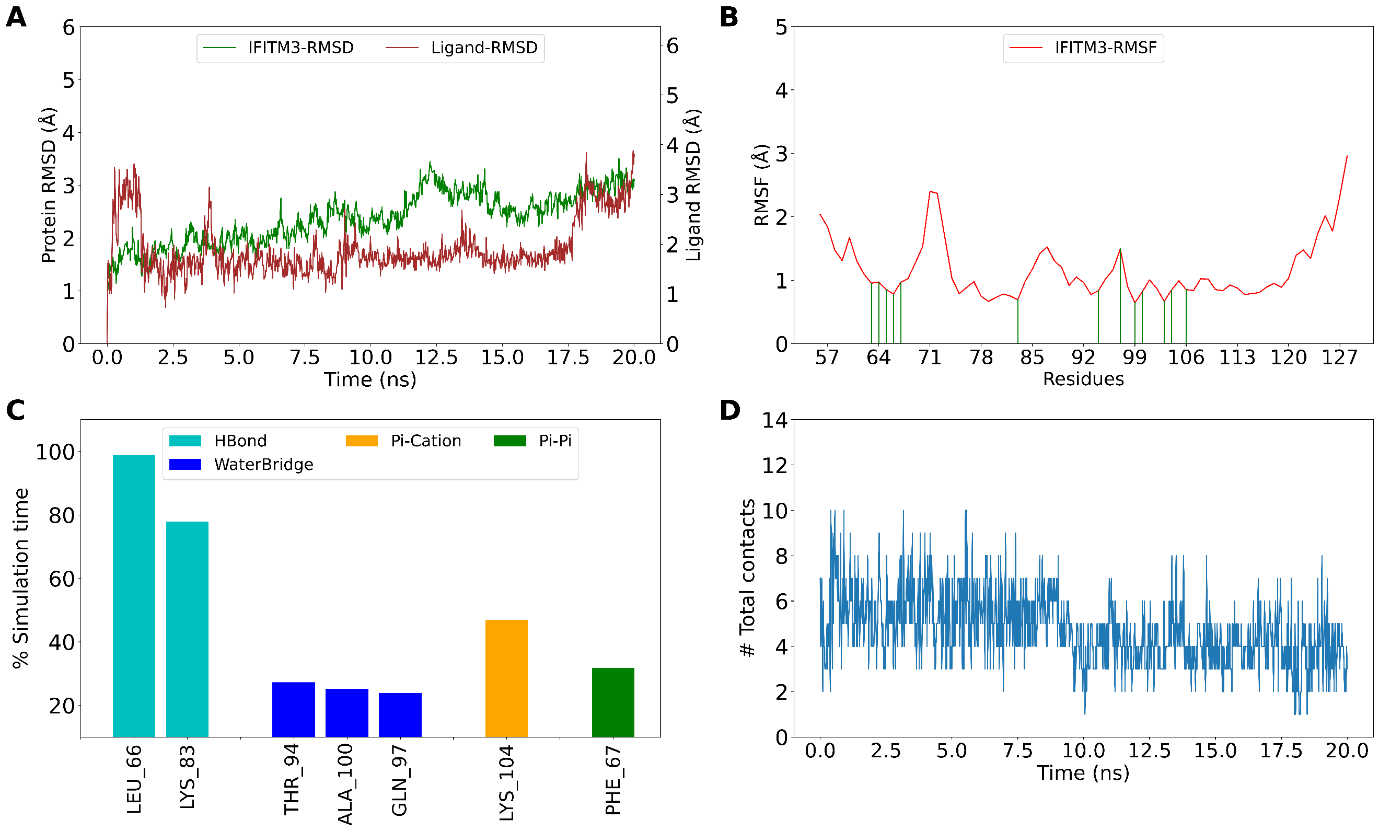
**

**Figure S9:** MD simulation of IFITM3-Cephaloglycin complex (**A**) RMSD of IFITM3 and Cephaloglycin fit on IFITM3 (Ligand-RMSD) (**B**) RMSF of IFITM3. Green lines indicate interactions with Cephaloglycin (**C**) Interactions between IFITM3 and Cephaloglycin as percentage of simulation time. Interactions that persist for more than 20% of simulation time have been shown (**D**) Total contacts (includes all interactions) between Cephaloglycin and IFITM3

**
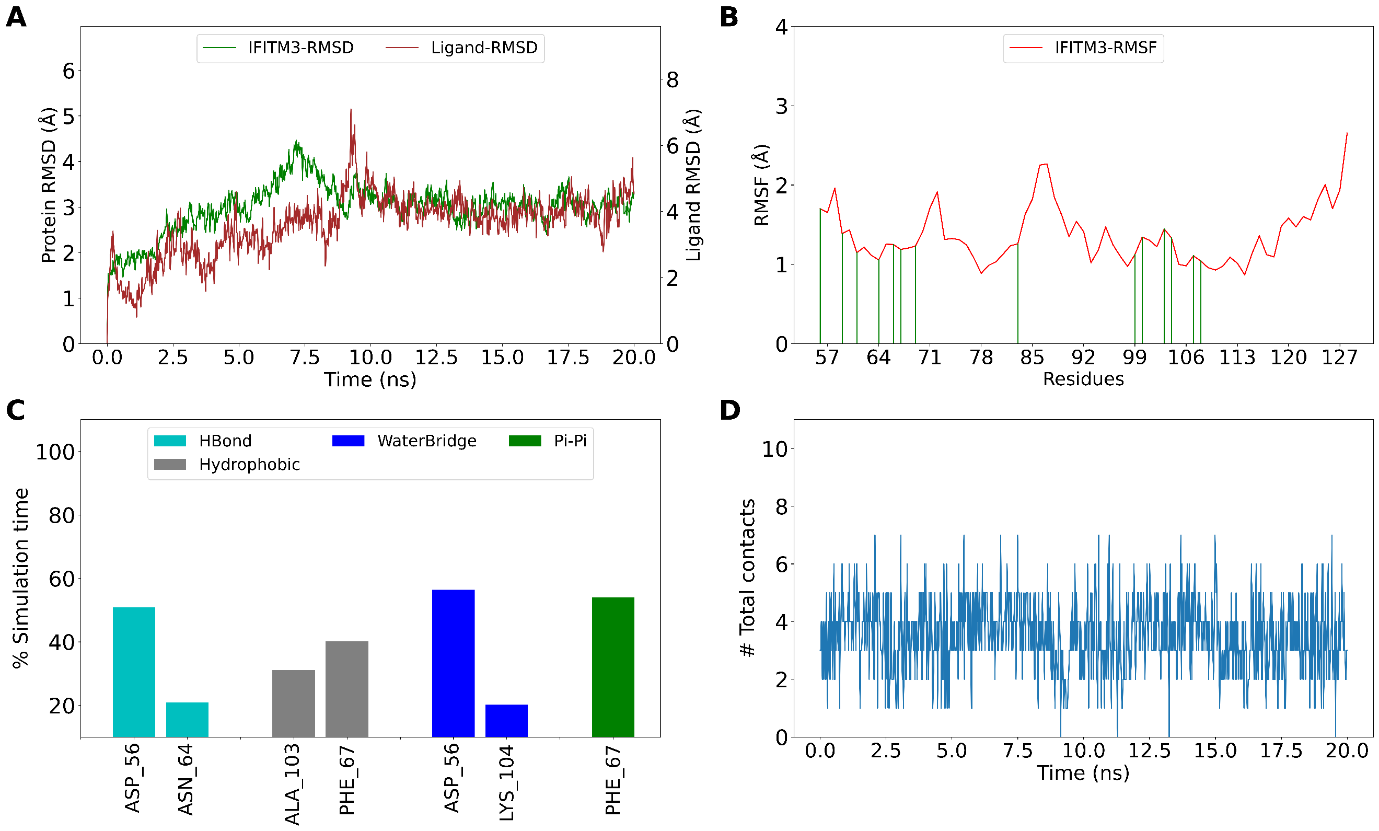
**

**Figure S10:** MD simulation of IFITM3-Aprepitant complex (**A**) RMSD of IFITM3 and Aprepitant fit on IFITM3 (Ligand-RMSD) (**B**) RMSF of IFITM3. Green lines indicate interactions with Aprepitant (**C**) Interactions between IFITM3 and Aprepitant as percentage of simulation time. Interactions that persist for more than 20% of simulation time have been shown (**D**) Total contacts (includes all interactions) between Aprepitant and IFITM3

**
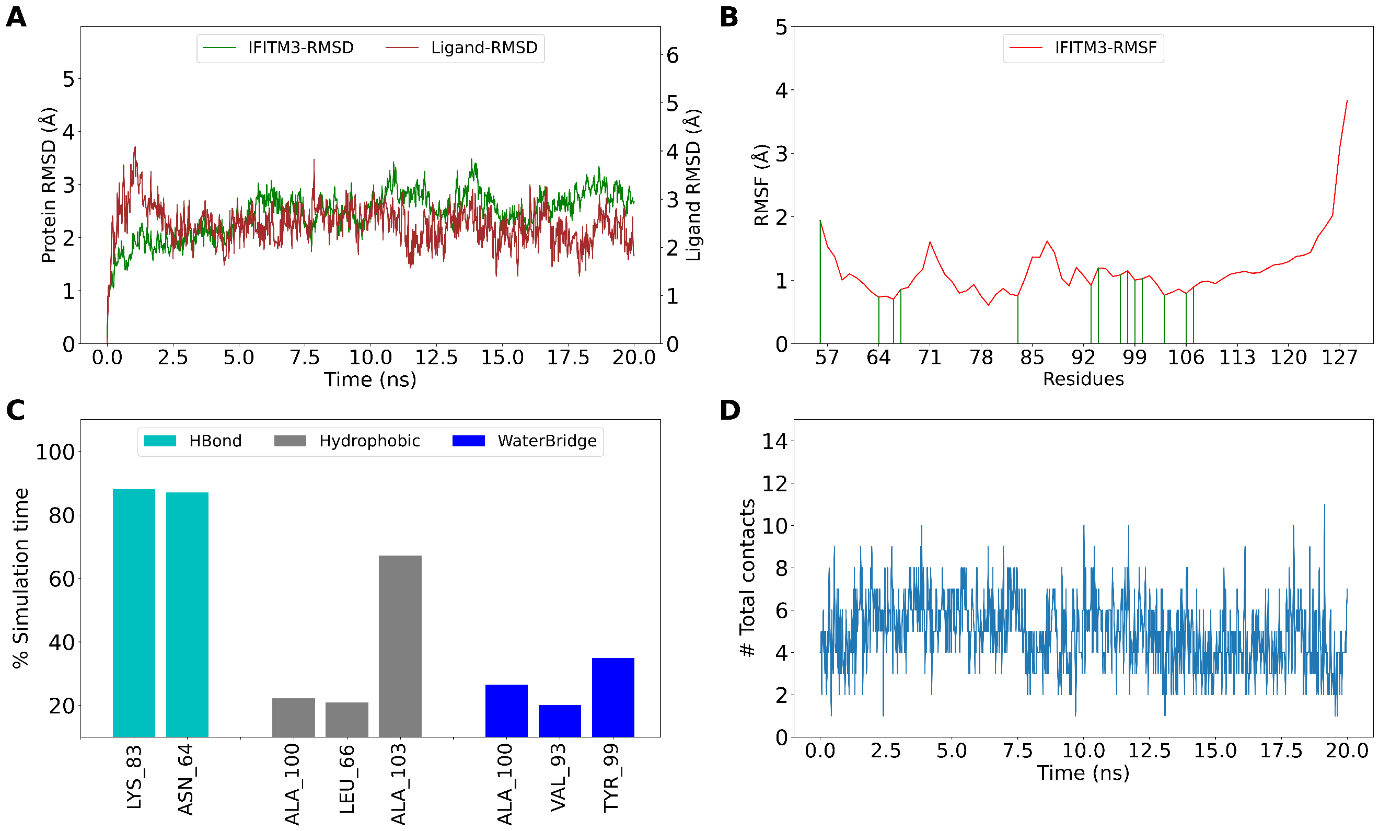
**

**Figure S11:** MD simulation of IFITM3-Fusidic acid complex (**A**) RMSD of IFITM3 and Fusidic acid fit on IFITM3 (Ligand-RMSD) (**B**) RMSF of IFITM3. Green lines indicate interactions with Fusidic acid (**C**) Interactions between IFITM3 and Fusidic acid as percentage of simulation time. Interactions that persist for more than 20% of simulation time have been shown (**D**) Total contacts (includes all interactions) between Fusidic acid and IFITM3

**
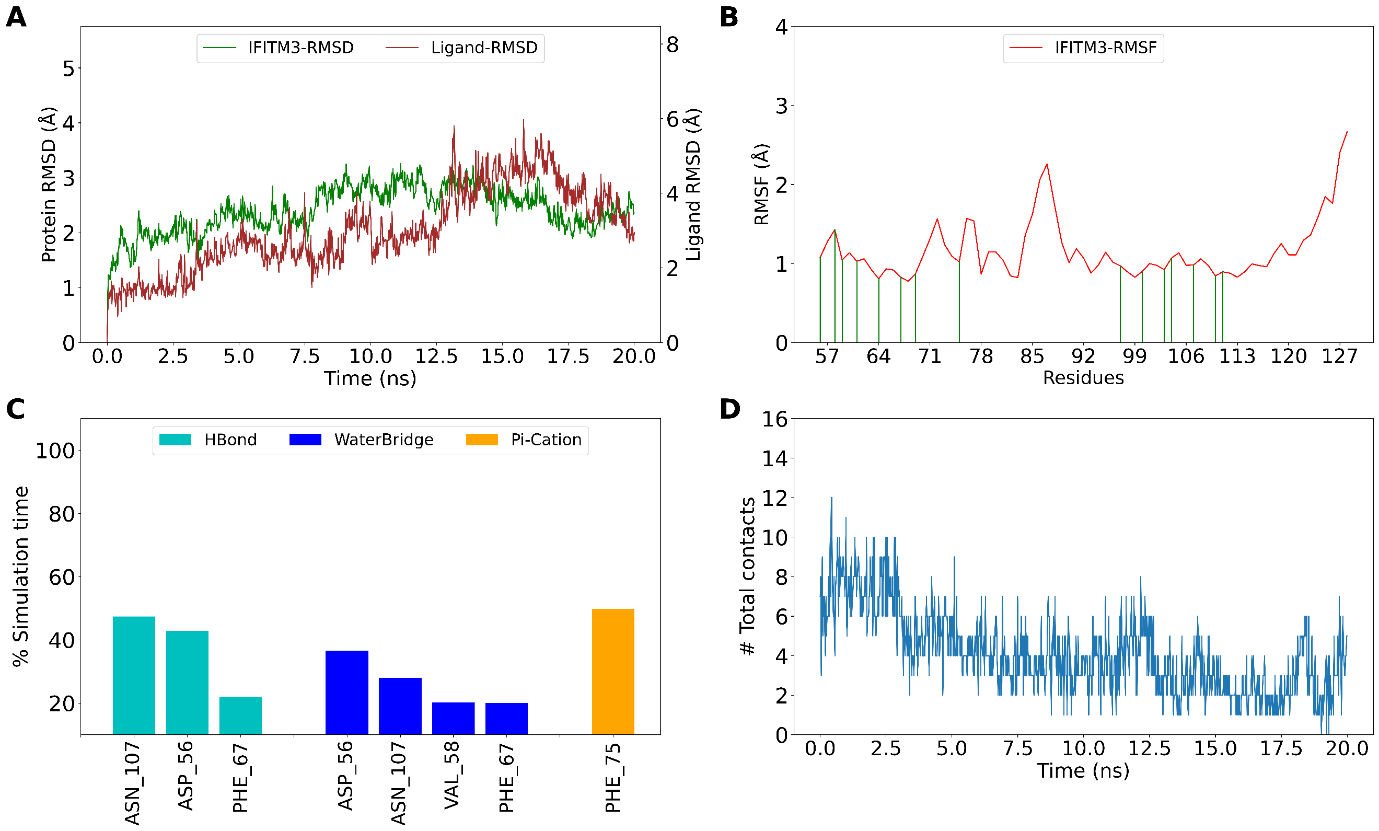
**

**Figure S12:** MD simulation of IFITM3-Mitoxantrone complex (**A**) RMSD of IFITM3 and Mitoxantrone fit on IFITM3 (Ligand-RMSD) (**B**) RMSF of IFITM3. Green lines indicate interactions with Mitoxantrone (**C**) Interactions between IFITM3 and Mitoxantrone as percentage of simulation time. Interactions that persist for more than 20% of simulation time have been shown (**D**) Total contacts (includes all interactions) between Mitoxantrone and IFITM3

**
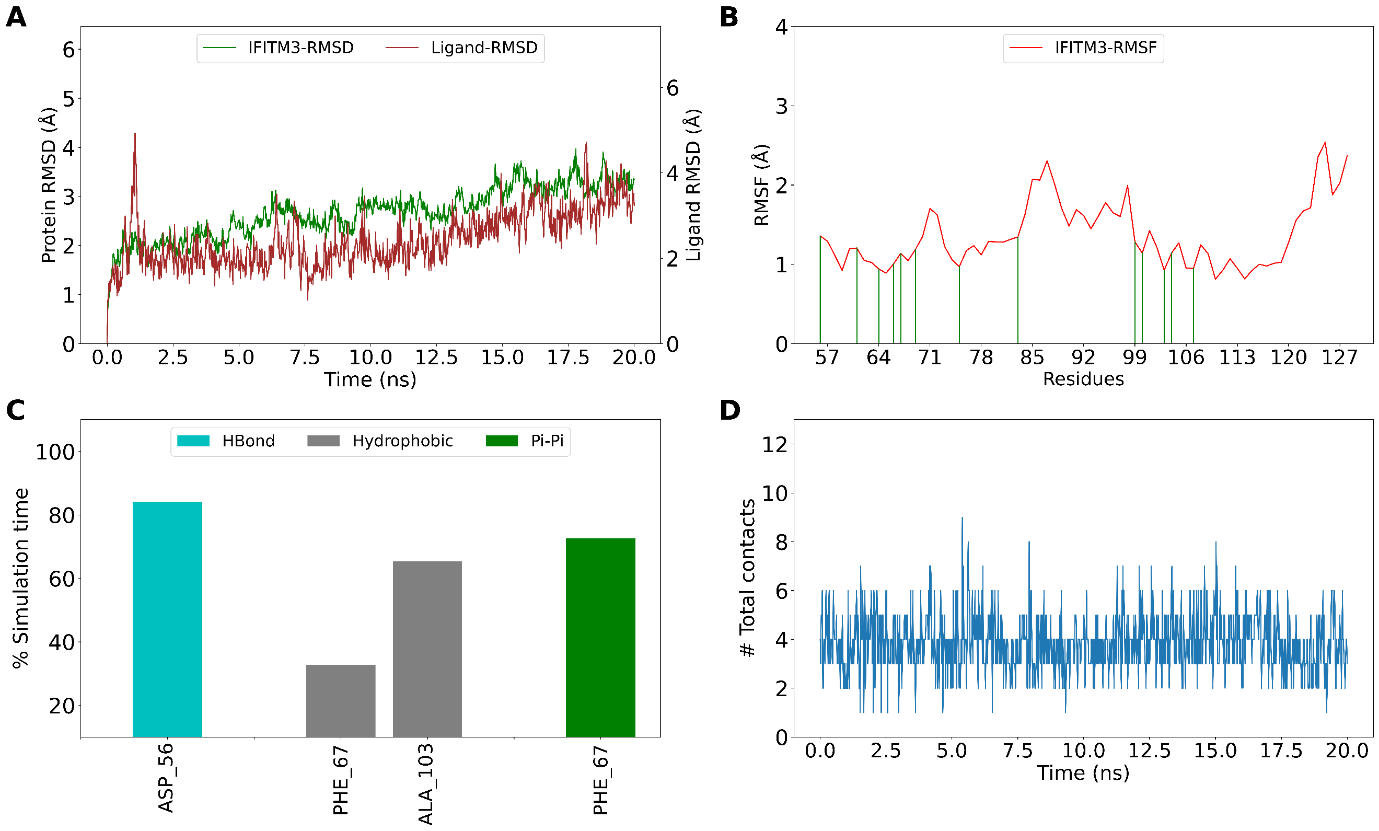
**

**Figure S13:** MD simulation of IFITM3-Tafenoquine complex (**A**) RMSD of IFITM3 and Tafenoquine fit on IFITM3 (Ligand-RMSD) (**B**) RMSF of IFITM3. Green lines indicate interactions with Tafenoquine (**C**) Interactions between IFITM3 and Tafenoquine as percentage of simulation time. Interactions that persist for more than 20% of simulation time have been shown (**D**) Total contacts (includes all interactions) between Tafenoquine and IFITM3

**
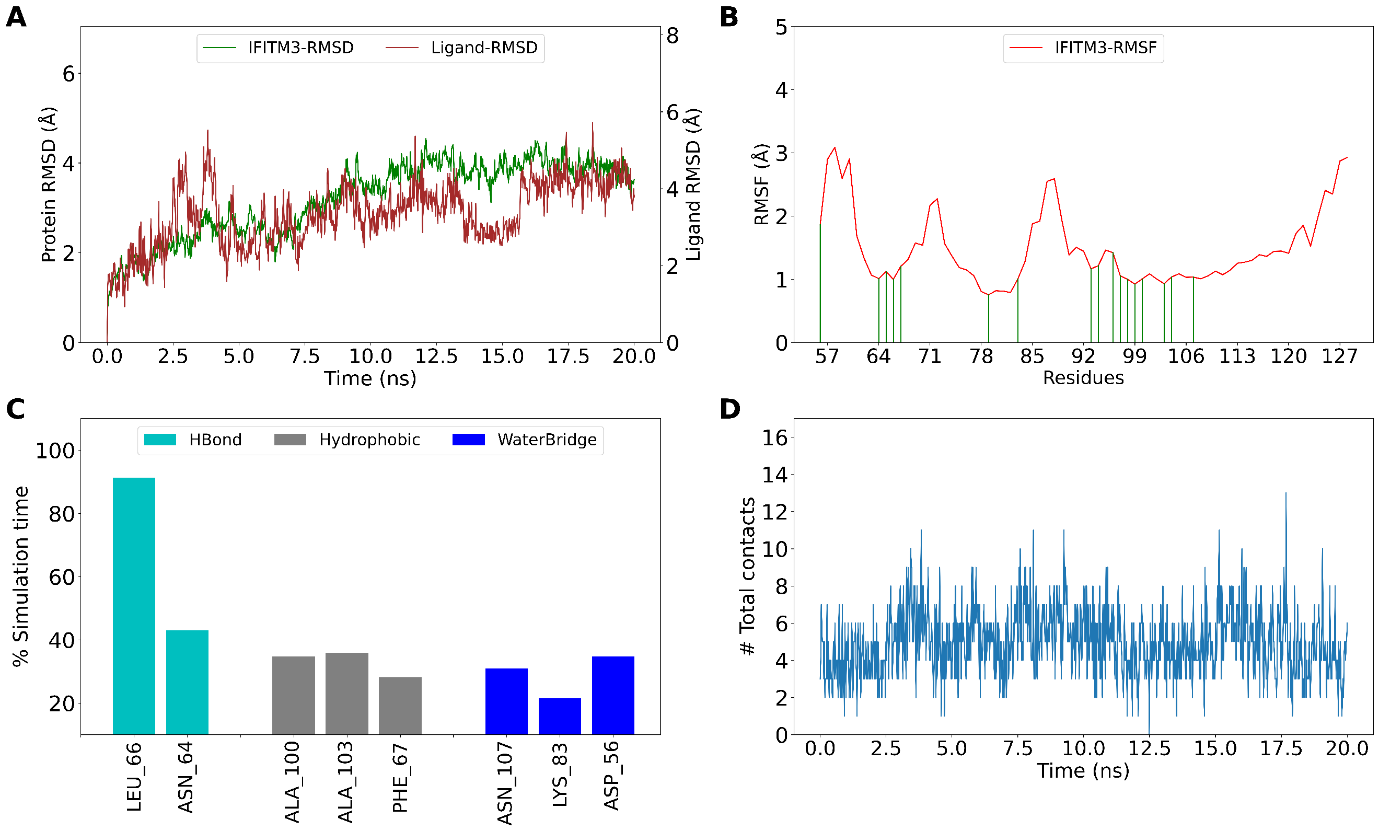
**

**Figure S14:** MD simulation of IFITM3-Riboflavin complex (**A**) RMSD of IFITM3 and Riboflavin fit on IFITM3 (Ligand-RMSD) (**B**) RMSF of IFITM3. Green lines indicate interactions with Riboflavin (**C**) Interactions between IFITM3 and Riboflavin as percentage of simulation time. Interactions that persist for more than 20% of simulation time have been shown (**D**) Total contacts (includes all interactions) between Riboflavin and IFITM3

**
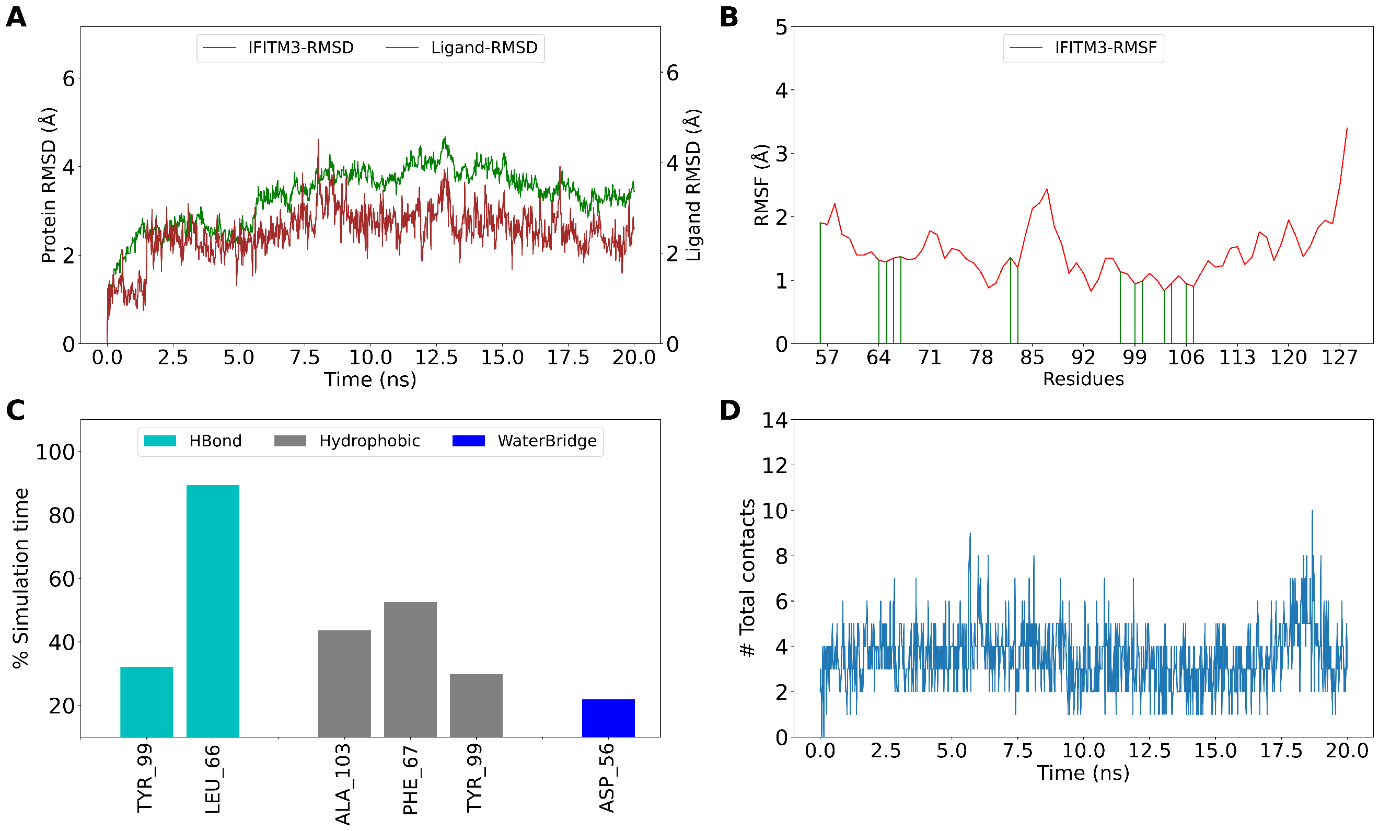
**

**Figure S15:** MD simulation of IFITM3-Roflumilast complex (**A**) RMSD of IFITM3 and Roflumilast fit on IFITM3 (Ligand-RMSD) (**B**) RMSF of IFITM3. Green lines indicate interactions with Roflumilast (**C**) Interactions between IFITM3 and Roflumilast as percentage of simulation time. Interactions that persist for more than 20% of simulation time have been shown (**D**) Total contacts (includes all interactions) between Roflumilast and IFITM3

**
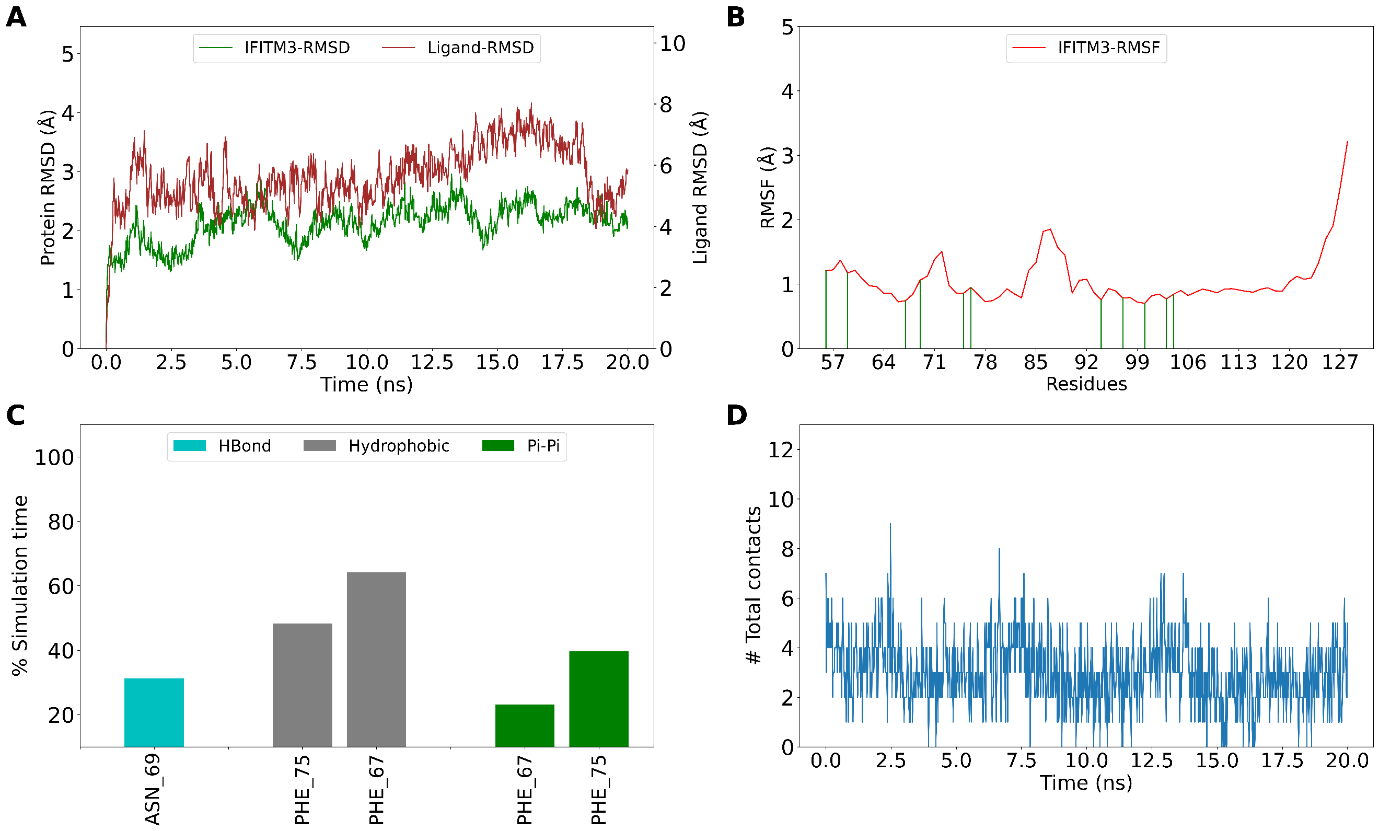
**

**Figure S16:** MD simulation of IFITM3-Atazanavir complex (**A**) RMSD of IFITM3 and Atazanavir fit on IFITM3 (Ligand-RMSD) (**B**) RMSF of IFITM3. Green lines indicate interactions with Atazanavir (**C**) Interactions between IFITM3 and Atazanavir as percentage of simulation time. Interactions that persist for more than 20% of simulation time have been shown (**D**) Total contacts (includes all interactions) between Atazanavir and IFITM3

**
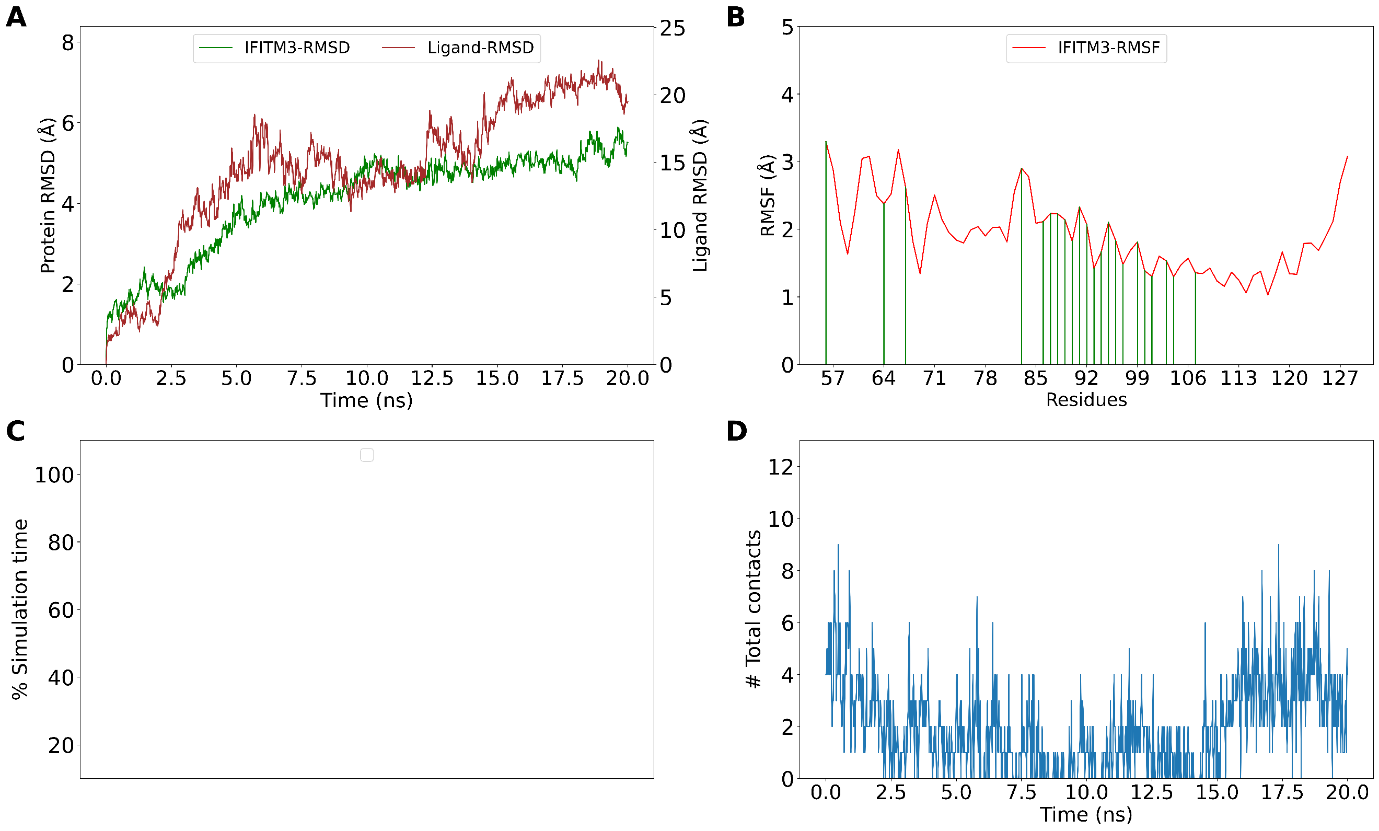
**

**Figure S17:** MD simulation of IFITM3-Hesperidin complex (**A**) RMSD of IFITM3 and Hesperidin fit on IFITM3 (Ligand-RMSD) (**B**) RMSF of IFITM3. Green lines indicate interactions with Hesperidin (**C**) Interactions between IFITM3 and Hesperidin as percentage of simulation time. Interactions that persist for more than 20% of simulation time have been shown (**D**) Total contacts (includes all interactions) between Hesperidin and IFITM3

**
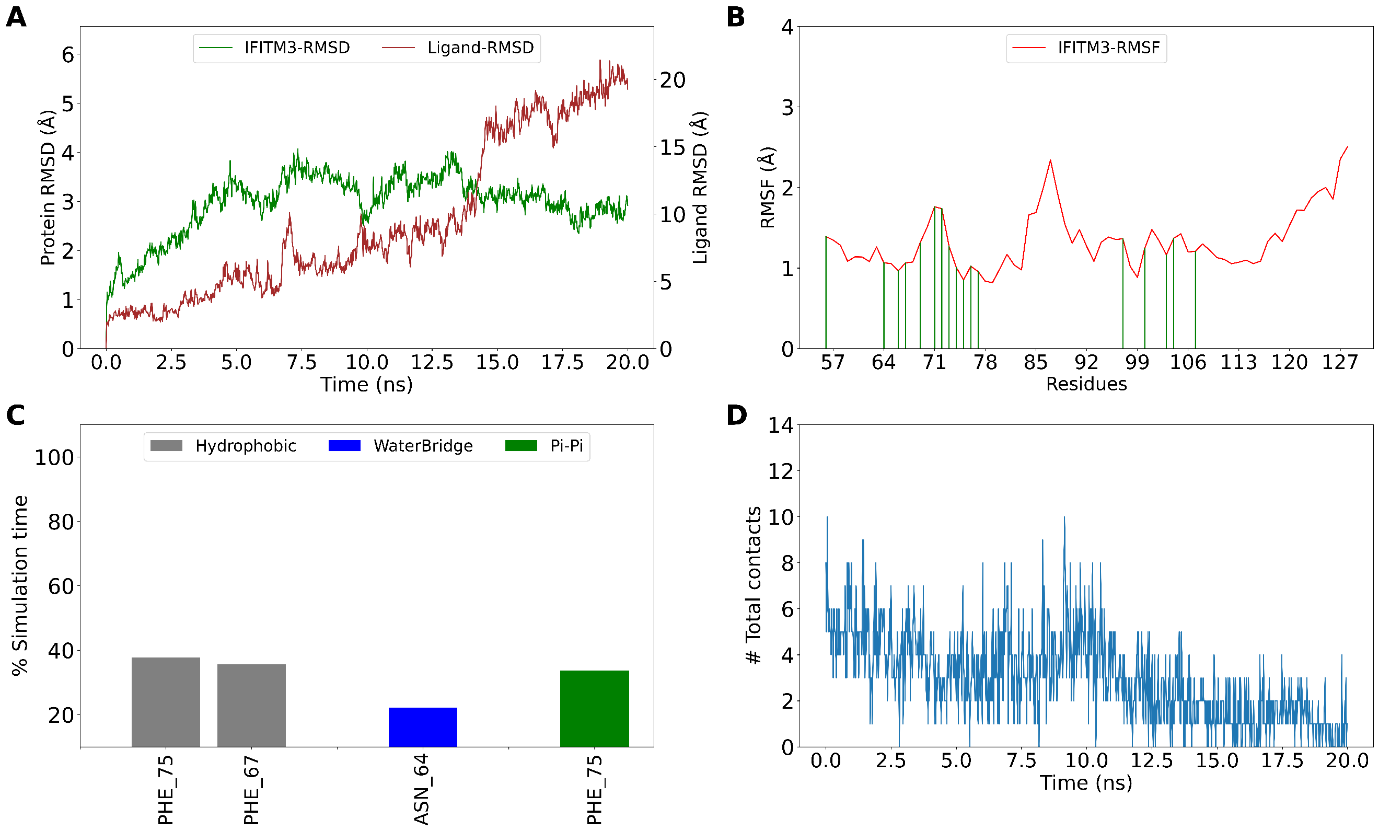
**

**Figure S18:** MD simulation of IFITM3-Elbasvir complex (**A**) RMSD of IFITM3 and Elbasvir fit on IFITM3 (Ligand-RMSD) (**B**) RMSF of IFITM3. Green lines indicate interactions with Elbasvir (**C**) Interactions between IFITM3 and Elbasvir as percentage of simulation time. Interactions that persist for more than 20% of simulation time have been shown (**D**) Total contacts (includes all interactions) between Elbasvir and IFITM3

**
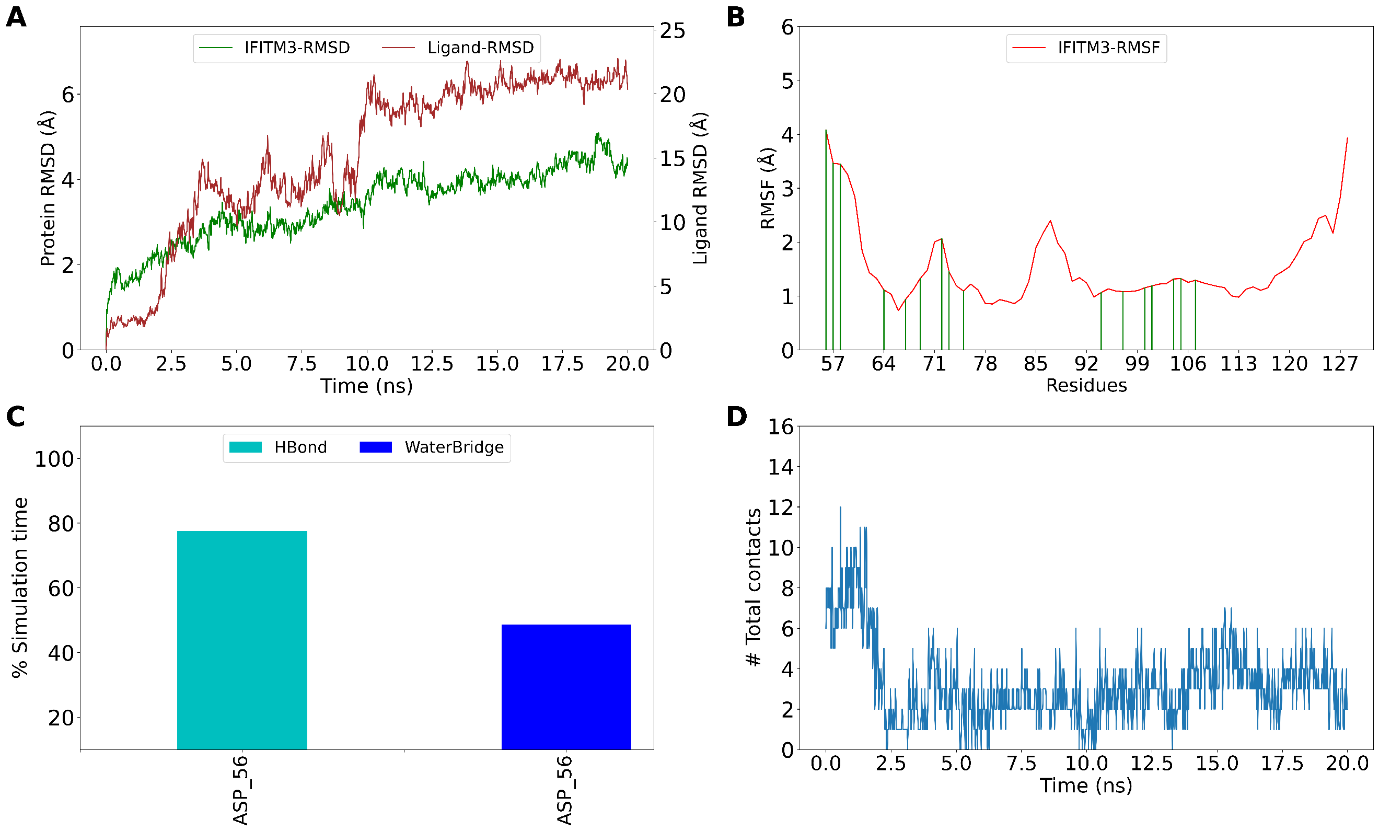
**

**Figure S19:** MD simulation of IFITM3-Amikacin complex (**A**) RMSD of IFITM3 and Amikacin fit on IFITM3 (Ligand-RMSD) (**B**) RMSF of IFITM3. Green lines indicate interactions with Amikacin (**C**) Interactions between IFITM3 and Amikacin as percentage of simulation time. Interactions that persist for more than 20% of simulation time have been shown (**D**) Total contacts (includes all interactions) between Amikacin and IFITM3

**
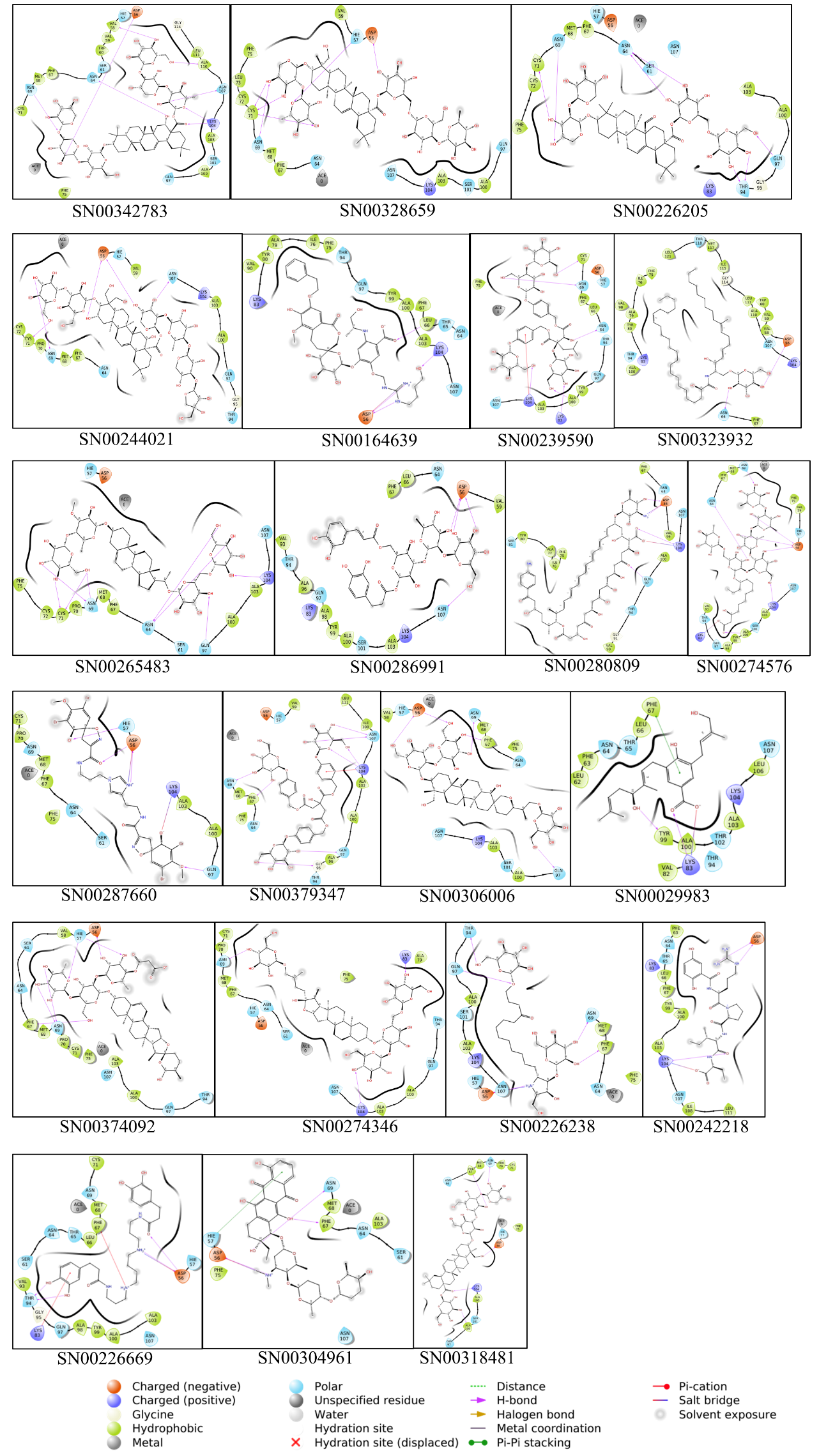
**

**Figure S20:** Interaction plot of SNDB ligands (XP docking pose) with IFITM3

**
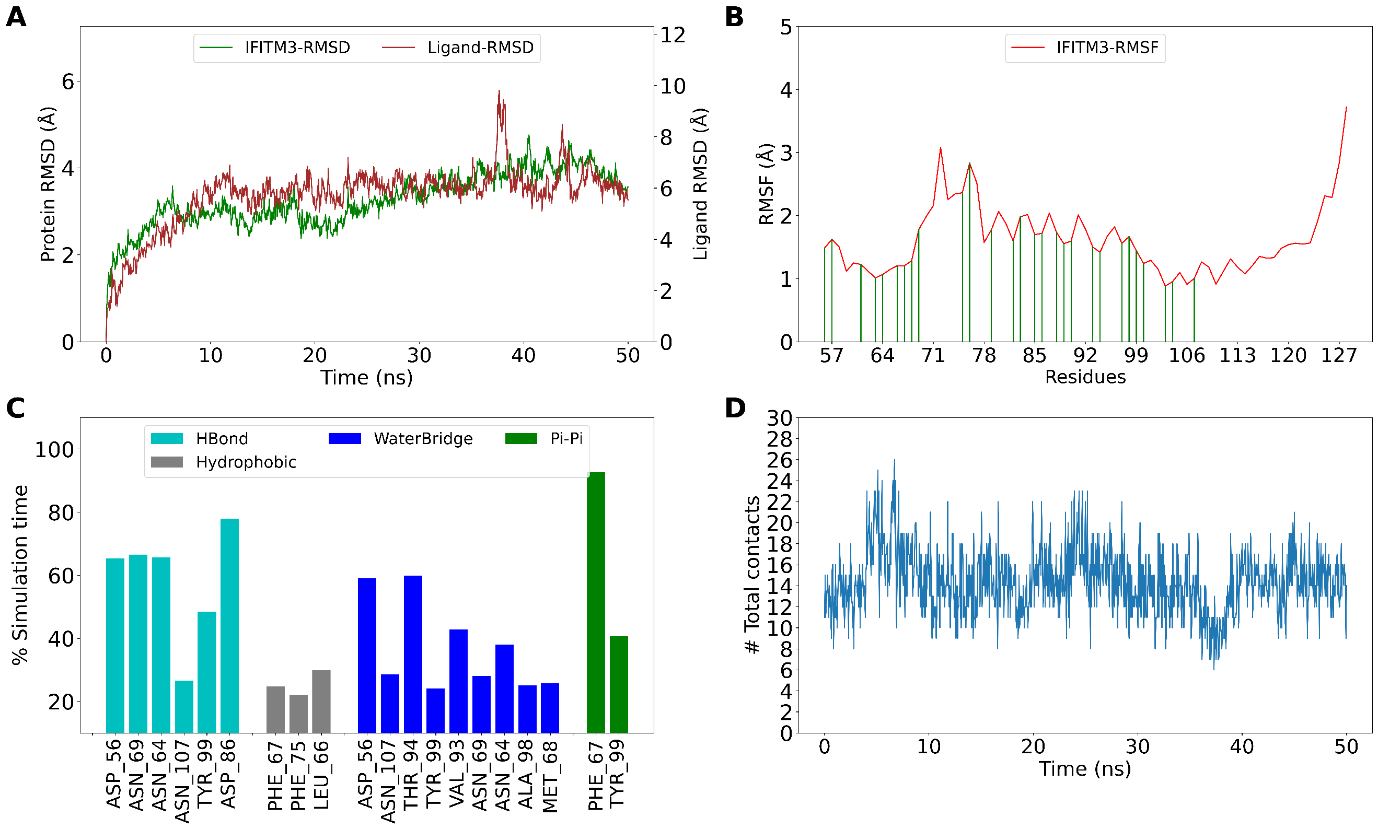
**

**Figure S21:** MD simulation of IFITM3-SN00249458 complex (**A**) RMSD of IFITM3 and SN00249458 fit on IFITM3 (Ligand-RMSD) (**B**) RMSF of IFITM3. Green lines indicate interactions with SN00249458 (**C**) Interactions between IFITM3 and SN00249458 as percentage of simulation time. Interactions that persist for more than 20% of simulation time have been shown (**D**) Total contacts (includes all interactions) between SN00249458 and IFITM3

**
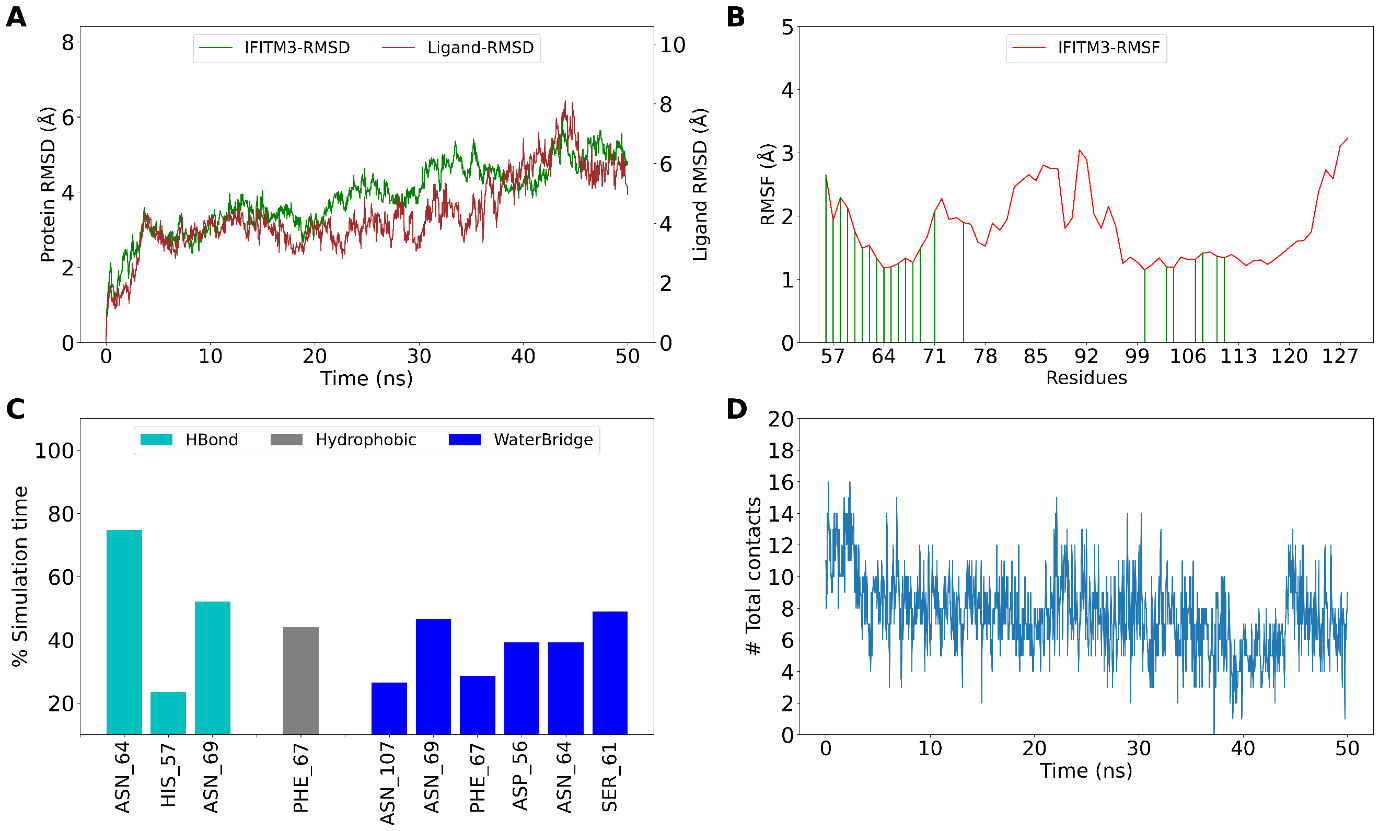
**

**Figure S22:** MD simulation of IFITM3-SN00342783 complex (**A**) RMSD of IFITM3 and SN00342783 fit on IFITM3 (Ligand-RMSD) (**B**) RMSF of IFITM3. Green lines indicate interactions with SN00342783 (**C**) Interactions between IFITM3 and SN00342783 as percentage of simulation time. Interactions that persist for more than 20% of simulation time have been shown (**D**) Total contacts (includes all interactions) between SN00342783 and IFITM3

**
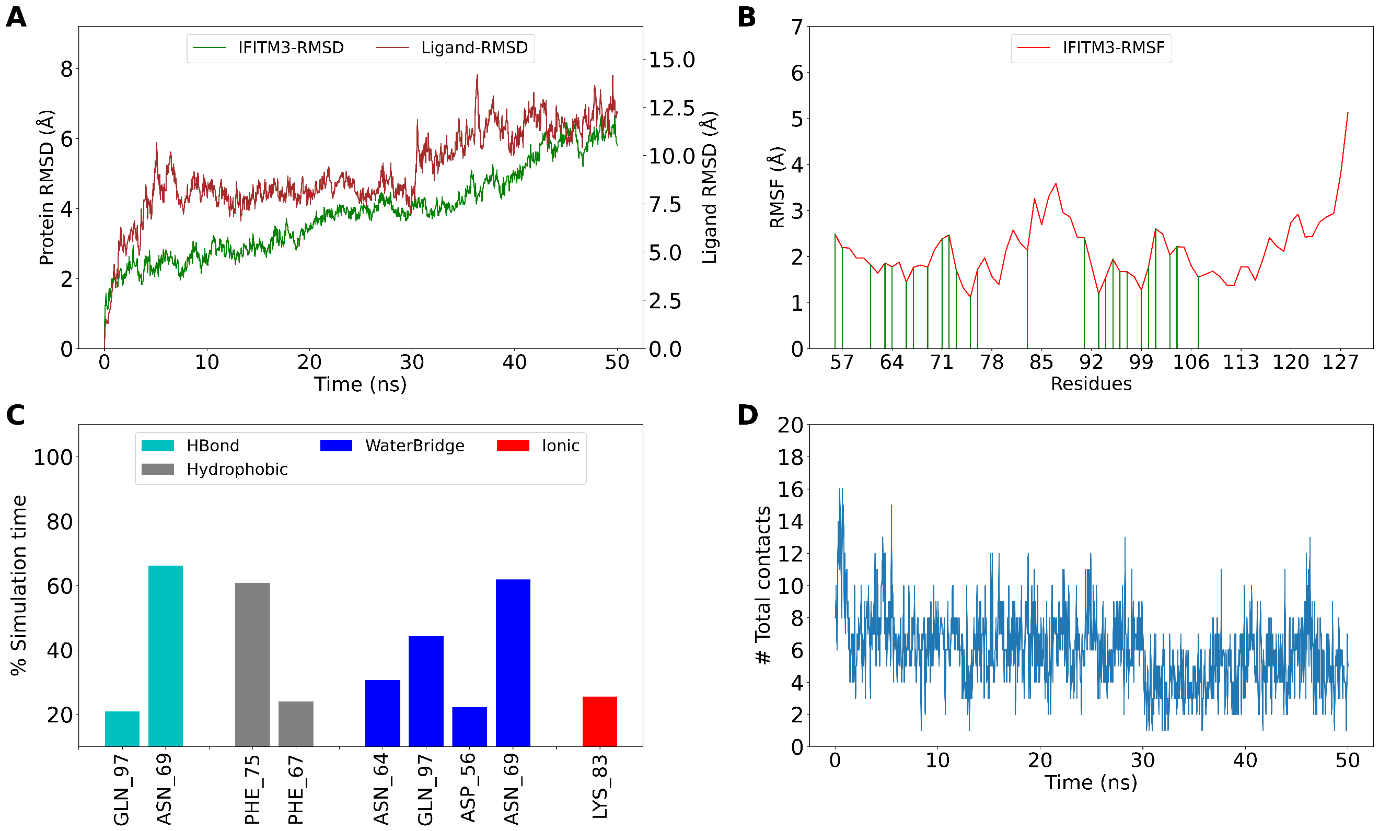
**

**Figure S23:** MD simulation of IFITM3-SN00328659 complex (**A**) RMSD of IFITM3 and SN00328659 fit on IFITM3 (Ligand-RMSD) (**B**) RMSF of IFITM3. Green lines indicate interactions with SN00328659 (**C**) Interactions between IFITM3 and SN00328659 as percentage of simulation time. Interactions that persist for more than 20% of simulation time have been shown (**D**) Total contacts (includes all interactions) between SN00328659 and IFITM3

**
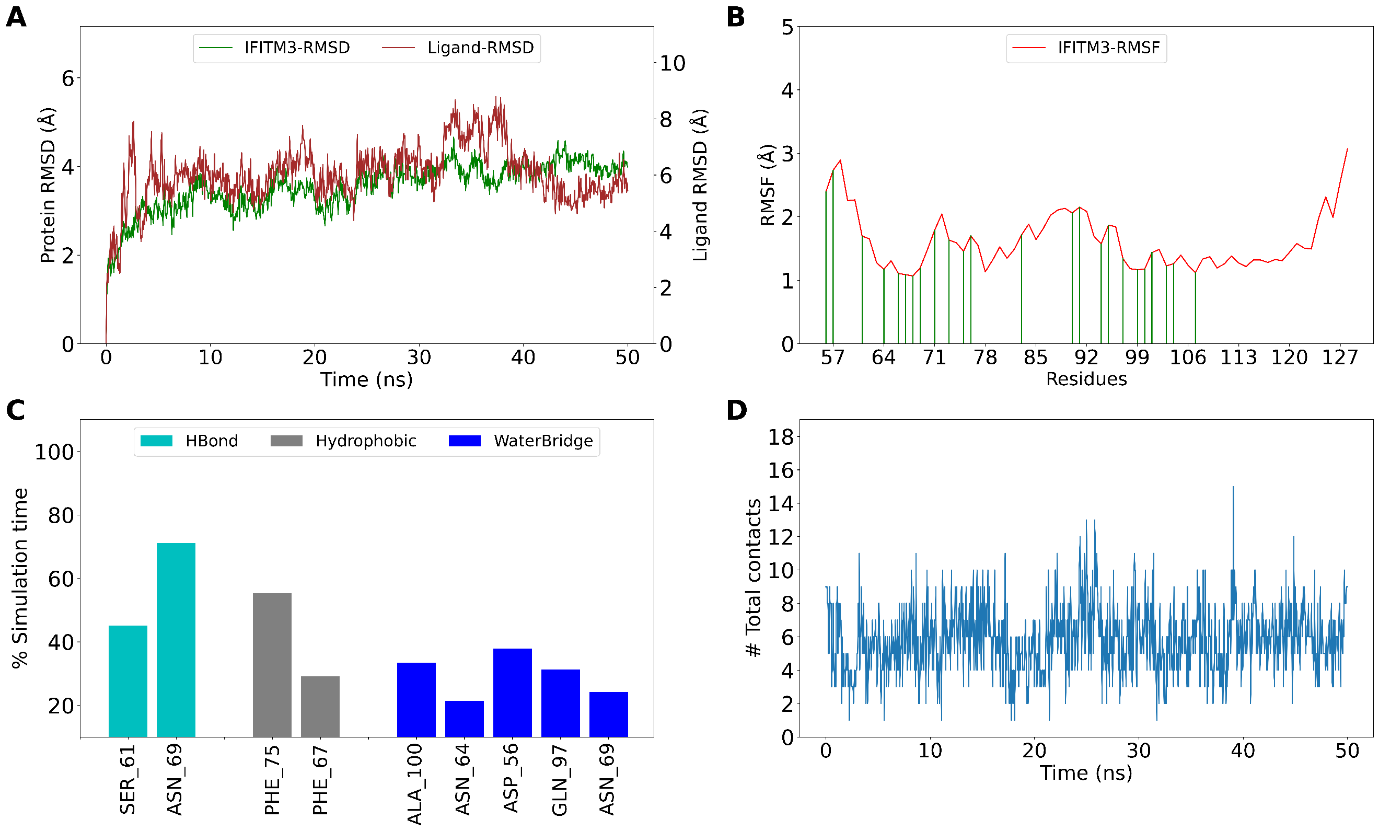
**

**Figure S24:** MD simulation of IFITM3-SN00226205 complex (**A**) RMSD of IFITM3 and SN00226205 fit on IFITM3 (Ligand-RMSD) (**B**) RMSF of IFITM3. Green lines indicate interactions with SN00226205 (**C**) Interactions between IFITM3 and SN00226205 as percentage of simulation time. Interactions that persist for more than 20% of simulation time have been shown (**D**) Total contacts (includes all interactions) between SN00226205 and IFITM3

**
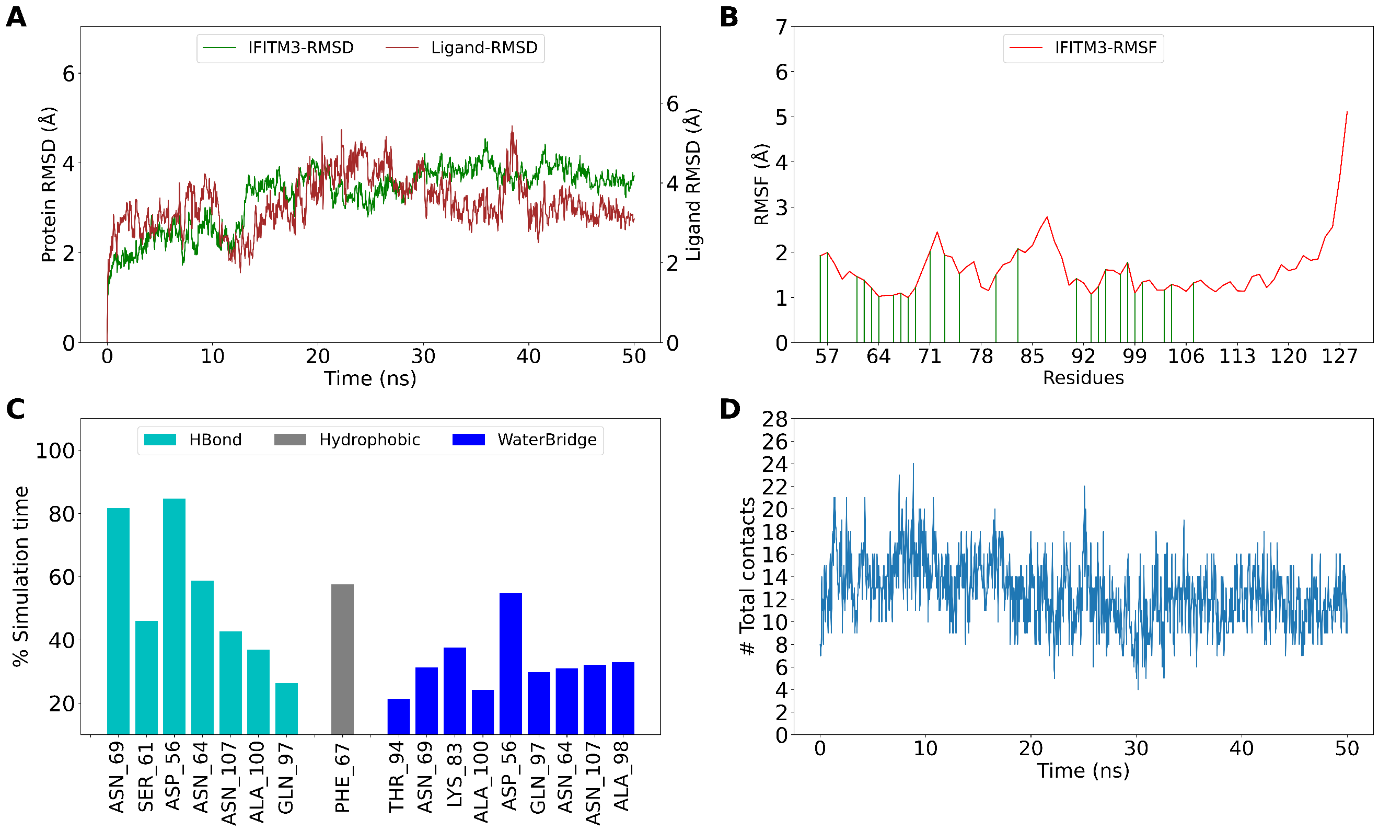
**

**Figure S25:** MD simulation of IFITM3-SN00244021complex (**A**) RMSD of IFITM3 and SN00244021 fit on IFITM3 (Ligand-RMSD) (**B**) RMSF of IFITM3. Green lines indicate interactions with SN00244021 (**C**) Interactions between IFITM3 and SN00244021 as percentage of simulation time. Interactions that persist for more than 20% of simulation time have been shown (**D**) Total contacts (includes all interactions) between SN00244021 and IFITM3

**
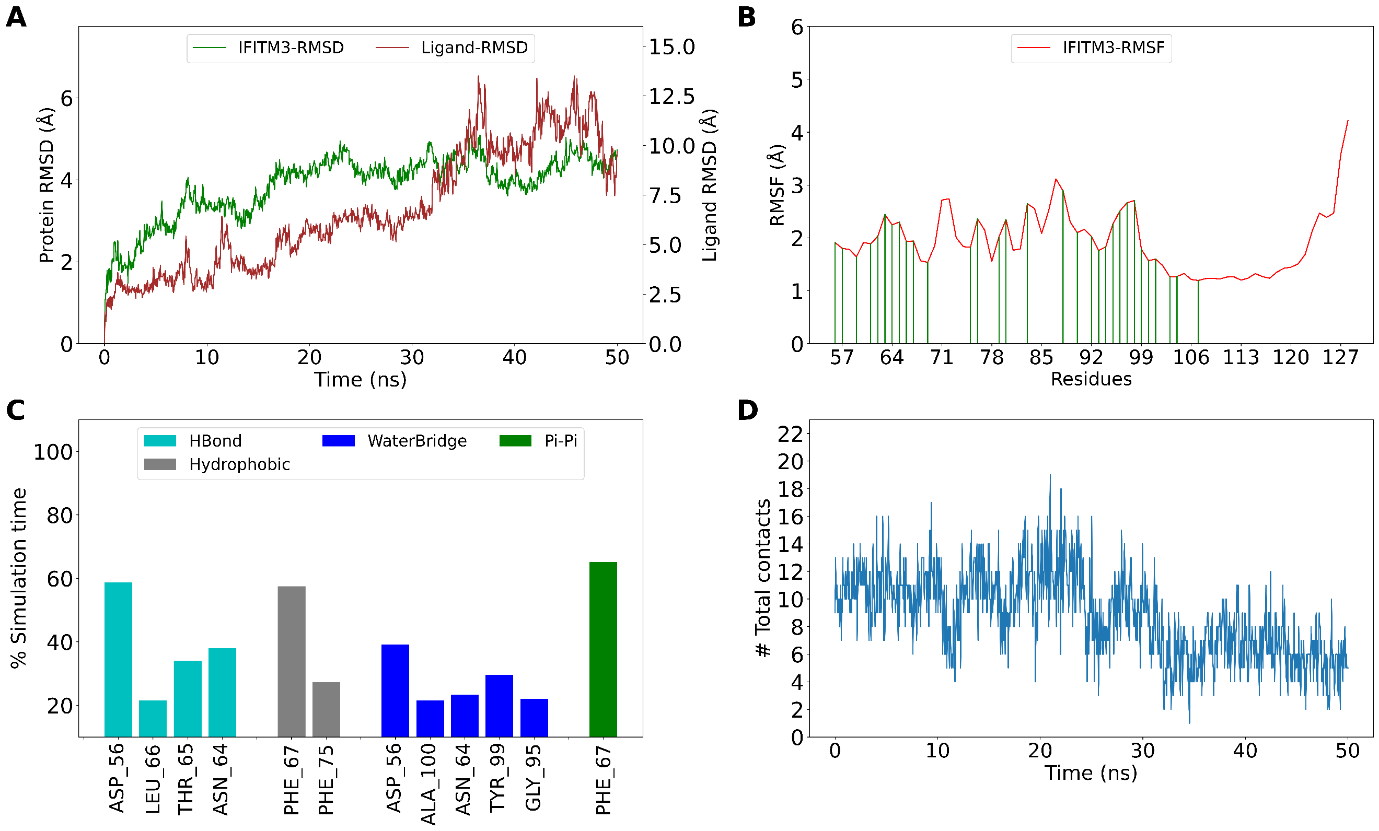
**

**Figure S26:** MD simulation of IFITM3-SN00164639 complex (**A**) RMSD of IFITM3 and SN00164639 fit on IFITM3 (Ligand-RMSD) (**B**) RMSF of IFITM3. Green lines indicate interactions with SN00164639 (**C**) Interactions between IFITM3 and SN00164639 as percentage of simulation time. Interactions that persist for more than 20% of simulation time have been shown (**D**) Total contacts (includes all interactions) between SN00164639 and IFITM3

**
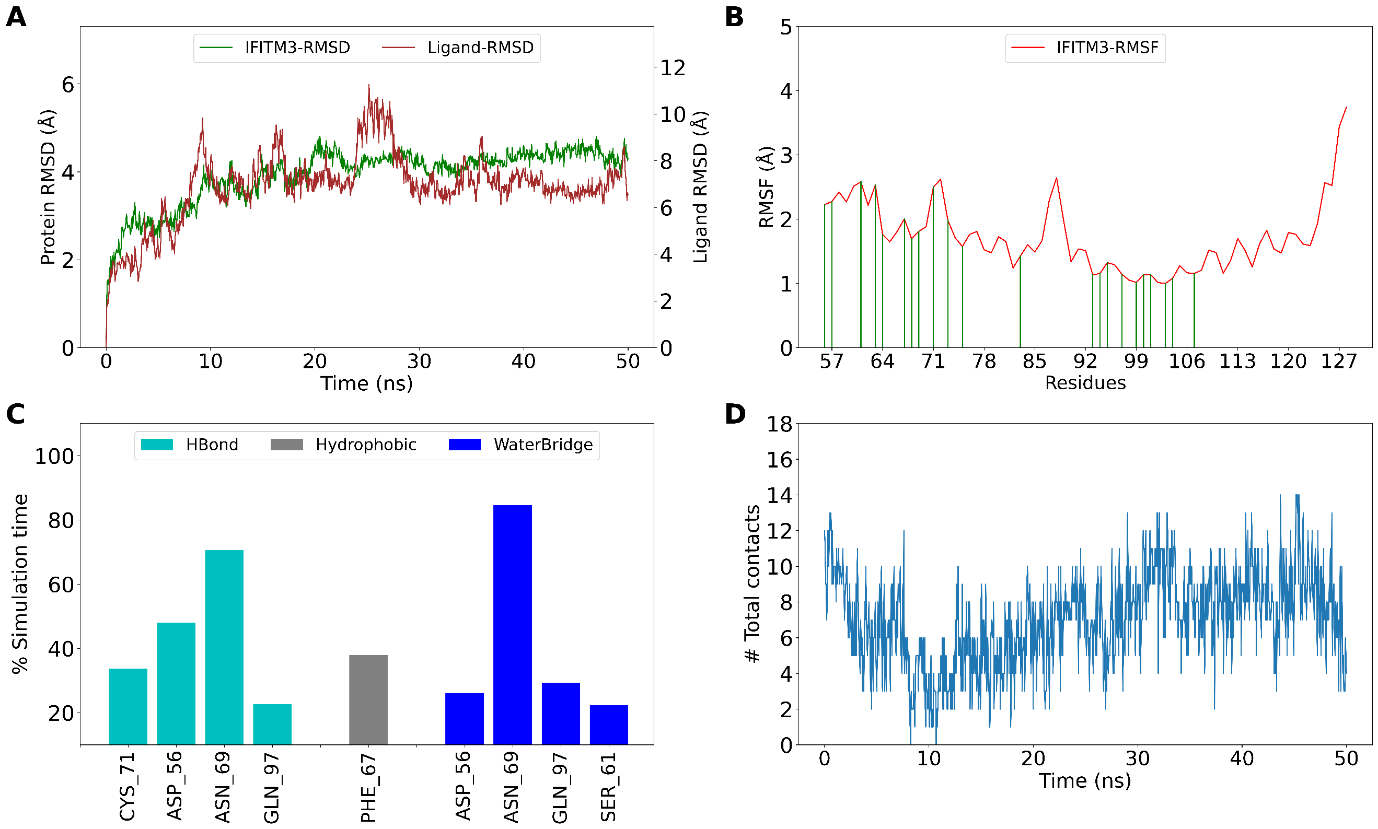
**

**Figure S27:** MD simulation of IFITM3-Periseoside D (SN00265483) complex (**A**) RMSD of IFITM3 and Periseoside D fit on IFITM3 (Ligand-RMSD) (**B**) RMSF of IFITM3. Green lines indicate interactions with Periseoside D (**C**) Interactions between IFITM3 and Periseoside D as percentage of simulation time. Interactions that persist for more than 20% of simulation time have been shown (**D**) Total contacts (includes all interactions) between Periseoside D and IFITM3

**
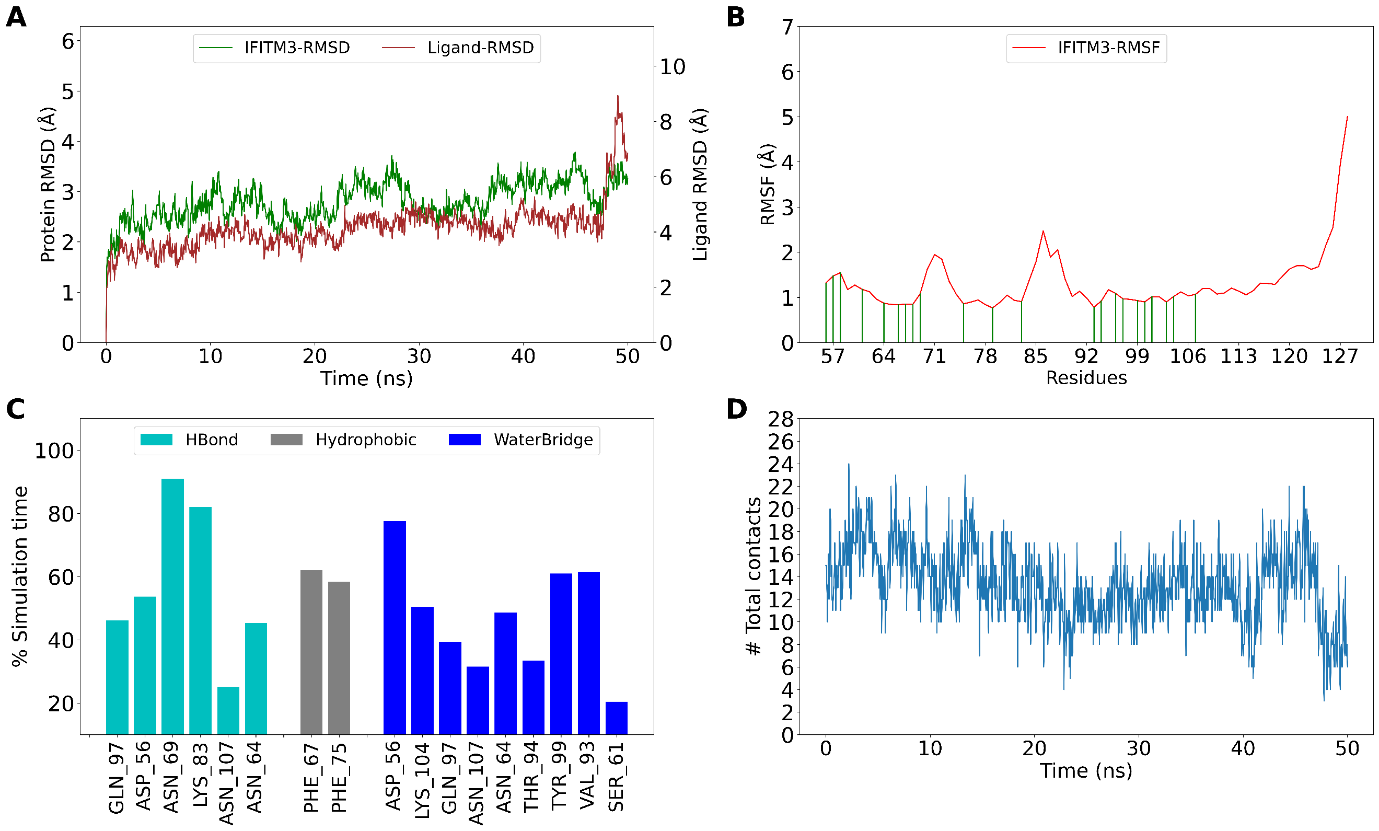
**

**Figure S28:** MD simulation of IFITM3-Pharbitic acid D (SN00274576) complex (**A**) RMSD of IFITM3 and Pharbitic acid D fit on IFITM3 (Ligand-RMSD) (**B**) RMSF of IFITM3. Green lines indicate interactions with Pharbitic acid D (**C**) Interactions between IFITM3 and Pharbitic acid D as percentage of simulation time. Interactions that persist for more than 20% of simulation time have been shown (**D**) Total contacts (includes all interactions) between Pharbitic acid D and IFITM3

**
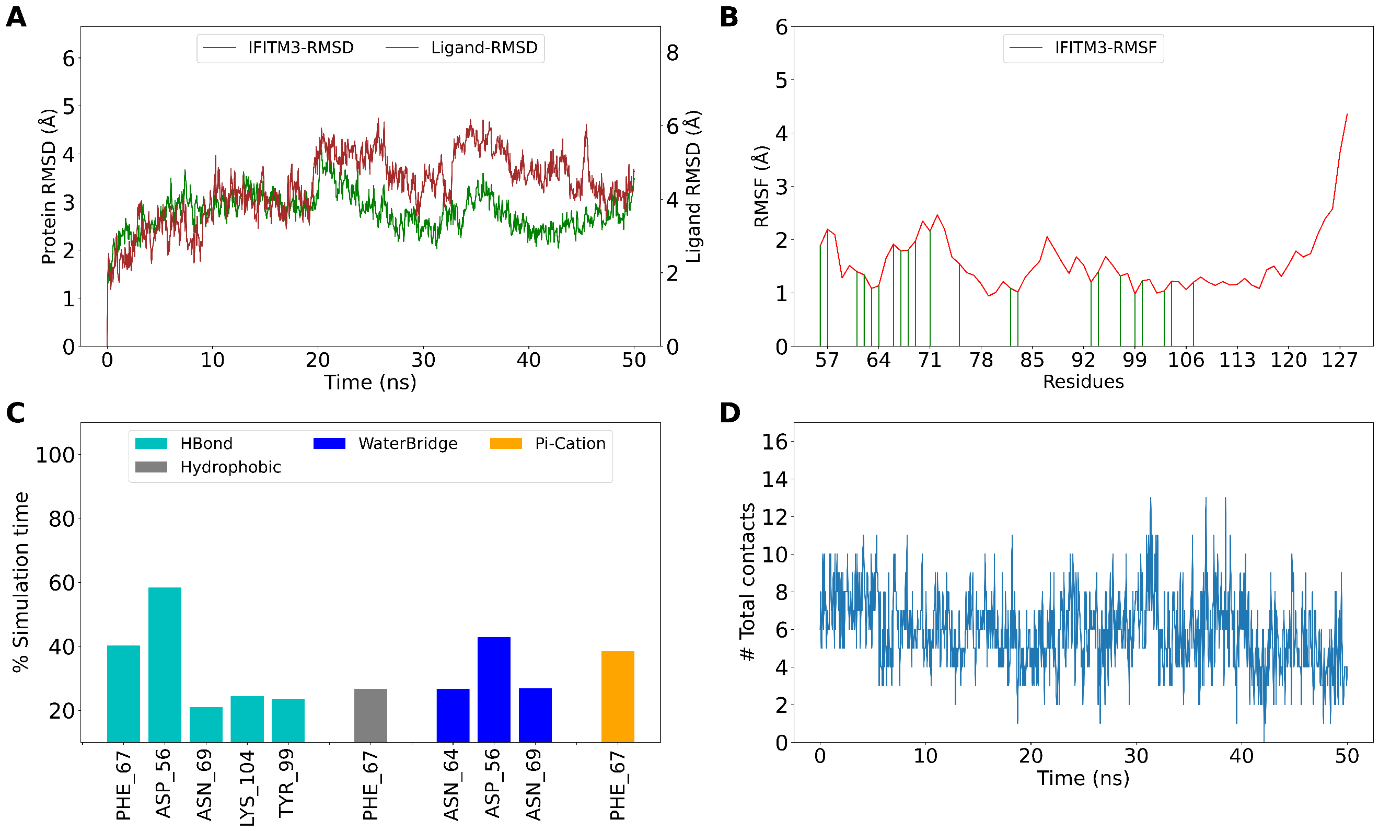
**

**Figure S29:** MD simulation of IFITM3-Pseudoceratinazole A (SN00287660) complex (**A**) RMSD of IFITM3 and Pseudoceratinazole A fit on IFITM3 (Ligand-RMSD) (**B**) RMSF of IFITM3. Green lines indicate interactions with Pseudoceratinazole A (**C**) Interactions between IFITM3 and Pseudoceratinazole A as percentage of simulation time. Interactions that persist for more than 20% of simulation time have been shown (**D**) Total contacts (includes all interactions) between Pseudoceratinazole A and IFITM3

**
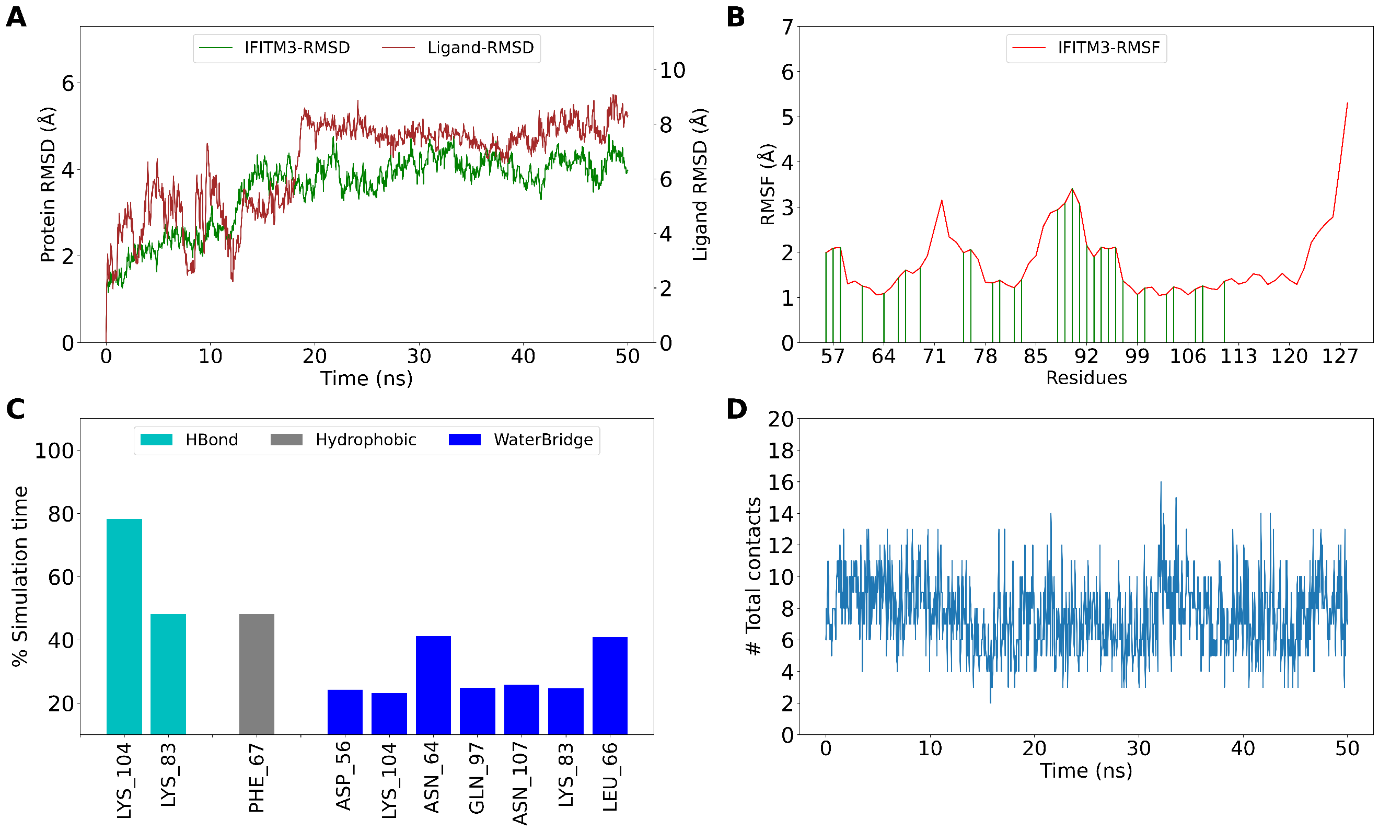
**

**Figure S30:** MD simulation of IFITM3-Parishin A (SN00379347) complex (**A**) RMSD of IFITM3 and Parishin A fit on IFITM3 (Ligand-RMSD) (**B**) RMSF of IFITM3. Green lines indicate interactions with Parishin A (**C**) Interactions between IFITM3 and Parishin A as percentage of simulation time. Interactions that persist for more than 20% of simulation time have been shown (**D**) Total contacts (includes all interactions) between Parishin A and IFITM3

**
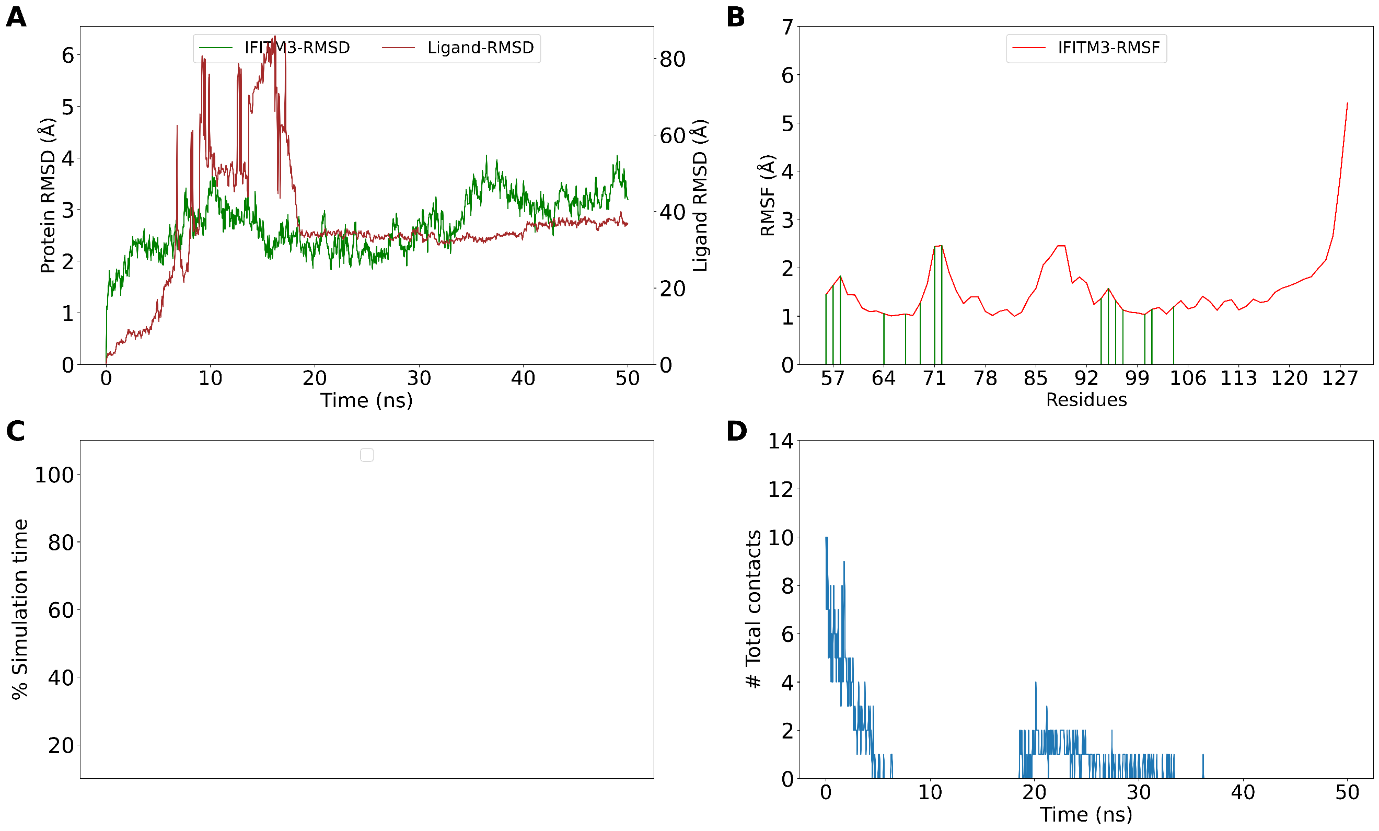
**

**Figure S31:** MD simulation of IFITM3-Hosenkoside B (SN00306006) complex (**A**) RMSD of IFITM3 and Hosenkoside B fit on IFITM3 (Ligand-RMSD) (**B**) RMSF of IFITM3. Green lines indicate interactions with Hosenkoside B (**C**) Interactions between IFITM3 and Hosenkoside B as percentage of simulation time. Interactions that persist for more than 20% of simulation time have been shown (**D**) Total contacts (includes all interactions) between Hosenkoside B and IFITM3

**
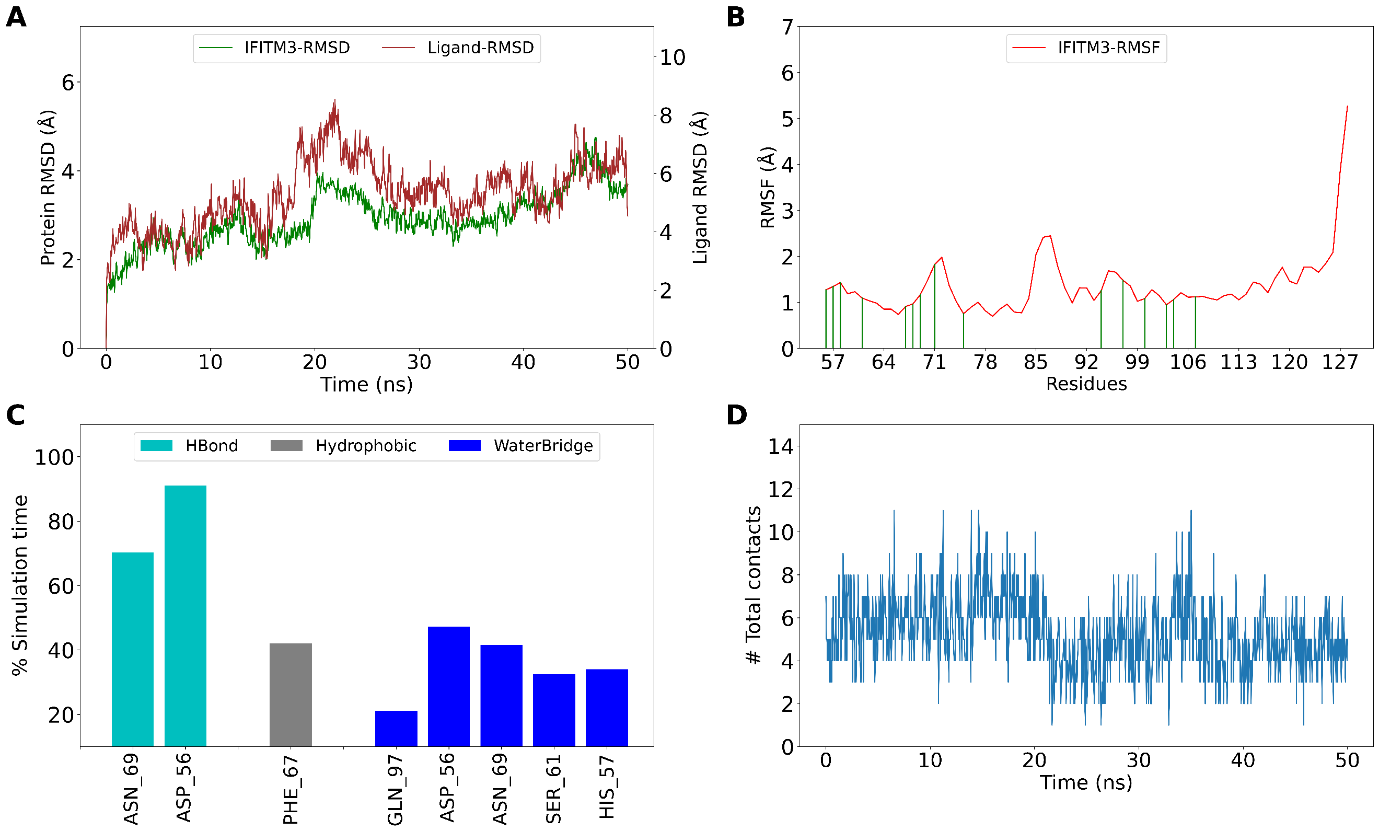
**

**Figure S32:** MD simulation of IFITM3-Anguivioside B (SN00374092) complex (**A**) RMSD of IFITM3 and Anguivioside B fit on IFITM3 (Ligand-RMSD) (**B**) RMSF of IFITM3. Green lines indicate interactions with Anguivioside B (**C**) Interactions between IFITM3 and Anguivioside B as percentage of simulation time. Interactions that persist for more than 20% of simulation time have been shown (**D**) Total contacts (includes all interactions) between Anguivioside B and IFITM3

**
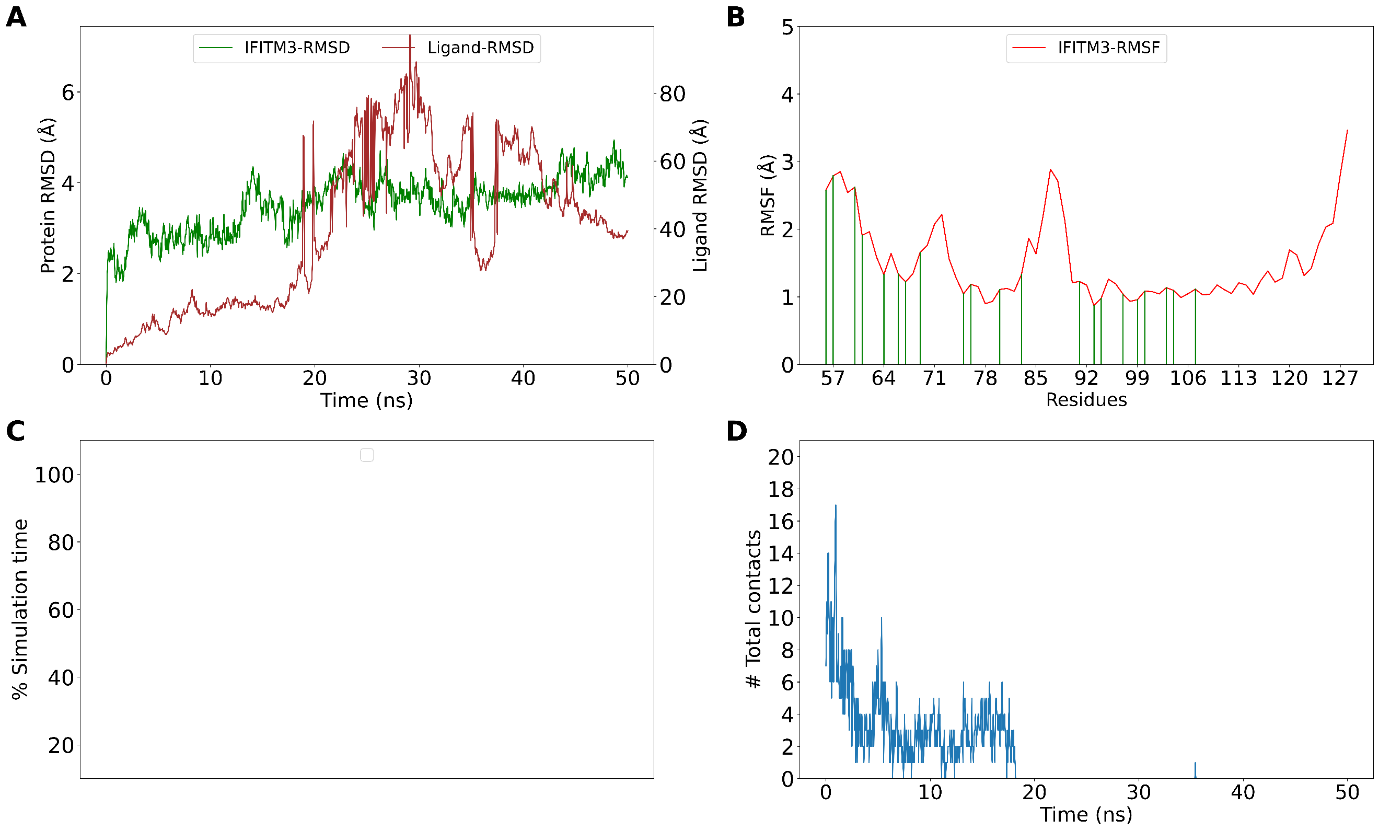
**

**Figure S33:** MD simulation of IFITM3-Disporoside D (SN00274346) complex (**A**) RMSD of IFITM3 and Disporoside D fit on IFITM3 (Ligand-RMSD) (**B**) RMSF of IFITM3. Green lines indicate interactions with Disporoside D (**C**) Interactions between IFITM3 and Disporoside D as percentage of simulation time. Interactions that persist for more than 20% of simulation time have been shown (**D**) Total contacts (includes all interactions) between Disporoside D and IFITM3

**
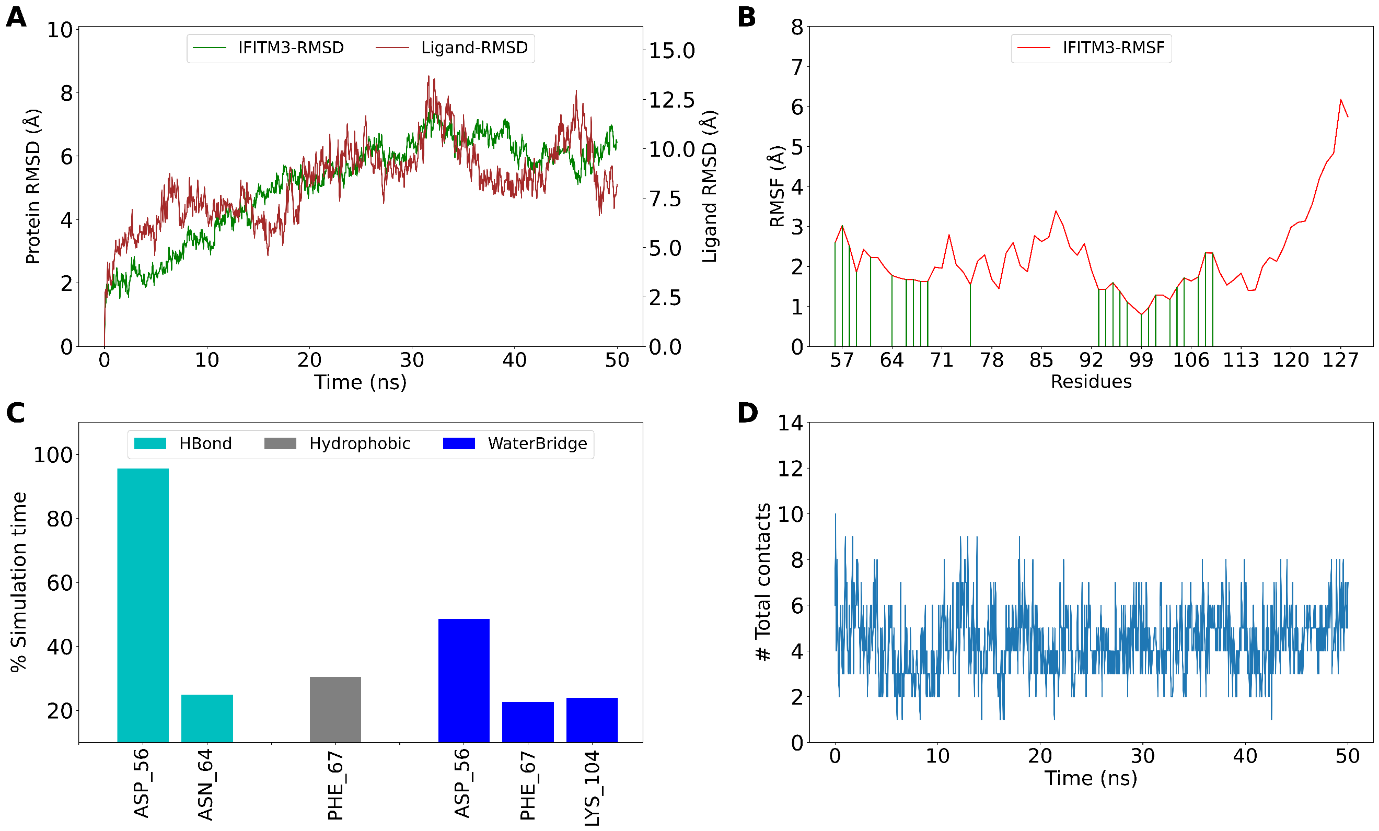
**

**Figure S34:** MD simulation of IFITM3-Broussonetine Q (SN00226238) complex (**A**) RMSD of IFITM3 and Broussonetine Q fit on IFITM3 (Ligand-RMSD) (**B**) RMSF of IFITM3. Green lines indicate interactions with Broussonetine Q (**C**) Interactions between IFITM3 and Broussonetine Q as percentage of simulation time. Interactions that persist for more than 20% of simulation time have been shown (**D**) Total contacts (includes all interactions) between Broussonetine Q and IFITM3

**
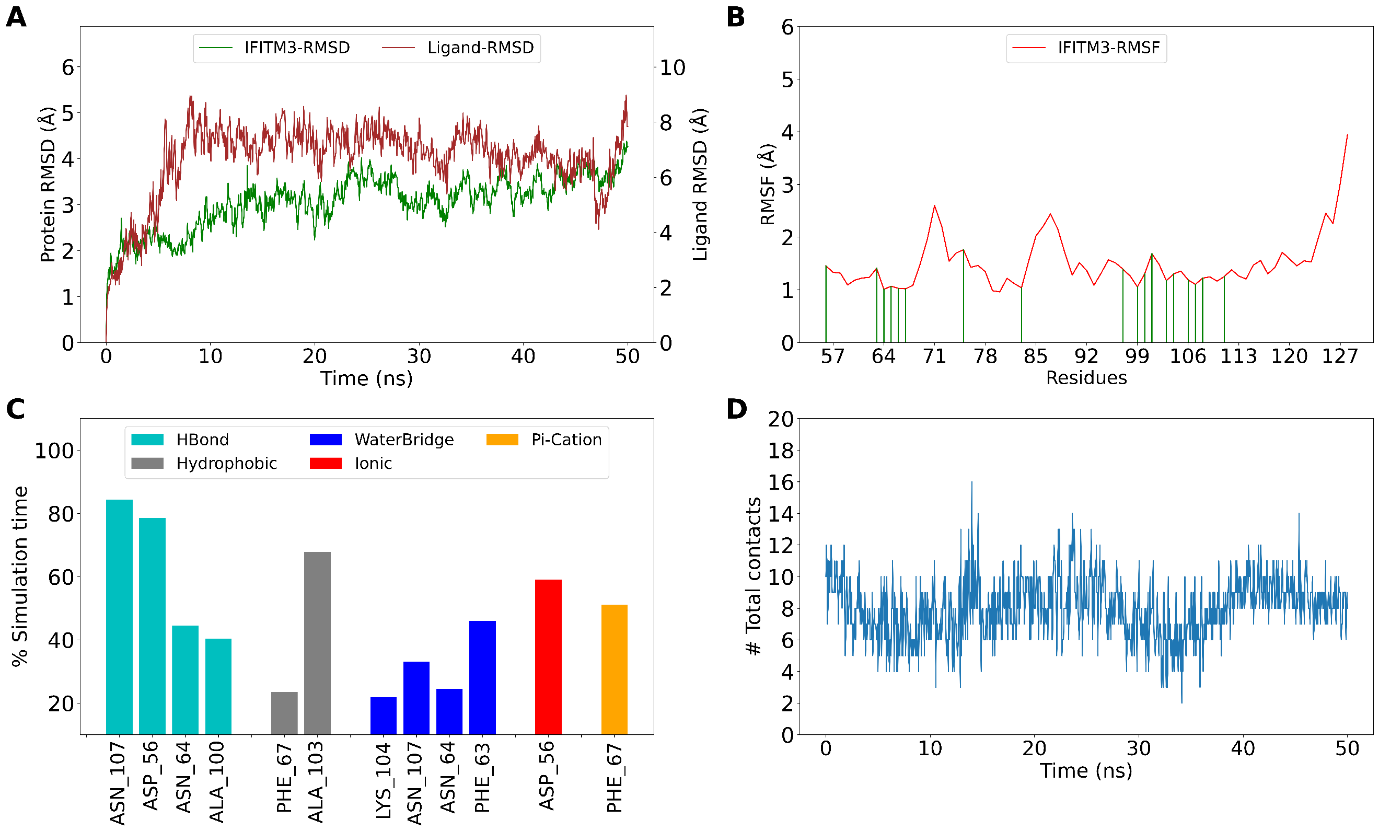
**

**Figure S35:** MD simulation of IFITM3-Nazumamide A (SN00242218) complex (**A**) RMSD of IFITM3 and Nazumamide A fit on IFITM3 (Ligand-RMSD) (**B**) RMSF of IFITM3. Green lines indicate interactions with Nazumamide A (**C**) Interactions between IFITM3 and Nazumamide A as percentage of simulation time. Interactions that persist for more than 20% of simulation time have been shown (**D**) Total contacts (includes all interactions) between Nazumamide A and IFITM3

**
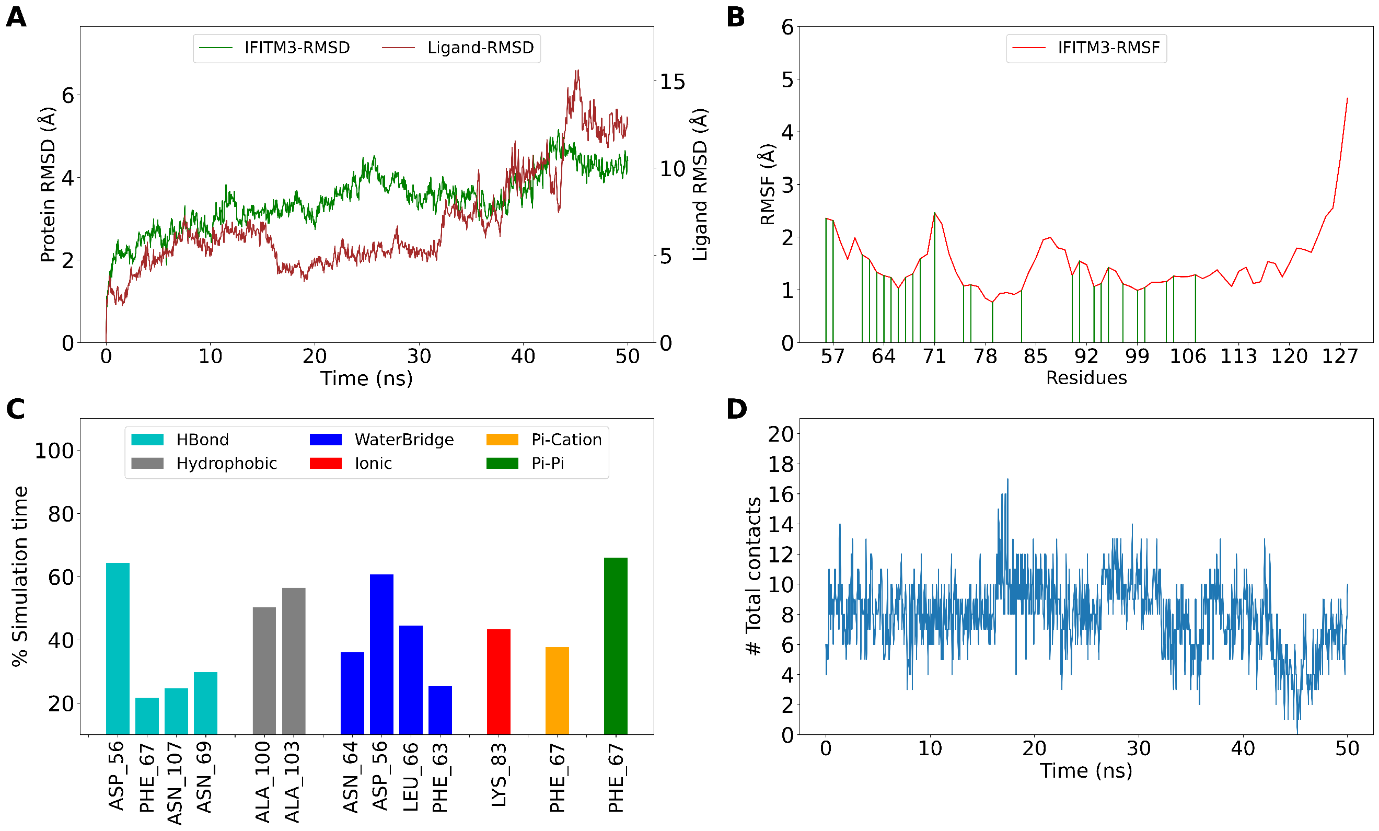
**

**Figure S36:** MD simulation of IFITM3-Kukoamine A (SN00226669) complex (**A**) RMSD of IFITM3 and Kukoamine A fit on IFITM3 (Ligand-RMSD) (**B**) RMSF of IFITM3. Green lines indicate interactions with Kukoamine A (**C**) Interactions between IFITM3 and Kukoamine A as percentage of simulation time. Interactions that persist for more than 20% of simulation time have been shown (**D**) Total contacts (includes all interactions) between Kukoamine A and IFITM3

**
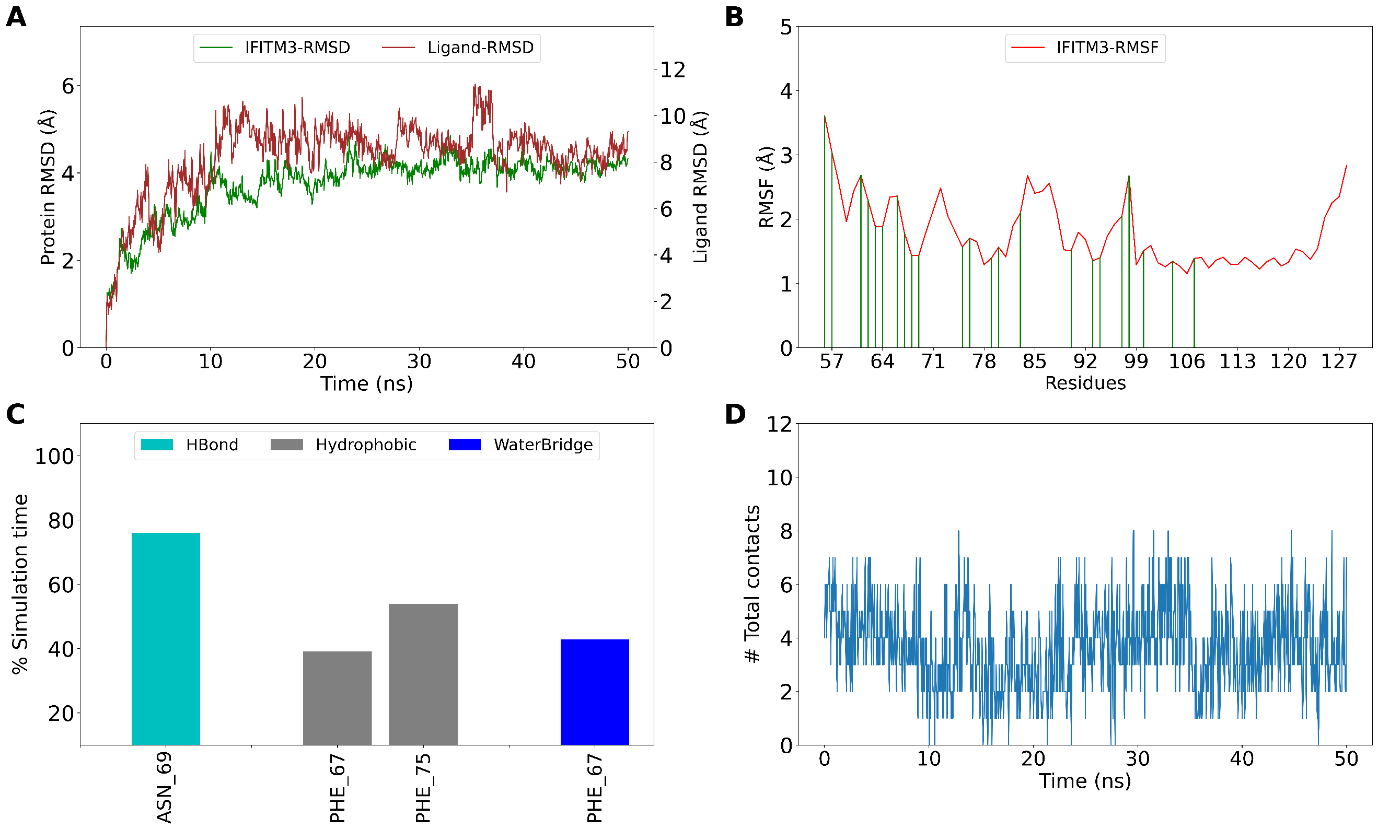
**

**Figure S37:** MD simulation of IFITM3-Rhodilunancin A (SN00304961) complex (**A**) RMSD of IFITM3 and Rhodilunancin A fit on IFITM3 (Ligand-RMSD) (**B**) RMSF of IFITM3. Green lines indicate interactions with Rhodilunancin A (**C**) Interactions between IFITM3 and Rhodilunancin A as percentage of simulation time. Interactions that persist for more than 20% of simulation time have been shown (**D**) Total contacts (includes all interactions) between Rhodilunancin A and IFITM3

**
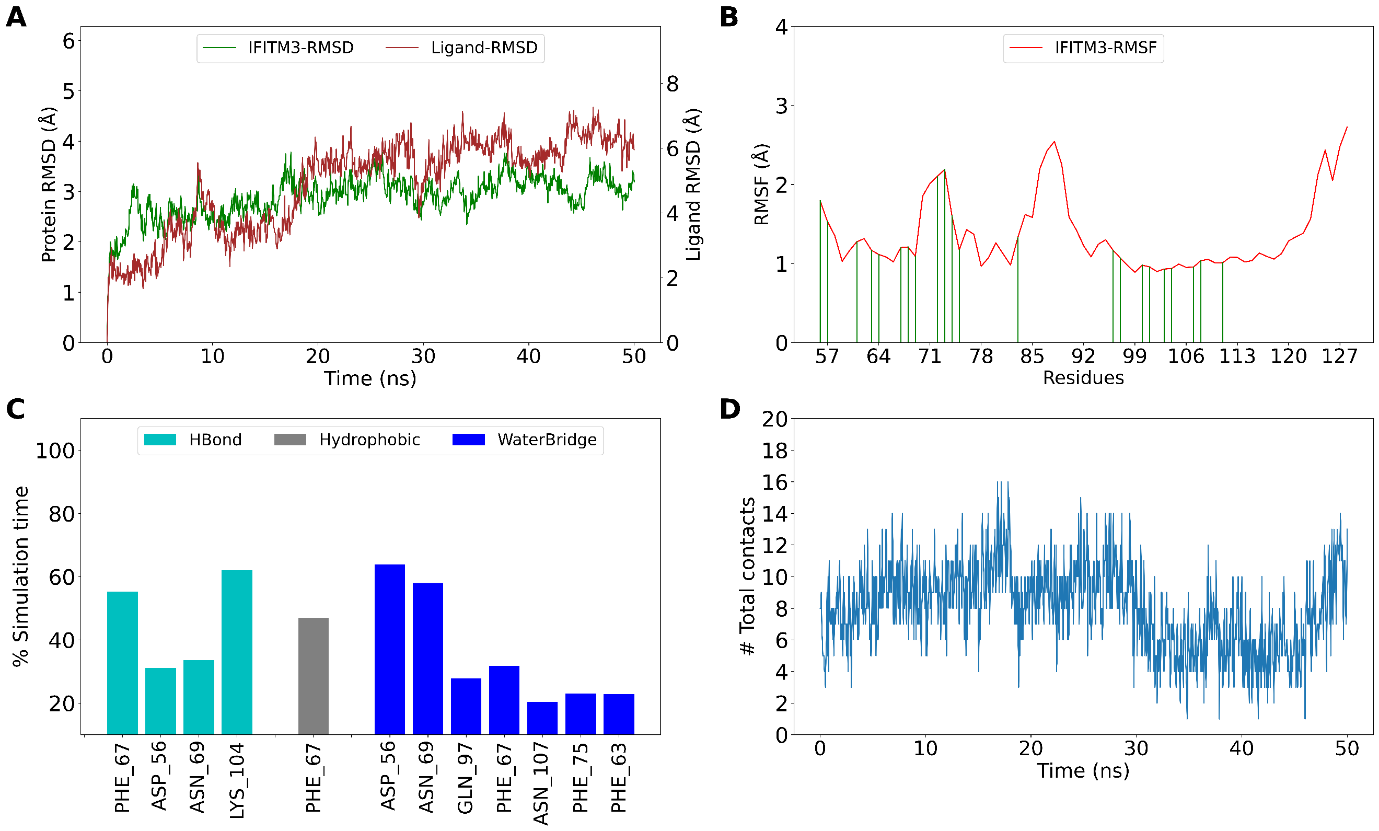
**

**Figure S38:** MD simulation of IFITM3-Caryocaroside II-12 (SN00318481) complex (**A**) RMSD of IFITM3 and Caryocaroside II-12 fit on IFITM3 (Ligand-RMSD) (**B**) RMSF of IFITM3. Green lines indicate interactions with Caryocaroside II-12 (**C**) Interactions between IFITM3 and Caryocaroside II-12 as percentage of simulation time. Interactions that persist for more than 20% of simulation time have been shown (**D**) Total contacts (includes all interactions) between Caryocaroside II-12 and IFITM3

**
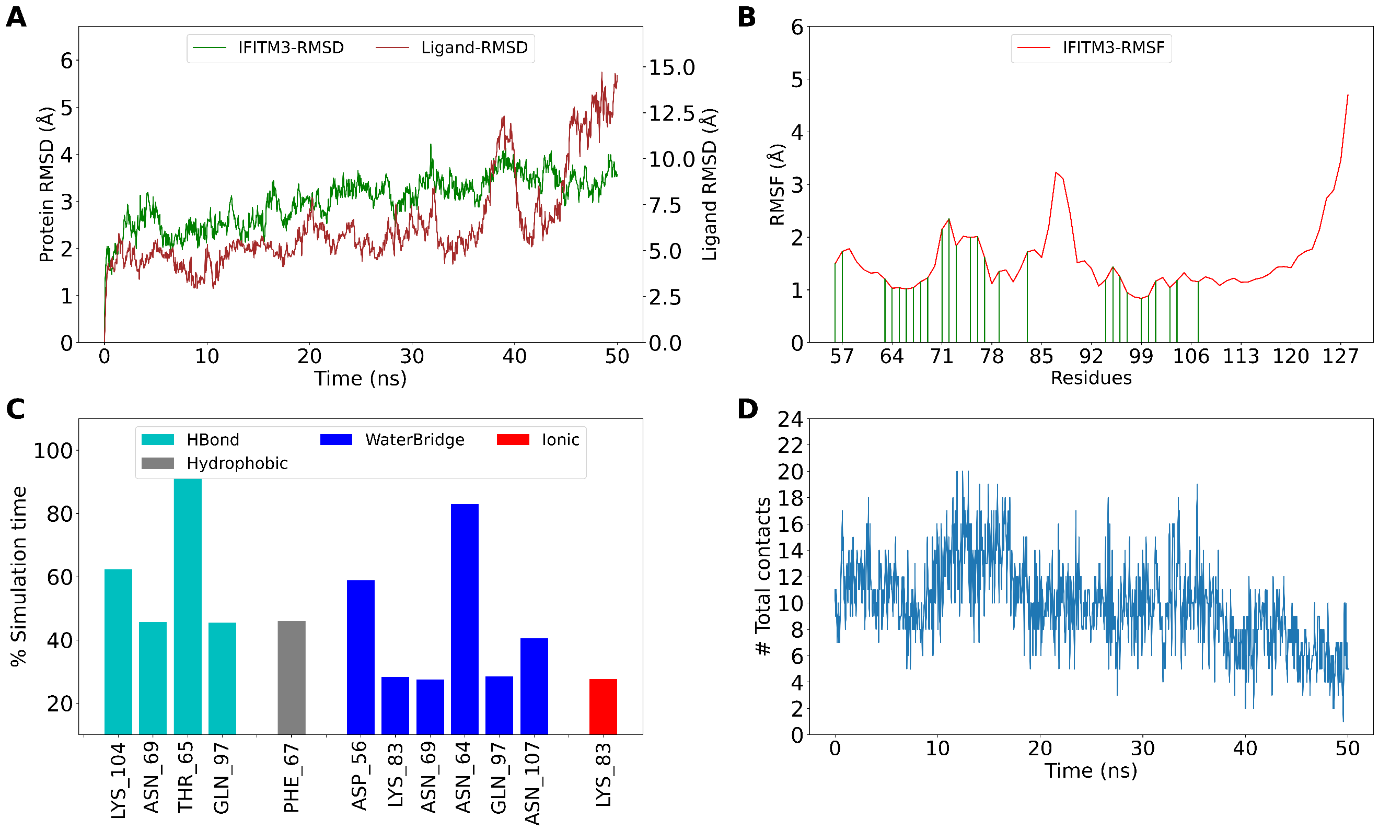
**

**Figure S39:** MD simulation of IFITM3-SN00239590 complex (**A**) RMSD of IFITM3 and SN00239590 fit on IFITM3 (Ligand-RMSD) (**B**) RMSF of IFITM3. Green lines indicate interactions with SN00239590 (**C**) Interactions between IFITM3 and SN00239590 as percentage of simulation time. Interactions that persist for more than 20% of simulation time have been shown (**D**) Total contacts (includes all interactions) between SN00239590 and IFITM3

**
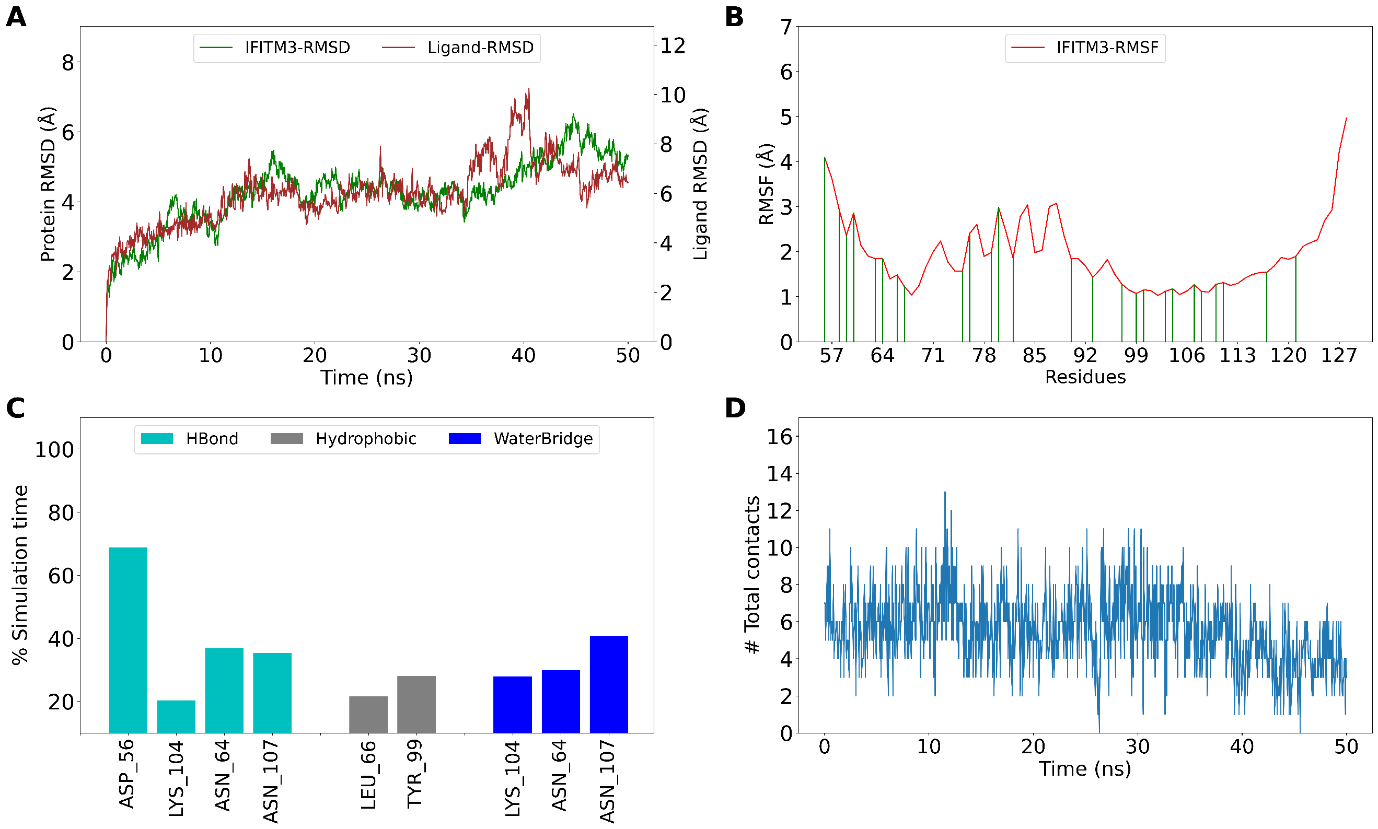
**

**Figure S40:** MD simulation of IFITM3-SN00323932 complex (**A**) RMSD of IFITM3 and SN00323932 fit on IFITM3 (Ligand-RMSD) (**B**) RMSF of IFITM3. Green lines indicate interactions with SN00323932 (**C**) Interactions between IFITM3 and SN00323932 as percentage of simulation time. Interactions that persist for more than 20% of simulation time have been shown (**D**) Total contacts (includes all interactions) between SN00323932 and IFITM3

**
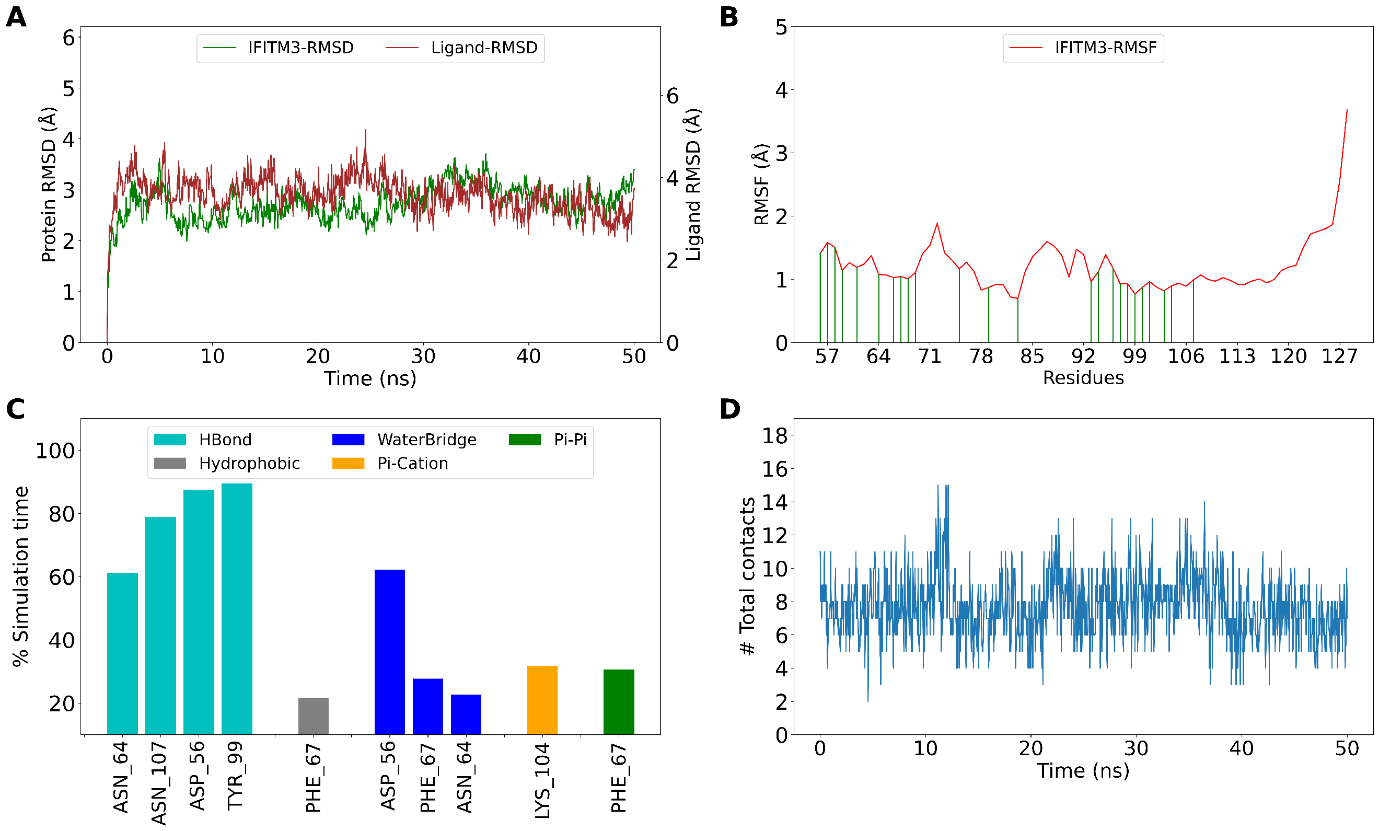
**

**Figure S41:** MD simulation of IFITM3-SN00286991 complex (**A**) RMSD of IFITM3 and SN00286991 fit on IFITM3 (Ligand-RMSD) (**B**) RMSF of IFITM3. Green lines indicate interactions with SN00286991 (**C**) Interactions between IFITM3 and SN00286991 as percentage of simulation time. Interactions that persist for more than 20% of simulation time have been shown (**D**) Total contacts (includes all interactions) between SN00286991 and IFITM3

**
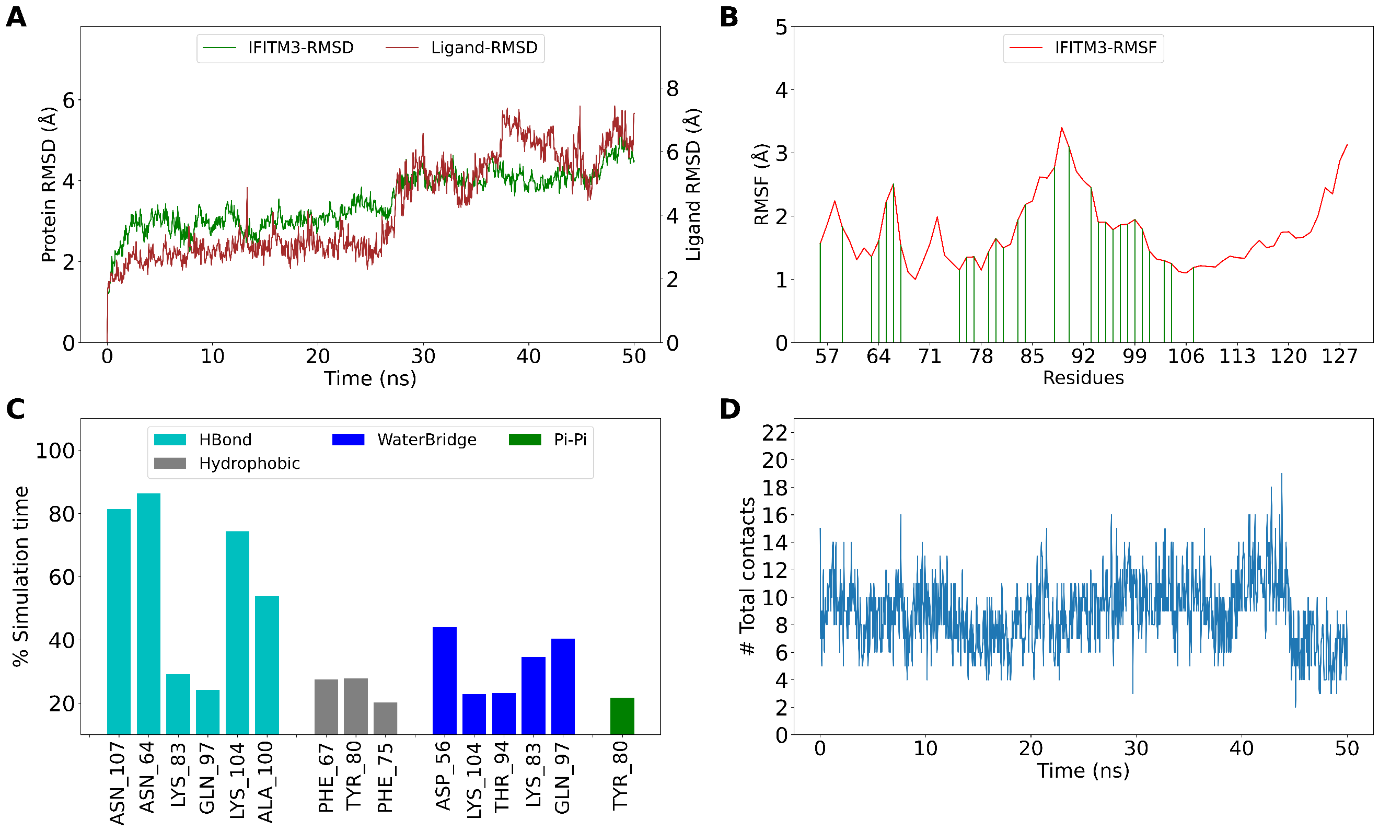
**

**Figure S42:** MD simulation of IFITM3-SN00280809 complex (**A**) RMSD of IFITM3 and SN00280809 fit on IFITM3 (Ligand-RMSD) (**B**) RMSF of IFITM3. Green lines indicate interactions with SN00280809 (**C**) Interactions between IFITM3 and SN00280809 as percentage of simulation time. Interactions that persist for more than 20% of simulation time have been shown (**D**) Total contacts (includes all interactions) between SN00280809 and IFITM3

**
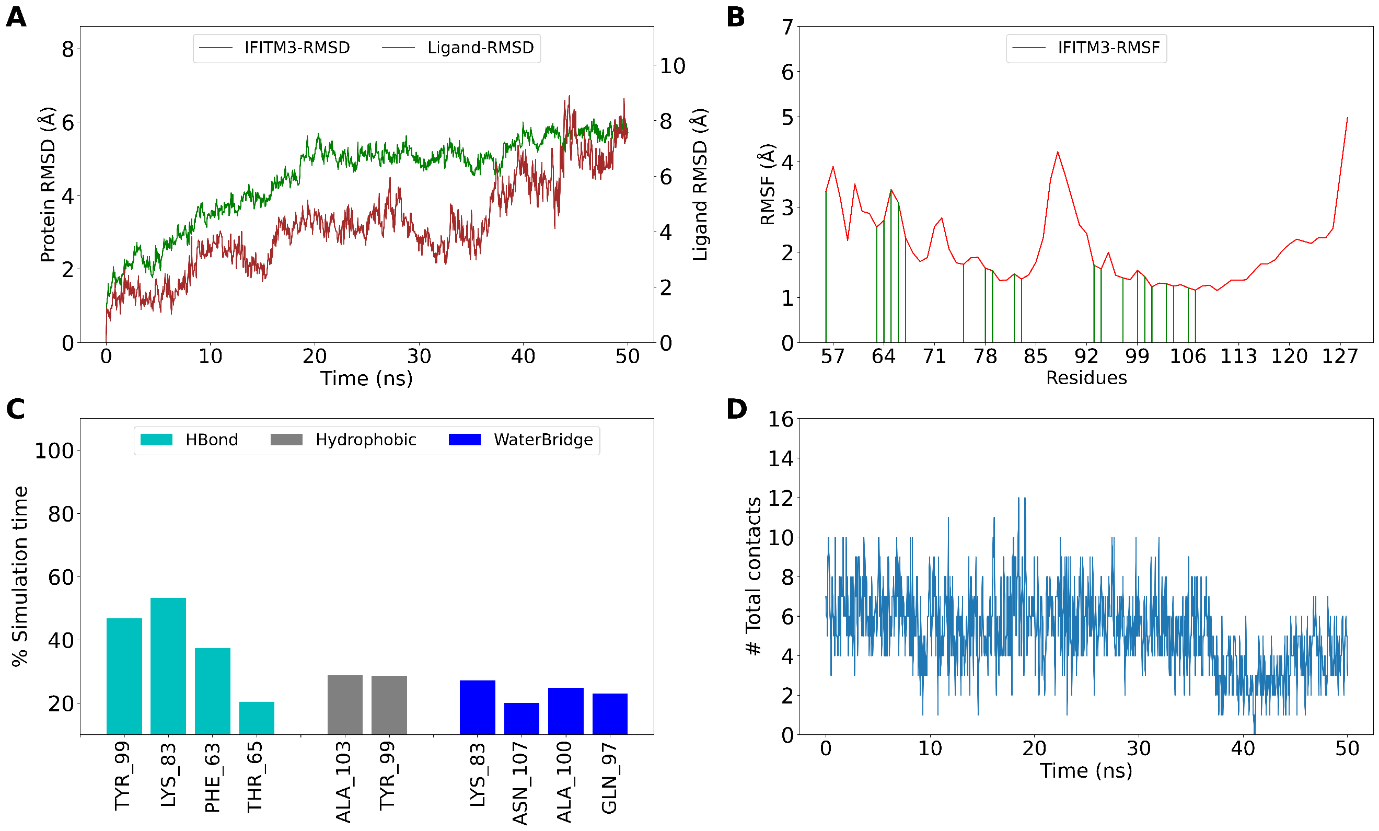
**

**Figure S43:** MD simulation of IFITM3-SN00029983 complex (**A**) RMSD of IFITM3 and SN00029983 fit on IFITM3 (Ligand-RMSD) (**B**) RMSF of IFITM3. Green lines indicate interactions with SN00029983 (**C**) Interactions between IFITM3 and SN00029983 as percentage of simulation time. Interactions that persist for more than 20% of simulation time have been shown (**D**) Total contacts (includes all interactions) between SN00029983 and IFITM3

**
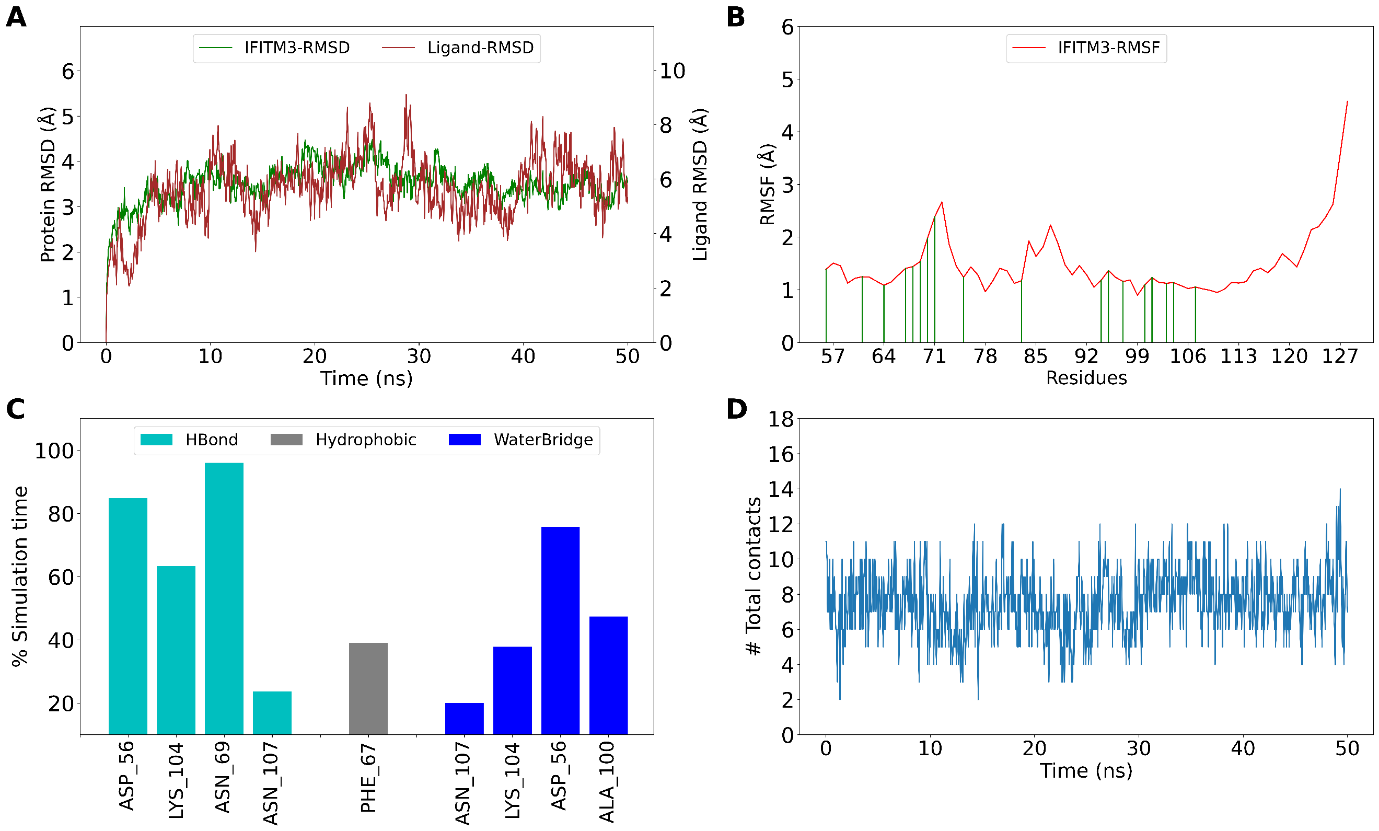
**

**Figure S44:** MD simulation of IFITM3-Amphotericin B complex (**A**) RMSD of IFITM3 and Amphotericin B fit on IFITM3 (Ligand-RMSD) (**B**) RMSF of IFITM3. Green lines indicate interactions with Amphotericin B (**C**) Interactions between IFITM3 and Amphotericin B as percentage of simulation time. Interactions that persist for more than 20% of simulation time have been shown (**D**) Total contacts (includes all interactions) between Amphotericin B and IFITM3
